# Pilot study of responsive nucleus accumbens deep brain stimulation for loss-of-control eating

**DOI:** 10.1038/s41591-022-01941-w

**Published:** 2022-08-29

**Authors:** Rajat S. Shivacharan, Camarin E. Rolle, Daniel A. N. Barbosa, Tricia N. Cunningham, Austin Feng, Noriah D. Johnson, Debra L. Safer, Cara Bohon, Corey Keller, Vivek P. Buch, Jonathon J. Parker, Dan E. Azagury, Peter A. Tass, Mahendra T. Bhati, Robert C. Malenka, James D. Lock, Casey H. Halpern

**Affiliations:** 1 Department of Neurosurgery, Stanford University School of Medicine, Stanford, CA USA; 2 Department of Neurosurgery, Perelman School of Medicine, Richards Medical Research Laboratories, Pennsylvania Hospital, University of Pennsylvania, Philadelphia, PA USA; 3 Department of Psychiatry and Behavioral Sciences, Stanford University School of Medicine, Stanford, CA USA; 4 Veterans Affairs Palo Alto Healthcare System and the Sierra-Pacific Mental Illness Research Education, and Clinical Center, Palo Alto, CA USA; 5 Department of Surgery, Stanford University School of Medicine, Stanford, CA USA; 6 Nancy Pritzker Laboratory, Department of Psychiatry and Behavioral Sciences, Stanford University School of Medicine, Stanford, CA USA; 7 Department of Surgery, Corporal Michael J. Crescenz Veterans Affairs Medical Center, PA, USA

**Keywords:** Predictive markers, Reward

## Abstract

Cravings that precede loss of control (LOC) over food consumption present an opportunity for intervention in patients with the binge eating disorder (BED). In this pilot study, we used responsive deep brain stimulation (DBS) to record nucleus accumbens (NAc) electrophysiology during food cravings preceding LOC eating in two patients with BED and severe obesity (trial registration no. NCT03868670). Increased NAc low-frequency oscillations, prominent during food cravings, were used to guide DBS delivery. Over 6 months, we observed improved self-control of food intake and weight loss. These findings provide early support for restoring inhibitory control with electrophysiologically-guided NAc DBS. Further work with increased sample sizes is required to determine the scalability of this approach.

## Abstract (web summary)

Nucleus accumbens, low-frequency, responsive deep brain stimulation improved self-control of food intake and weight loss in two patients with binge eating disorder and severe obesity

## Main

LOC eating, or the subjective sense that one cannot stop eating, is associated with binge eating—defined by the consumption of an objectively large amount of food in a short period of time accompanied by a sense of LOC^1^. LOC eating is often characterized by the loss of inhibitory control in response to appetitive cues and cravings leading to binge eating^2^. Recurrent and distressing episodes of binge eating are the key features of BED. BED is the most common eating disorder, affecting up to 3% of US adults, and is the most severe disorder associated with LOC eating based on volume of food consumed^1^. It is associated with obesity, decreased quality of life and premature mortality^3^.

Most treatments for obesity fail to address LOC eating directly, limiting the efficacy of even the most aggressive interventions such as bariatric surgery^4,5^. Clinical evidence supports a role of cravings for preferred food, or intense desires to consume specific palatable foods, before the onset of LOC and binge eating^6,7^. Particularly in individuals who are overweight or obese, food cravings have been linked with LOC among those diagnosed with BED^8^. Given this, recent studies have examined neural signals associated with food craving in the pursuit of identifying a biomarker used to trigger DBS (that is, responsive DBS (rDBS)) and inhibit onset of LOC eating when patients may be most at risk.

In the effort to identify such a craving biomarker, previous work in mice found that anticipation of a high-fat food reward was associated with increased low-frequency oscillatory power in the NAc^9^. This work supported a growing body of evidence across species reporting electrophysiological, neurochemical and functional neuroimaging activities within circuits involving the NAc that correlate with reward anticipation^10–13^ and predict consequential behavioral outcomes^14^. Using low-frequency, delta-bandpower as a biomarker to trigger delivery of a brief train of high-frequency electrical stimulation to the NAc (hereafter referred to as rDBS) resulted in substantial and lasting attenuation of binge-like eating in mice sensitized to high-fat food^9^, whereas conventional, continuous DBS appeared to lose efficacy over time^15,16^.

In the present study, we report the proof of concept in this human study designed to characterize human NAc electrophysiology of craving as it relates to LOC eating. We sought to identify changes in NAc electrophysiology associated with moments of food craving and LOC eating during controlled in-clinic behavioral tasks and to assess the generalization of this effect to LOC eating events in a naturalistic setting and outside the behavioral laboratory. Finally, we implemented rDBS triggered by NAc electrophysiology identified in behavioral and naturalistic assessments, and report here the initial results on the potential efficacy of this new intervention. The present study was performed under a US Food and Drug Administration (FDA) Investigational Device Exemption (G180079) using the NeuroPace Responsive Neurostimulation (RNS) System^17^.

## Multi-item buffet: NAc electrophysiology during in-lab LOC eating

**Fig. 1:**
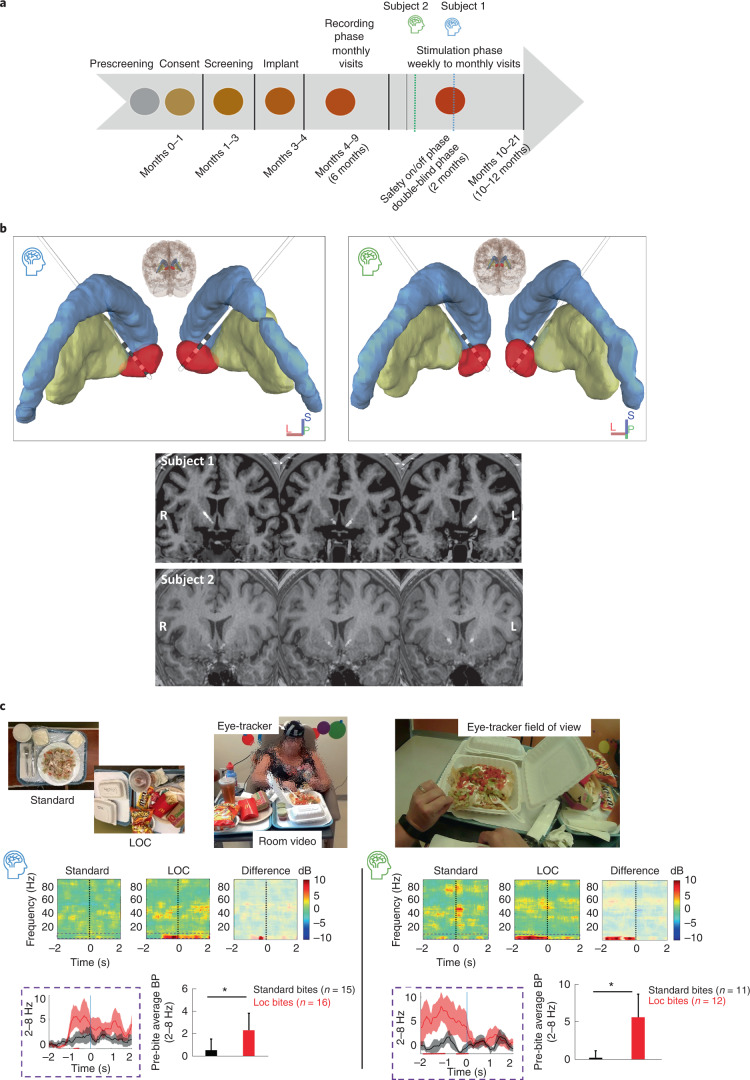
Initial characterization of human accumbens electrophysiological signal provoked by exposure to preferred food stimuli. **a**, Schematic of the multi-stage, staggered enrollment, early feasibility study design (NCT03868670). The current stage of each participant within the trial is highlighted (blue. subject 1; green, subject 2). **b**, Two quadripolar depth electrodes placed bilaterally in the NAc. Stereotactic coordinate with fGATIR and T1 images were used for direct targeting optimization of the electrode trajectory within the NAc. NAc-Red, anterior limb of internal capsule, orange; putamen, yellow; caudate, blue. Coordinates are detailed in Supplementary Table 2. **c**, The multi-item buffet of preferred foods allowing us to examine NAc electrophysiology during LOC eating as well as the preceding in-lab standardized meals. The 2 s preceding a bite was quantified during LOC versus standard meals. Both participants reported LOC after mood provocation, which correlated with significant low-frequency increases in power (2–8 Hz) during the 2 s preceding a bite when compared with standard meal bites (buffet (red): subject 1, 2.4 ± 1.5 dB, *n* = 16 bites; subject 2, 5.6 ± 3.1 dB, *n* = 12 bites; standard meal (black): subject 1, 0.6 ± 1.0 dB, *n* = 15 bites; subject 2, 0.3 ± 0.9 dB, *n* = 11 bites; Student’s *t*-test, ^*^P < 0.05). BP, bandpower. Bar graphs with error bars are presented as mean bandpower ± s.e.m.

We first describe the recording phase. In the assessment of the multi-item buffet, we investigated each subject’s LOC by modeling the at-risk environment in a controlled setting^18^. Using mood provocation (Supplementary Information), we assessed LOC (1–5 Likert severity scale) during presentation of a high-calorie buffet of the subject’s preferred foods while recording synchronized video-NAc local field potential (LFP) activity. Analogous to our pre-clinical work, we analyzed and compared bite onset during the buffet with that in standard meals. Results showed low-frequency power increases immediately before LOC eating. Specifically, increases in left ventral NAc low-frequency (2–8 Hz) power were observed for both subjects during LOC immediately preceding (within 2 s) the videoed bite-onset (Supplementary Information) power (dB) (mean ± s.e.m.): subject 1, 2.4 ± 1.5, *n* = 16 bites; subject 2, 5.6 ± 3.1, *n* = 12 bites. In contrast, increases in low-frequency power were not observed immediately before bites during standard meals (subject 1, 0.6 ± 1.0, *n* = 15 bites; subject 2, 0.3 ± 0.9, *n* = 11 bites) (Fig. 1c, Student’s *t*-test, *P* < 0.05). There were no statistical changes in any of the other recorded frequency bands in either subject (Student’s *t*-test, *P* > 0.05).

**Fig. 2:**
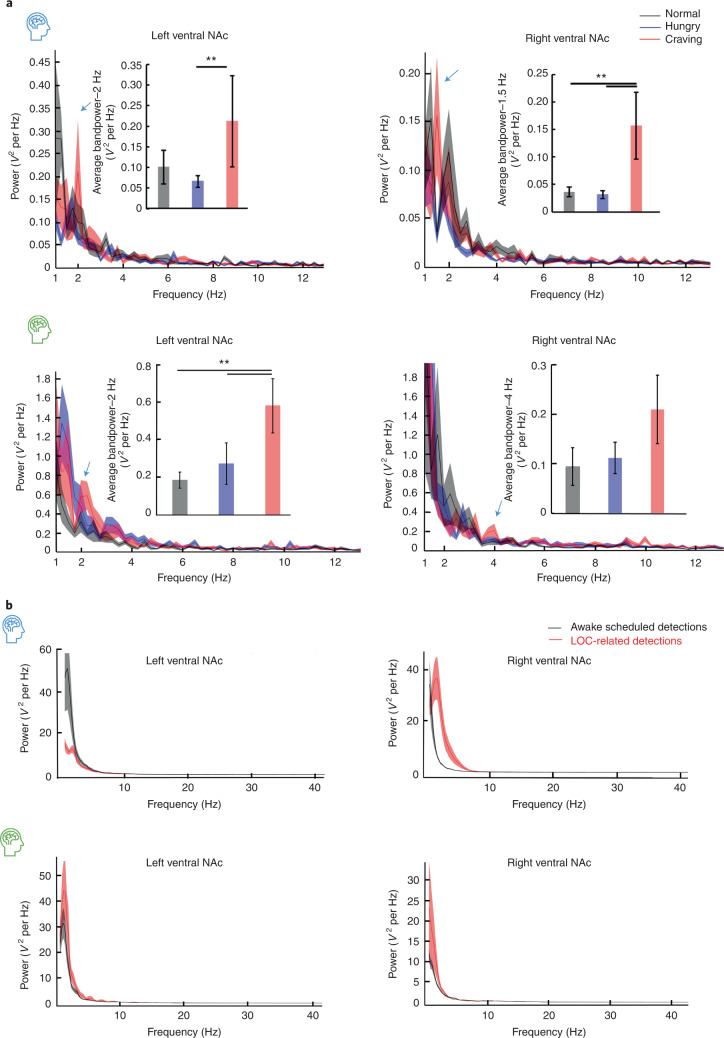
Bilateral NAc low-frequency oscillation captured during self-reported, at-risk moments for LOC eating. **a**, Both subjects reporting LOC eating events (high craving, low hunger) in the real world consistent with the diagnosis of BED, and kept a food diary describing the LOC, craving and hunger intensity. Analysis of the left and right ventral NAc showed an increase in low-frequency oscillations during LOC eating when subjects experienced craving, compared with hunger and control periods (blue arrow corresponds to frequency quantified in bandpower insert). This increase in low frequency was observed in both our subjects (craving-red trace: power (*V*^2^ (volts squared) per Hz) (mean ± s.e.m.): subject 1, left NAc: 0.21 ± 0.11, right NAc: 0.16 ± 0.06, *n* = 10 events; subject 2, left NAc: 0.58 ± 0.14, right NAc: 0.21 ± 0.07, *n* = 71 events; control-black trace: subject 1, left NAc: 0.1 ± 0.04, right NAc: 0.04 ± 0.01, *n* = 9 events; subject 2, left NAc: 0.19 ± 0.04, right NAc: 0.09 ± 0.04, *n* = 80 events; hunger-blue trace: subject 1, left NAc: 0.06 ± 0.01; right NAc: 0.03 ± 0.01, *n* = 13 events; subject 2, left NAc: 0.27 ± 0.11, right NAc: 0.11 ± 0.03, *n* = 37 events; corrected for multiple comparisons with *P* value adjustment for the three conditions using Bonferroni’s corrections (ANOVA, followed by Student’s *t*-tests comparing each state): subject 1, left NAc: *F* = 3.50, *P* = 0.04; right NAc: *F* = 4.95, *P* = 0.03; subject 2, left NAc: *F* = 5.14, *P* = 0.02, right NAc: *F* = 0.07, *P* = 0.93, ^**^P < 0.05). **b**, The detection algorithms programmed to detect low frequency in both left and right ventral NAc simultaneously. An analysis of the LFP time-locked to low frequency triggered detections captured over 8 weeks during scheduled awake and user-initiated LOC recordings and confirmed that our detection algorithms were indeed detecting changes in low-frequency power. Bar graphs with error bars are presented as mean bandpower ± s.e.m.

For ambulatory assessment, we analyzed electrophysiology acquired during real-world behavioral states (Supplementary Information) to validate the lab findings. Low-frequency power increases during LOC eating were corroborated with real-world assessments. Specifically, significantly higher low-frequency oscillatory power (Fig. 2a) in the bilateral ventral NAc was found during subject-reported LOC eating events (craving-red trace power (*V*^2^ per Hz) (mean ± s.e.m.): subject 1, left NAc: 0.21 ± 0.11, right NAc: 0.16 ± 0.06, *n* = 10 events; subject 2, left NAc: 0.58 ± 0.14, right NAc: 0.21 ± 0.07, *n* = 71 events) when compared with control periods (control-black trace, subject 1, left NAc: 0.1 ± 0.04, right NAc: 0.04 ± 0.01, *n* = 9 events; subject 2, left NAc: 0.19 ± 0.04, right NAc: 0.09 ± 0.04, *n* = 80 events) and periods of hunger (hunger-blue trace, subject 1, left NAc: 0.06 ± 0.01, right NAc: 0.03 ± 0.01, *n* = 13 events; subject 2, left NAc: 0.27 ± 0.11, right NAc: 0.11 ± 0.03, *n* = 37 events) (Fig. 2a, one-way ANOVA, subject 1, left NAc: *F* = 3.50, *P* = 0.04, right NAc: *F* = 4.95, *P* = 0.03; subject 2, left NAc: *F* = 5.14, *P* = 0.02, right NAc: *F* = 0.07, *P* = 0.93). Consistent with the in-clinic tasks, there were no differences in any other frequency band during at-risk moments in the ambulatory setting.

For each subject, we programmed the device to detect brief increases in low-frequency activity in both the left and the right ventral NAc. To confirm that the signal being detected was in the low-frequency range, we analyzed the power spectra of the NAc LFP activity in the 5 s before detection and found that the area detectors (Supplementary Information) were detecting low-frequency activity in the left and right ventral NAc (Fig. 2b). For this analysis, we compared detection made in stored LFPs during reported LOC eating events and awake events. For subject 1, increased low-frequency power compared with baseline NAc LFP signal (average 2-min window) was identified in 74.4% (67/90) of reported LOC eating event detections and 63.2% (84/133) of the awake detections (*χ*^2^(1,*n* = 223) = 24.54, *P* < 0.05). For subject 2, increased low-frequency power was identified in 76.9% (30/39) reported LOC eating event detections and 45.8% (22/48) awake detections (*χ*^2^(1,*n* = 87) = 14.82, *P* < 0.05).

**Extended Data Fig. 1:**
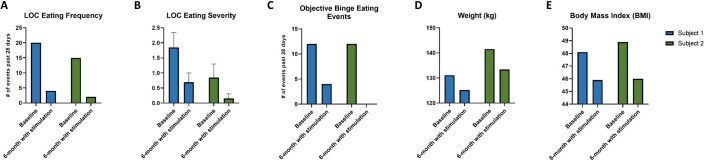
6-Month rDBS outcomes. Bilateral stimulation has been well tolerated by both subjects (~1.5μC/cm^2^ charge density, 10 s duration). Both subjects report an increased sense of control specific to food cravings and eating-related behaviors. Here, behavior outcomes are reported at 6-months with stimulation (rDBS) compared to pre-surgery baseline. **A)** For both subjects, LOC frequency decreased with stimulation (*Subject 1: 16-point decrease; Subject 2: 13-point decrease*). **B)** With LOC episode severity, both subjects showed decrease in severity from baseline to 6-months with stimulation (*Subject 1: Baseline* = *1.84* + */−0.69 (n* = *13 samples), 6-month* = *0.69* + */−0.053 (n* = *13 samples), student’s t-test, p* = *0.05; Subject 2: Baseline* = *0.84* + */−0.15 (n* = *13 samples), 6-month* = *0.15* + */−.09 (n* = *13 samples), student’s t-test, p* = *0.09*). Further, both subjects also experienced decreases in **C)** For both subjects, Objective Binge Eating events (OBE) decreased with stimulation (*Subject 1: 8-point decrease; Subject 2: 12-point decrease*). **D)** Weight (kg, %) and **E)** BMI (*Subject 1: Weight* = *−5.9* *kg, 4.5%, BMI* = *−2.2; Subject 2: Weight* = *−8.2* *kg, 5.8%, BMI* = *−2.9*). Bar graphs with error bars are presented as mean band power + /- S.E.

Considering the stimulation phase, both subjects reported an increased sense of self-regulation and control over food intake specific to cravings and related eating behavior. Furthermore, both subjects showed a decrease in the reported frequency of LOC eating events from baseline to 6 months post-stimulation (that is, the primary endpoint), as assessed by the Eating Disorder Examination (EDE), and LOC severity, as assessed by the Eating Loss of Control Scale (ELOCS), across the 28-d period during the baseline month compared with 6 months after the stimulation month (LOC frequency: subject 1 = 80% decrease; subject 2 = 87% decrease; LOC episode severity: subject 1: 9-point improvement (*P* = 0.09); subject 2: 15-point improvement (*P* = 0.05)) (Extended Data Fig. 1a,b). Notably, by the end of the 6-month follow-up period, subject 1 exhibited substantial improvement in BED severity, whereas subject 2 no longer met criteria for BED (that is, fewer than average of four binge eating events per month over the prior consecutive 3 months for no more BED diagnosis), which met our primary endpoint (Extended Data Fig. 1c). Corroborating their subjective reports (Extended Data Fig. 1), 6-month outcomes showed a decrease in body weight (kg and percentage reduction) and body mass index (BMI) for both subjects: subject 1 = −5.9 kg, −4.5% and −2.2 kg m^−2^, respectively; subject 2 = −8.2 kg, −5.8% and −2.9 kg m^−^^2^, respectively (Extended Data Fig. 1d,e). Month-to-month reports of Ecological Momentary Assessment (EMA), ELOCS ratings and weight (kg)/BMI are reported for both subjects in Supplementary Figs. 3.1–3.6.

## Discussion

The present study identified NAc low-frequency oscillatory power as a signal associated with LOC craving, and then implemented this biomarker to guide rDBS delivery in two subjects with BED and severe obesity. In the recording phase, in-lab assessments implicated NAc low-frequency signaling during naturalistic LOC eating. The generalizability of this signal to real-world settings was then corroborated by our finding that low-frequency oscillatory power was increased during real-world LOC eating events compared with non-LOC events. In the stimulation phase, 6 months of bilateral NAc rDBS triggered by low-frequency power was found to improve LOC eating, as well as reduce body weight and BMI. Optimization of stimulation parameters is still ongoing in both subjects, and four additional subjects are expected to be implanted after a supplement approval to our investigational device exemption. The present study was a safety and feasibility assessment and LOC was the primary endpoint. As such, we did not control for changes in lifestyle or diet. However, we used single-blind subjects during stimulation to rule out stimulation-induced side effects and placebo effects. Moreover, subjects 1 and 2 both reported making healthier choices around food and having interest in being more physically active, although neither patient enrolled in any regular exercise or diet program during this 6-month follow-up. Therefore, we have found that, by ameliorating LOC through NAc stimulation, we’ve helped patients with severe obesity lose weight without providing them instruction or guidance on a dietary plan. This supports the stimulation-induced restoration of control in these subjects being the underlying mechanism of BMI improvement.

We encountered early challenges when capturing LOC eating events in the real world. A training period was necessary before surgery for both subjects to learn to identify and document their LOC eating behaviors. This involved having a psychiatrist (D.S.) with expertise in obesity and eating disorders discuss with each patient her personal understanding of LOC eating. As we report (Supplementary Information), although sensitivity of low-frequency detections to LOC eating was high, low-frequency oscillations in the NAc were not always specific to food craving and LOC eating compared with non-LOC eating events. Ongoing work seeks to optimize detection algorithms and improve the sensitivity and specificity of rDBS for LOC eating. Furthermore, real-world LOC electrophysiology detected from ambulatory recordings was specific to bilateral, ventral NAc delta (2–4 Hz), whereas in-lab experiments found effects in both delta and theta (2–8 Hz) and were limited to the left ventral NAc. We believe this inconsistency to be grounded in the methodological variance between the in-lab buffet task procedures and the ambulatory data acquisition procedures. The NAc signal acquired during the in-lab buffet task NAc was time-locked to bites during an LOC eating episode (2 s preceding a bite), whereas the ambulatory data were time-locked to moments of craving preceding an LOC eating episode (60 s preceding a user-initiated magnet swipe). Although cravings are a relevant and important precedent to LOC eating, they capture a signal that is characteristically different from the signal during eating itself. Furthermore, there is a substantial difference in the duration captured (and averaged over) between the two types of data acquisition, which could certainly contribute to the variance in results. Secondarily, the in-lab buffet task captures an LOC eating episode that is clinically provoked using emotional content, whereas the ambulatory data are acquired during all LOC eating episodes. Real-world LOC can be provoked for a number of reasons and by a combination of factors not entirely captured by mood provocation in the lab. Therefore, it is possible that the specificity of in-lab procedures during the buffet task did not capture all dimensions of LOC eating that the ambulatory data did. We also note that, although the frequencies within which we found our effects contained the delta signal identified in mice^9^, the effects from in-lab testing were broader and inclusive of theta frequencies. It’s important to mention that it is difficult to disambiguate the clinical value of the presence of low-frequency peaks, compared with the frequency of low-frequency peaks, in predicting LOC eating events. We believe that this is an interesting scientific query that would be better answered by more targeted analyses of low-frequency power and LOC eating. Importantly, one difficulty with the low-frequency biomarker signal is its presence during normal physiological processes such as sleep^19,20^. To account for detection and stimulation during sleep, we limited rDBS delivery to awake hours (7am–10pm). Finally, the upfront cost of implantable devices is high; thus long-term follow-up of LOC eating as well as BMI beyond the study period will be necessary to assess societal cost-effectiveness of this intervention based on our decision analyses^21^.

In conclusion, NAc rDBS improved LOC eating frequency and severity in two patients with BED and severe obesity. These findings were associated with weight loss even during this early follow-up period, suggesting that patients can lose weight without instruction to change their diet or physical activity (efforts that are often unsuccessful). This is a testament to the potential clinical impact of this new intervention and supports continued study in this FDA-guided, human, early feasibility trial.

## Methods

This research complies with and was approved by Stanford’s Institutional Review Board (IRB-46563). The study protocol for this clinical trial (trial registration no. NCT03868670) is publicly available as a supplement to this manuscript. Data presented in this manuscript can be requested within reason by contacting the corresponding author (see Data availability for additional details). As previously described, the clinical trial is a multi-stage early feasibility study with staggered enrollment (Fig. 1a, see protocol paper for more details^17^). All participant information, magnetic resonance imaging (MRI), computed tomography (CT), LFP and behavior data were collected under a Stanford University IRB-approved protocol (IRB no. 46563, NCT03868670).

### Prestudy procedures

#### Subjects

Two adult women with BED and treatment-refractory, severe (grade III) obesity, despite bariatric surgery, were recruited for the present study. The first participant (subject 1), a 45-year-old white woman with a BMI of 46 kg m^−2^, underwent roux-en-Y gastric bypass (RYGB) in 2005 and experienced an initial weight loss of 52.16 kg. However, the participant gradually regained the weight. At the time of enrollment, she was back to her pre-RYGB weight and met DSM-V criteria^1^ for BED, reporting at least five LOC eating bouts per week, with three LOC eating events per week as well. At the time of enrollment, she had co-morbities of neoplasm, lower back pain, kyphoscoliosis/scoliosis, hypertension, esophageal reflux, dyslipidemia, complicated migraine and anxiety. The second participant (subject 2), a 56-year-old white woman with a BMI of 47 kg m^−2^, underwent RYGB in 2005 and initially lost 68.95 kg. Subject 2 maintained this weight loss for 6 years; however, she regained the weight in 2009 while both caring for an ill family member and recovering from a car accident. At the time of study enrollment, she was within 9% of her pre-RYGB weight and she reported LOC eating four times a week and met criteria for BED as well. At the time of enrollment, she had a migraine co-morbidity. Both subjects reported severe cravings related to emotional and/or stress-related triggers that led to LOC eating. In addition to the RYGB, both subjects tried numerous other weight-loss strategies including exercise, dieting, support groups and medication. as was required by our enrollment criteria.

The study was approved by Stanford’s IRB (IRB-46563) (see Supplementary Information for participant characteristics) and informed consent was obtained from all subjects. Neither participant was compensated for their participation.

#### Study design

Designed with a staggered enrollment, each subject progressed through the study stages shown in Fig. 1a. Both subjects underwent stereotactic implantation of bilateral depth electrodes, each with four contacts. The two distal contacts of the DBS leads were positioned in the NAc, verified by both indirect and direct targeting strategies noted earlier, with the two more proximal contacts traversing the anterior limb of the internal capsule (Fig. 1b and Supplementary Table 2)^22^. The preoperative MRI anatomical images were co-registered to a post-surgical CT scan using advanced normalization tools (ANTs^23^) for electrode localization^24^. The electrode artifacts from the co-registered postoperative CT, together with the preoperative MRI and atlas-based regions of interest, were then loaded in DSI Studio for three-dimensional rendering (https://dsi-studio.labsolver.org), as presented in Fig. 1b.

Implantation → Recording phase (6 months) → Stimulation phase (12 months; see Fig. 1a).

#### Responsive DBS system

The closed-loop intracranial stimulation system used here for rDBS was a brain RNS System (NeuroPace, Inc.) currently approved by the US FDA for the treatment of adults with medically refractory focal-onset seizures. The system consists of two intracranial recording and stimulating electrodes connected to a neurostimulator implanted in the skull. The neurostimulator continuously monitors electrographic signals and detects abnormal electrophysiological activity to trigger brief bursts of electrical stimulation. The RNS System stores hourly counts of detected events as well as multiple 1- to 3-min snapshots of LFP activity, which are uploaded to a computer and server where they can be analyzed and reviewed. This system allows the opportunity to collect and examine electrographic activity of the NAc in a subject’s naturalistic environment during vulnerable periods of craving that precede LOC eating events.

#### Stereotactic targeting, device implantation and recording configuration

After screening by a multidisciplinary team, the subjects underwent surgical placement of the cranial neurostimulator attached to two depth leads (four contacts each, 3.5-mm electrode spacing) (NeuroPace, Inc.) targeting the NAc bilaterally (Fig. 1b). Direct targeting of the NAc using Fast Gray Matter Acquisition T1 Inversion Recovery (fGMATI) MRI was implemented. Due to the investigative nature of the present study, the neurostimulator (250-Hz sampling rate) was programmed with the high-pass filter set to 1 Hz and the low-pass filter set to 90 Hz, the widest range available on the device. From each hemisphere, the neurostimulator stores two channels of differential recordings each made up of one NAc electrode pair (contacts 1 and 2) and another electrode pair in the white matter of the anterior limb of the internal capsule (contacts 3 and 4) to make up a ventral NAc (contacts 1 and 3) and dorsal NAc (contacts 2 and 4) recording. Implantation was performed for subject 1 on 31 January 2020 and for subject 2 on 17 July 2020.

### Recording phase

Immediately after implantation, the subjects entered a 6-month recording phase, during which naturalistic in-lab assessments and ambulatory real-world assessments were performed to identify an electrophysiological biomarker for rDBS in the consecutive stimulation phase. From each hemisphere, activity was recorded from the ventral and dorsal NAc (see Supplementary Information for details).

During the end of the recording phase of the study and after optimization of signal detection, both subjects underwent a day of monopolar survey testing of all the electrode contacts to screen for any acute stimulation effects. One week after this survey testing, each subject was randomized in a single-blind fashion to receive a week of active or sham rDBS unilaterally to assess initial tolerability and optimize stimulation parameters. One subject was randomized to active stimulation and the other sham stimulation. For subject 1, stimulation was administered at 3 mA unilateral for a week, off for a week, 5 mA unilateral for 4 weeks. The subject was unblinded after these 4 weeks and transitioned to open-label stimulation. For subject 2, stimulation was administered at 0.5 mA unilateral for a week, off for a week, 1 mA bilateral (split between two leads) for 4 weeks. The subject randomized to sham stimulation underwent a week of active stimulation testing before transitioning into the open-label phase. Stimulation was well tolerated throughout this protocol with no adverse effects.

#### Behavioral Assessments

Subjects underwent two assessments to evaluate NAc electrophysiology during: (1) anticipation (pre-consumption) of food during standard meals and LOC eating (that is, multi-item buffet assessment, in-lab naturalistic testing); and (2) states of hunger and craving (pre-consumption) (that is, ambulatory assessment, real-world testing). All behavioral assessments were collected during the recording phase (see Fig. 1a).

##### Multi-item buffet

Subjects participated in a modified multi-item buffet designed to provoke LOC eating in a more naturalistic setting (developed by W. Stuart Agras^18^). This buffet of preferred foods was intended to model the at-risk environment of LOC in a controlled setting where real-time brain recordings could be synchronized to video monitoring. For this task, subjects spent the day in this behavioral lab where they were served a breakfast and lunch (~1,000 kcal total). After a mood provocation protocol with an eating disorder psychiatrist (D.S.), the subjects were presented with a 5,000-kcal calorie buffet of preferred foods. The mood provocation was administered based on research showing that negative affective states, compared with neutral mood provocations, are more likely to trigger loss of control/disinhibited eating in patients who binge eat^18^. During the buffet, real-time video with eye tracking (Pupil Lab Pupil Core Eyetracker (https://pupil-labs.com/products/core) and room video) and NAc LFP activity were recorded to capture bites (see Fig. 1c, picture insert^25^). Subjects reported their level of LOC before, during and after the presentation of the foods. The buffet was stopped 15 min after the subject would verbalize that they were experiencing LOC per FDA safety guidelines.

##### Ambulatory assessment

Due to storage capacity limitations, the neurostimulator does not store continuous LFP activity; however, it does record multiple LFP snapshots of activity when an event is detected, based on time of day or when the subject triggers storage (by swiping a magnet over the device). To assess LOC in the real world, subjects were instructed to trigger LFP activity storage (180 s for subject 1, 90 s for subject 2) whenever they had a craving and were about to eat. The storage time was different for each subject based on understanding the subject’s LOC behavior (for example, subject 2’s LOC revolved around multiple snacking events so they would report LOC more frequently) and how many events we could store without losing data. The subjects noted the day and time of the events in a diary and also provided Likert ratings of LOC severity, craving and hunger during these times. In addition to swiping the magnet for cravings, subjects were asked to swipe the magnet when they felt relaxed/‘normal’ to timestamp control periods. Detections were also scheduled to be stored during a randomly selected time of the day during awake (awake detections) and sleep (sleep detections) hours. Both of these events are scheduled LFP recordings that are not user initiated and not tied to an LOC event. As such, low-frequency detections acquired during these scheduled events act as controls to LOC detections and user-initiated events. Our primary analyses were designed to compare NAc LFP activity recorded during reported LOC versus control period magnet swipes. For both subjects, LOC was identified early on to be frequently associated with events of craving in the absence of hunger. In fact, both subjects noted qualitative differences in craving severity with higher severity cravings (referred to as high craving) being associated with LOC-related binges historically. Thus, for our study purposes, the presence of high craving with no hunger (that is, subjects felt at risk of losing control even though they were not hungry) was defined as an at-risk moment, and is referred to as a ‘LOC eating event’. In contrast, events of hunger sensations even in the context of craving (potentially closer to ‘typical eating’ behavior in the presence of metabolic need) were not associated with reported LOC. Subject reporting of LOC using the magnet swipe feature is probably one of the first of its kind, and a very new method for assessing ambulatory, real-world conditions.

### Stimulation phase

After the recording phase, both subjects underwent single-blinded stimulation survey testing in which they received brief bursts of electrical stimulation across all electrode contacts to screen for acute effects. This was followed by a single-blinded, staged, on–off stimulation safety testing period to assess for possible side effects of rDBS. Subjects then entered the 10- to 12-month open-label stimulation phase of the study. In this phase, rDBS was delivered using a bipolar montage of the two ventral contacts on the lead (contacts 1 and 2) on each left and right electrode positioned within the NAc (Fig. 1b). Both subjects received bilateral NAc rDBS via depth electrodes connected to a NeuroPace RNS System to detect and inhibit LOC eating events. Stimulation was delivered at 125 Hz in two 5-s bursts at a charge density of 0.5 μC cm^−2^. Current was incrementally increased over 6 months in both subjects, resulting in a stimulation charge density of 1.5 μC cm^−2^. Detections and stimulations occurred approximately 400 times a day with a stimulation limit set to 700 bouts (or approximately 117 min) per day to limit unnecessary stimulation at night (Supplementary Figs. 4.1 and 4.2). The recording phase was used to identify the biomarker that the rDBS would respond to, whereas the stimulation phase was used to test the safety and side effects of stimulation. Stimulation parameters were derived from those used in epilepsy and thought to have a disruptive effect on the target neural activation^26–28^. Stimulation was started at a low amplitude using contacts that a monopolar assessment previously revealed to elevate mood to avoid well-described, DBS-induced side effects to this region such as hypomania. Stimulation was started unilaterally per FDA guidance and added stimulation to the contralateral hemisphere, and then slowly titrated every 3 months until an improvement in LOC was detected. Frequency and pulse width were held constant such that only changes in amperage were made.

Based on the recording phase, each subject’s device was programmed to detect brief increases in low-frequency activity in both the left and the right ventral NAc (Supplementary Information). These detections of low-frequency activity triggered bilateral NAc rDBS (~1 μC cm^−2^ charge density, 10-s duration). Low-frequency triggered bilateral stimulation has been well tolerated by both subjects. Neither subject 1 nor subject 2 experienced a serious adverse event and all reported events were self-limited (Supplementary Table 1). Examination of sensitivity and specificity can be found in Supplementary Figs. 1 and 2.

High-frequency DBS has been widely used to effectively treat symptoms in psychiatry (for review see Sullivan et al.^29^). High-frequency stimulation has been found to both excite and suppress neural activation in different ways, and therefore it has been difficult to pinpoint the direct mechanism of action. However, although there are a number of theories surrounding the mechanism of action of high-frequency DBS, only one common mechanism has been found to explain the various neural effects found from high-frequency stimulation: DBS dissociates input and output signals in the stimulated nucleus and disrupts abnormal information flow in pathological conditions (aligning with the ‘disruption hypothesis’ of stimulation)^26^. Furthermore, therapeutic effect has been linked to effective suppression of low-frequency oscillations, which was accomplished only by high-frequency stimulation^17,30^.

### Data collection and analyses

#### LFP analyses

All LFP data analyses were performed in R2014b (Mathworks Inc.) using customized scripts built on the FieldTrip Toolbox^31^ (Donders Institute for Brain, Cognition and Behaviour, Radboud University, the Netherlands; see http://fieldtriptoolbox.org).

Electrophysiological data were recorded at 250 Hz and bipolar re-referenced online. The recorded LFP data were cleaned offline, using the following steps: (1) de-trending raw LFP data; (2) 60-Hz (and harmonics: 120-Hz, 180-Hz) notch filters; and (3) 1- to 90-Hz bandpass filter. The cleaned data were then epoched with respect to the trial onset or, in the case of ambulatory data, centered at the time of the magnet swipe. LFP data recorded during stimulation trials were not analyzed due to the high-frequency nature of the stimulation and associated artifact in the recording.

All spectral analyses were computed at each channel. Data underwent morlet wavelet time frequency transformation and then the power was averaged within the canonical frequencies: delta (2–4 Hz), theta (4–8 Hz), alpha (8–12 Hz), beta (13–30 Hz), gamma (31–50 Hz) and high-gamma (50–90 Hz) frequency bands. In some cases, we analyzed low-frequency power combining delta–theta (2–8 Hz). Average estimates of bandpower were derived by taking the mean power across the frequencies within each predefined canonical frequency band with the s.e.m. reported. Ambulatory: data epoch was extracted in the pre-magnet swipe time (1–2 min) before undergoing above spectral analysis. Multi-item buffet: data epochs were extracted 2 s before the time-locked bite within each task condition—standard eating and LOC eating. Bites were defined as the moment food entered the subject’s mouth, as defined by visual inspection of synchronized eye tracking (Pupil Lab Pupil Core Eye-tracker (https://pupil-labs.com/products/core)) and video captured during the task. The 2-s windows were chosen based on our previous pre-clinical mouse work looking at the same window with mice approaching the high-fat food^5^ and examining time-locked eye tracking and video per bite examining time lingering on food in hand/spoon or food coming to mouth. LFP data were captured using the neurostimulator’s magnet feature for storing which was wireless controlled by the research team in an adjacent room with a field of view on the subject behind a one-way mirror window.

#### Statistical analyses

Statistical analysis (Minitab Data and Statistical Toolbox) was performed using two-sided Student’s *t*-test to compare bandpower means during clinical tasks. One-way analysis of variance (ANOVA) followed by Student’s *t-*test was performed comparing mean power during ambulatory recordings under three conditions (control, craving, hunger). The *χ*^2^ test was performed on the rDBS detection count for each subject. A statistical significance criterion of *α* = 0.05 was used for all tests. Results are shown as a mean ± s.e.m. unless otherwise noted.

#### Signal detection

For each subject, we programmed the device to detect brief increases in low-frequency activity in both the left and right ventral NAc. To help differentiate the brief bursts of low-frequency activity associated with LOC from the longer periods of delta activity observed during sleep, we selected the RNS System ‘Area’ detector. The area under the curve (AUC) detector takes a signal AUC measurement every 2 s (short-term trend) and compares it with an average of AUC measurements from the past 2 min (long-term trend). If the current (short-term) AUC measurement exceeds the average long-term trend measurements of AUC, by the programmed threshold (in our cases by 63–100%), a detection is made. Stimulation detection threshold was determined by the analyses of the abnormal NAc activity. As the low-frequency activity observed during sleep increases the longer-term AUC, it is difficult for the short-term AUC measurement to exceed the longer-term AUC value by the specified threshold, thereby reducing the amount of activity detected during sleep. As we observed increases in low-frequency power during real-world LOC in both the left and the right ventral NAc, we also required the activity to be present simultaneously in both the left and the right NAc for the detection to trigger rDBS delivery.

### Clinical measures

The primary study endpoint is at least 50% of subjects exhibiting a decrease in the number of LOC eating events per week, utilizing the Ecological Momentary Assessment (EMA). Reported below are preliminary EMA data from subjects 1 and 2, as well as additional support from the monthly surveys and ELOCS:

1. EMA: 1 week out of every month, participants receive a survey at two semi-random times per day (morning and evening) that asks about eating behaviors, including frequency and severity of loss of control. Supplementary Figs. 3.1 and 3.3 display the number of LOC eating events per 28 d, which is the endpoint for the study (any reduction in LOC from baseline), for subjects 1 and 2, respectively.
2. ELOCS: a retrospective survey assessed once a month targeting LOC frequency and severity. Supplementary Figs. 3.2 and 3.4 for subjects 1 and 2 display the LOC frequency score and LOC frequency severity based on this 18-item assessment.

### Reporting summary

Further information on research design is available in the Nature Research Reporting Summary linked to this article.

## Online content

Any methods, additional references, Nature Research reporting summaries, extended data, supplementary information, acknowledgements, peer review information; details of author contributions and competing interests; and statements of data and code availability are available at 10.1038/s41591-022-01941-w.

## Supplementary information

Supplementary Information Supplementary Methods, Outcomes, Figs. 1–4, Tables 1 and 2, and Protocol.

Reporting Summary

## Data Availability

The data that support the findings of the present study are available upon reasonable request to the corresponding author (C.H.H.). The data are not publicly available yet, due to them containing information that could compromise research participant privacy/consent. As this is part of an ongoing clinical trial, enrolling additional subjects (trial registration no. NCT03868670), all data will be deposited into the Data Archive Brain Initiative (https://dabi.loni.usc.edu) as part of the BRAIN Initiative on completion of the study. During this time, any request will be reviewed in a timely manner by the corresponding author, corresponding author’s institution and ultimately shared within reason of a signed data transfer agreement.

## Extended data

Extended data are available for this paper at 10.1038/s41591-022-01941-w.

## Code availability

All code has been made publicly available and can be found at https://github.com/rajatss123/LOC_NM_Code.
