# Supplementary material for: Pilot study of responsive nucleus accumbens deep brain stimulation for loss-of-control eating
Source: Nat Med. 2022 Aug 29;28(9):1791–6. doi: 10.1038/s41591-022-01941-w (PMC9499853; doi:10.1038/s41591-022-01941-w)
Supplement: Supplementary file 1 — Supplementary Methods, Outcomes, Figs. 1–4, Tables 1 and 2, and Protocol. [file 41591_2022_1941_MOESM1_ESM.pdf]

nature portfolio

https://doi.org/10.1038/s41591-022-01941-w

# Supplementary information

# Pilot study of responsive nucleus accumbens deep brain stimulation for loss-of-control eating

In the format provided by the authors and unedited

# Appendix: Supplemental Information

## Table of Contents

Sensitivity and Specificity of Signal Detection ........ 1
Supplemental Stimulation Outcomes ........ 4
Supplemental Figures and Tables ........ 5
References ........ 12
Clinical Trial Protocol ........ 13

## Sensitivity and Specificity of Signal Detection

We investigated the sensitivity and specificity of our low-frequency detector by reviewing stored LFP recordings collected by the RNS System (**Figure S2A**). The NeuroPace RNS System has a limited storage capacity (12.5 minutes of 4 channel recordings), and this storage bandwidth can only be cleared by a user-initiated transfer of the data off the neurostimulator, triggered by an external remote monitor. For the sensitivity/specificity analysis, we visually reviewed (**Figure S1**): 1-LFP recordings for LOC reported events that also had a detection (True Positive, TP); 2-LOC reported events that did not have a detection (False Negative, FN); 3-awake LFP recordings where detections were observed (False Positive, FP); 4-awake LFP recordings where no detections were observed (True Negative, TN). All specificity, sensitivity and accuracy analyses excluded detections during sleep.

$$\text{Sensitivity} = TP/(TP + FN)$$

$$\text{Specificity} = TN/(TN + FP)$$

$$\text{Accuracy} = (TP+TN) / (TP+TN+FN+FP)$$

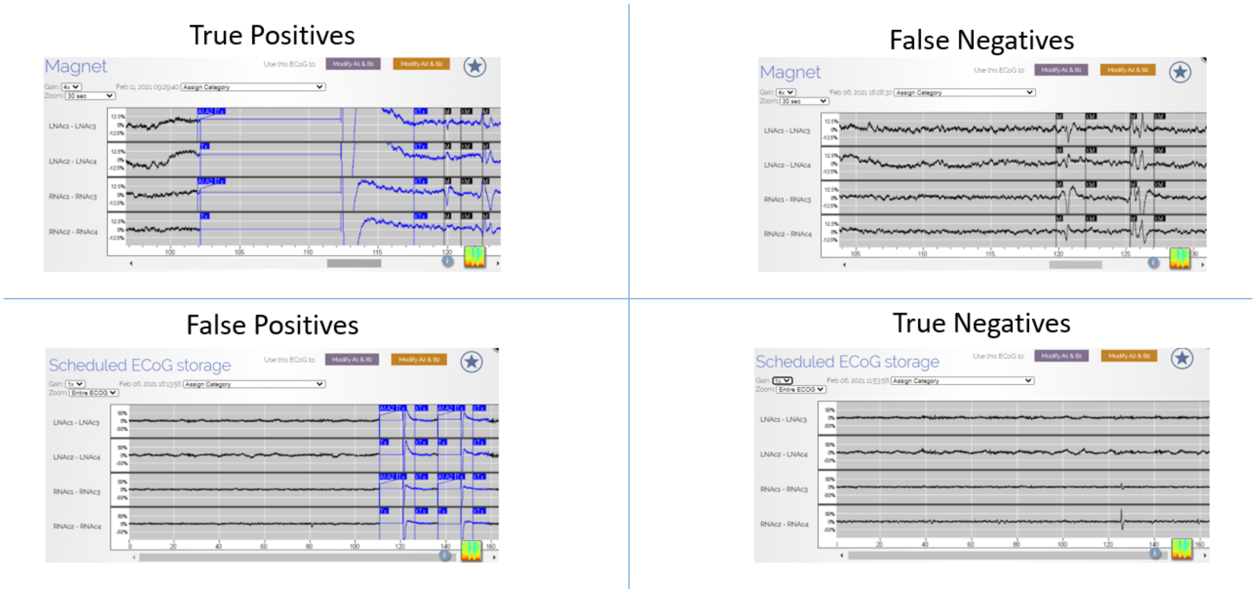


***Figure S1*** *– Example electrographic recordings showing how sensitivity and specificity of our detector was calculated.*

To examine the sensitivity and specificity of low-frequency power fluctuations to LOC eating behavior, we first visually reviewed LFP recordings for LOC reported events that also had a detection (True Positive), LOC reported events that did not have a detection (False Negative), awake LFP recordings where detections were observed (False Positive), and awake LFP recordings where no detections were observed (True Negative). Analysis of the sensitivity and specificity of the detector revealed Subject 1's sensitivity of 82.4% (71.2-90.5% for 95% Confidence Interval) and specificity of 48.3% (42.6-54.1%, 95% CI). For Subject 2, the programmed detector had a sensitivity of 67.8% (54.4-79.4% for 95% CI) and specificity of 58.8% (54.1-63.3%, 95% CI). The accuracy of LOC detection using these more longitudinal recordings of low-frequency was 54.6% for Subject 1 and 59.7% for Subject 2. In general, for both subjects, the sensitivity of detection was high, however, the specificity was lower (Figure S3). This indicates that the neurostimulator detected low-frequency activity associated with the majority of reported LOC events, but that the device also detected increased low-frequency activity during awake LFP recordings. As there were no clear side effects of NAc stimulation, and stimulation was not perceivable by the subjects at any point, the lower specificity for this proof-of-concept study was not deemed a risk factor.

A critical limitation of the above analysis is that it only includes the subset of detections successfully stored. LFP time-locked to user-initiated or scheduled events were always recorded. However, depending on the neurostimulator storage bandwidth, LFP data time-locked to detections may not be stored. Therefore, while all detection timestamps were stored for offline analyses, only a subset of detection-locked LFP data could be saved due to the limited storage capacity of the NeuroPace RNS System (12.5 minutes of 4 channel recordings) prior to subject-initiated transfer of data. As such, LFP analyses during detections is limited to the subgroup successfully stored. Since this is a limitation to the reported sensitivity/specificity analysis, we also compared the number of detections in the minutes to hours around a self-reported LOC versus non-LOC (scheduled, normal, etc) reported events for each subject (**Figure S2B**). All detections were timestamped and recorded for offline analyses, but the LFP data time-locked to each detection may not have been. Therefore, by quantifying the percentage of detections surrounding LOC eating events vs non-LOC events, we can more comprehensively examine specificity. In both subjects, we observed a significant increase in the number of detections in the hour and minutes before and after LOC eating event was reported compared (determined by magnet swipe t = 0) to non-LOC detections (**Figure S2B**, Subject 1: 1) LOC event detections (*60-30min prior to reporting LOC*: 60.75±23.18 detections, *30-60min after reporting LOC:* 60.13±26.81 detections*)* vs. non-LOC detection per 30min window (8.75±1.11 detections), Student's t-test comparing mean detections, *p<0.05; 2) LOC event detections (*30-15min prior to reporting LOC*: 31.25±12.10 detections, *15-0min prior to reporting LOC:* 32.63±12.5 detections, *0-15min after reporting LOC:* 29.25±13.94 detections, *15-30min after reporting LOC:* 29±15.57 detections *)* vs. non-LOC detection per 15min window (4.38±0.56 detections), Student's t-test comparing mean detections, *p<0.05. Subject 2: 1) LOC event detections (*60-30min prior to reporting LOC*: 24±11. detections, *30-60min after reporting LOC:* 23.5±14.41 detections*)* vs. non-LOC detection per 30min window (8.32±3.41 detections), Student's t-test comparing mean detections, *p<0.05; 2) LOC event detections (*30-15min prior to reporting LOC*: 14.33±9.14 detections, *15-0min prior to reporting LOC:* 15.83±4.22 detections, *0-15min after reporting LOC:* 7.5±3.30 detections, *15-30min after reporting LOC:* 9.33±4.11 detections) vs. non-LOC detection per 15min window (4.16±1.71 detections), Student's t-test comparing mean detections, *p<0.05).

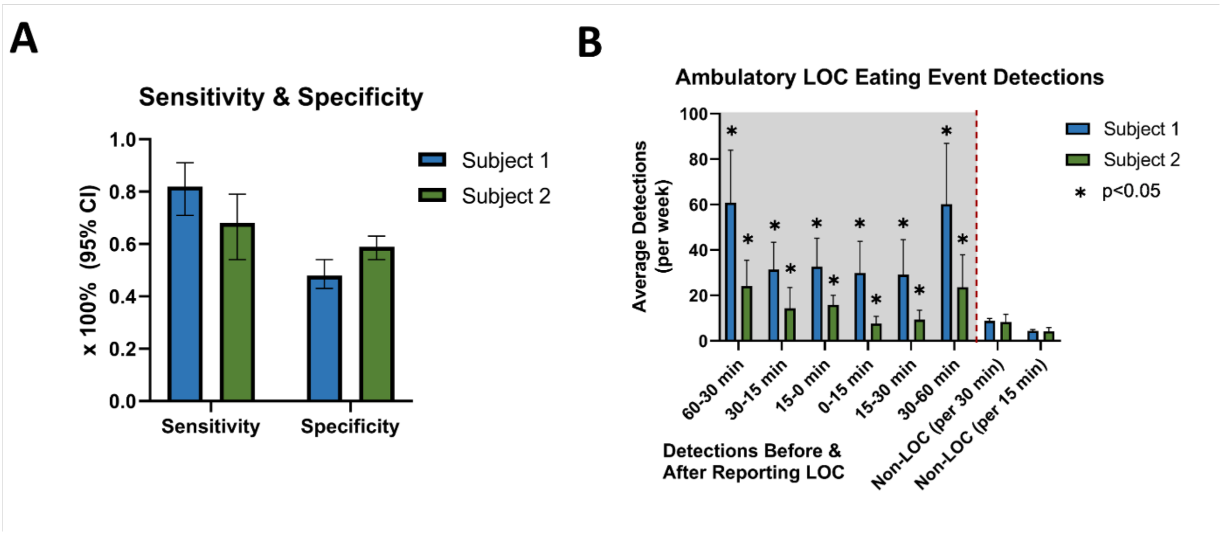


***Figure S2*** *– Sensitivity/Specificity Analyses*. A) Analysis of the sensitivity and specificity of the detector revealed Subject 1's sensitivity of 82.4% (71.2-90.5% for 95% Confidence Interval) and specificity of 48.3% (42.6-54.1%, 95% CI). For Subject 2, the programmed detector had a sensitivity of 67.8% (54.4-79.4% for 95% CI) and specificity of 58.8% (54.1-63.3%, 95% CI). Error bars represent upper and lower limit for 95% CI. B) In addition, we analyzed the number of low-frequency detections throughout the day and observed an increase in the number of detections in the hour, and minutes before and after a reported LOC eating event (*LOC events:* Subject 1, N = 90; Subject 2, N= 39; *Non-LOC event:* Subject 1, N = 133, Subject 2, N = 87; Student's t-test comparing mean detection, *p<0.05). Magnet swipe for reporting LOC eating event defined as t = 0. Bar graphs represents mean detection ± S.E.

## Supplemental Stimulation Outcomes

To identify whether there were changes in mood and impulsivity following 6 months of stimulation, we analyzed our baseline and 6-month visit mood surveys (Positive Negative Affect Schedule; PANAS) and Impulsivity questions (Ecological Momentary Assessment of Impulsivity[1]). We found that after 6 months of responsive DBS, there were no self-reported effects on positive affect or impulsivity as assessed by PANAS scale and Impulsivity measures (p>.22) for either subject. We did find that subject 1 showed a significant (p<.001) decrease in PANAS negative affect from baseline to 6-months post-stimulation.

## Supplemental Figures and Tables

### *Figure S3.1* - Ecological Momentary Assessment Ratings – Subject 1

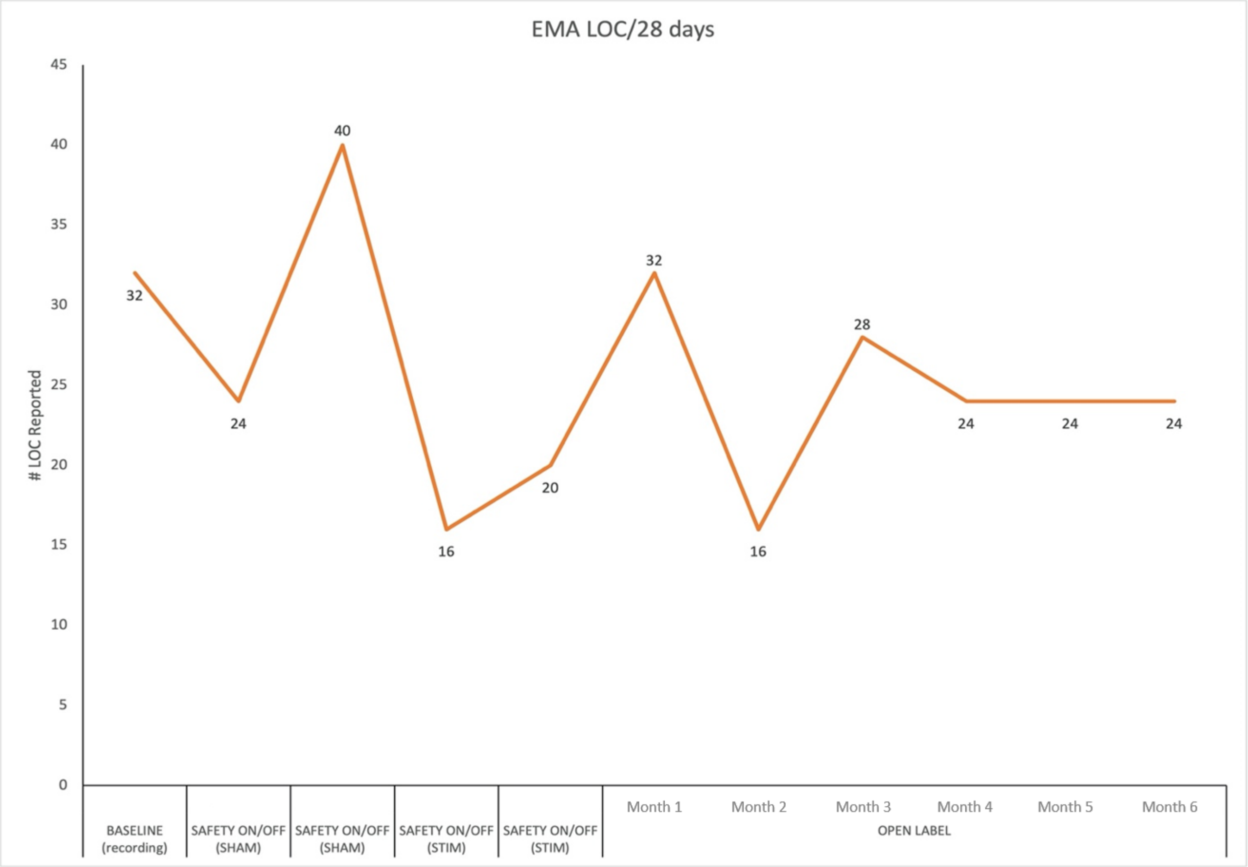


***Figure S3.2*** **- Eating Loss of Control Scale – Subject 1**

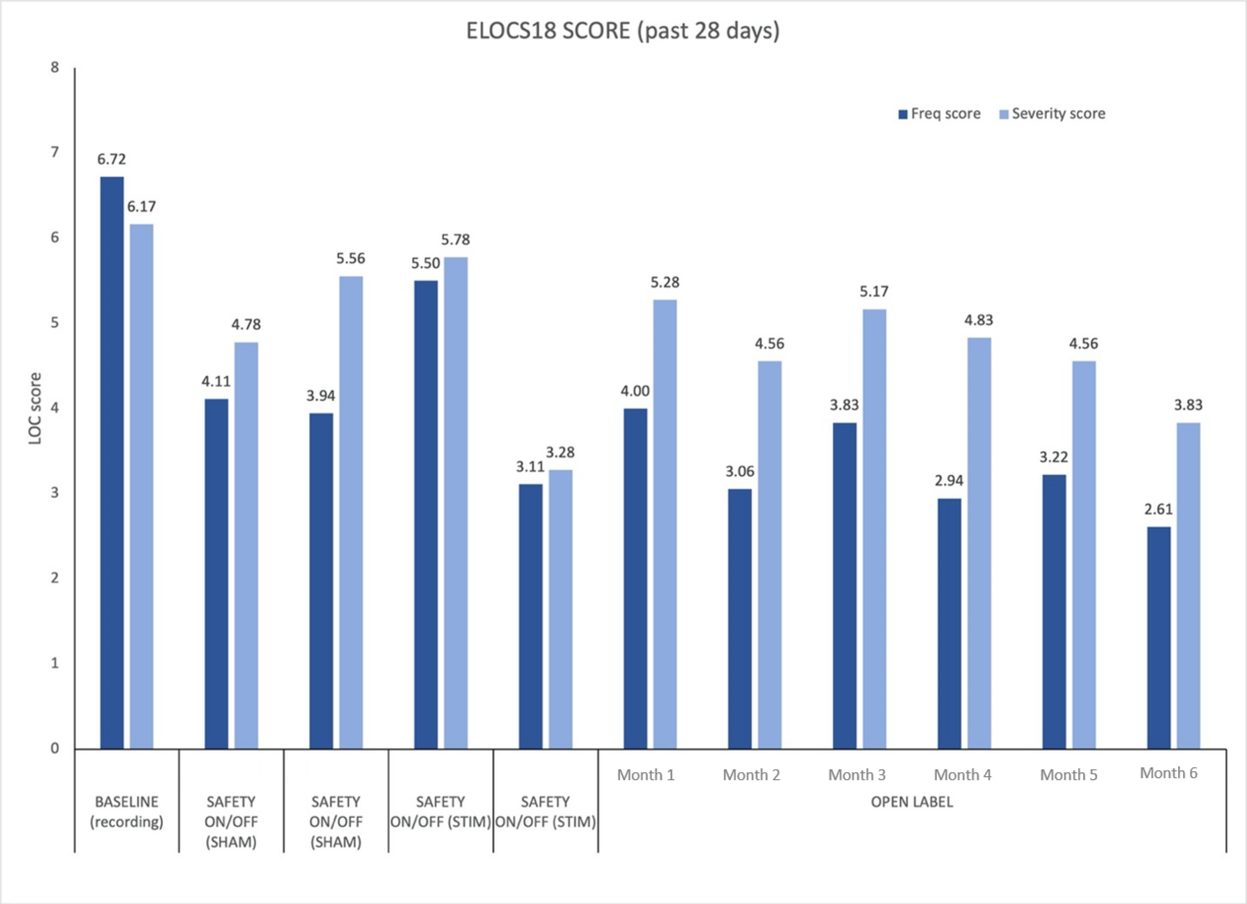


***Figure S3.3*** **- Ecological Momentary Assessment Ratings – Subject 2**

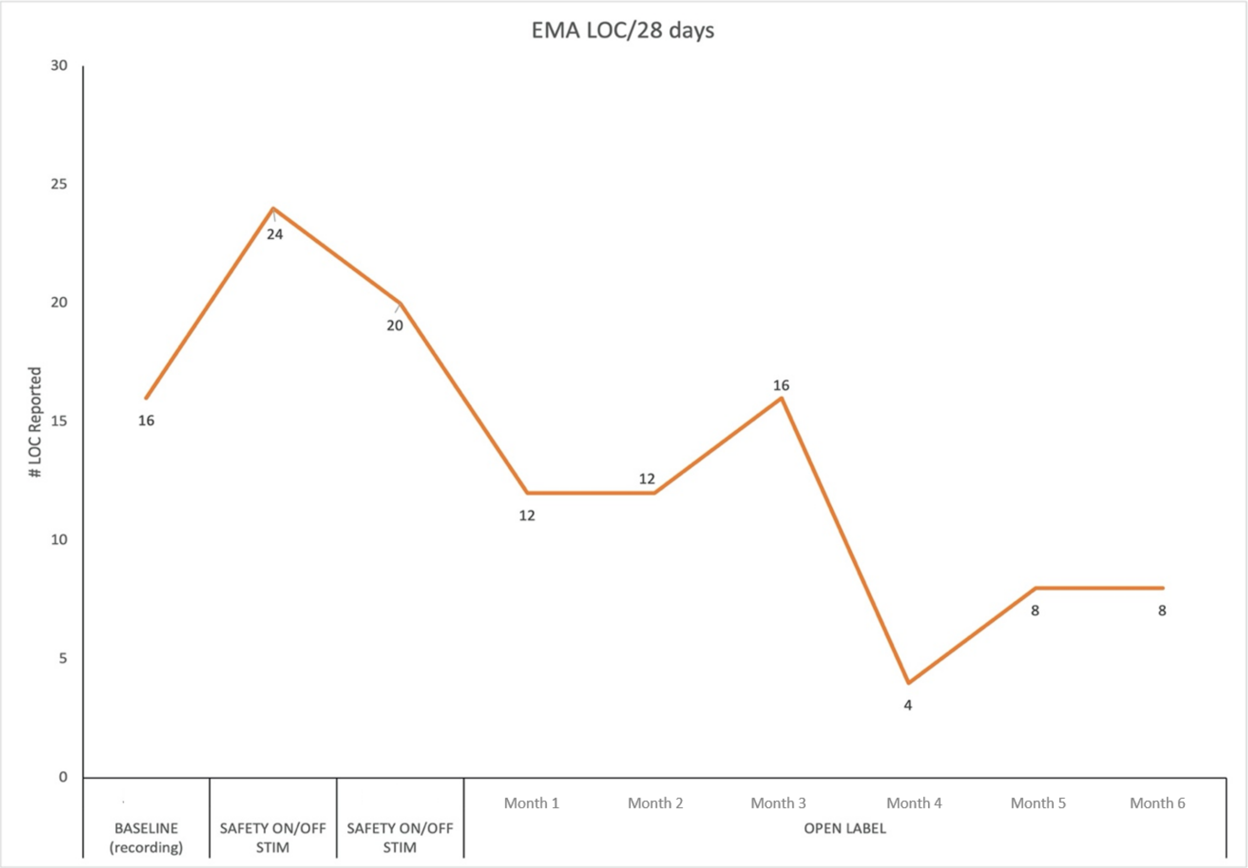


***Figure S3.4*** **- Eating Loss of Control Scale – Subject 2**

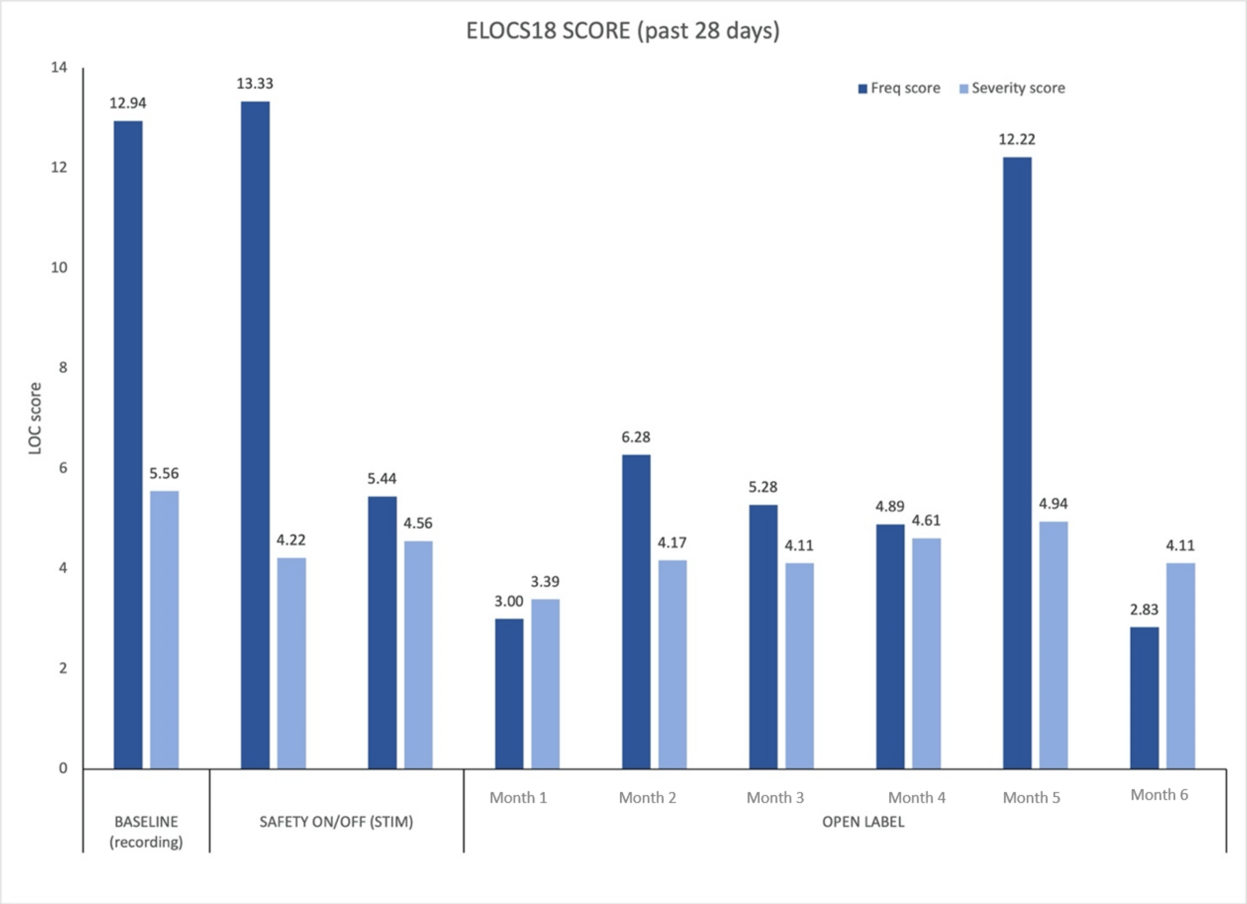


*Figure S3.5* **- Weight by Month during Open Label Stimulation Phase**

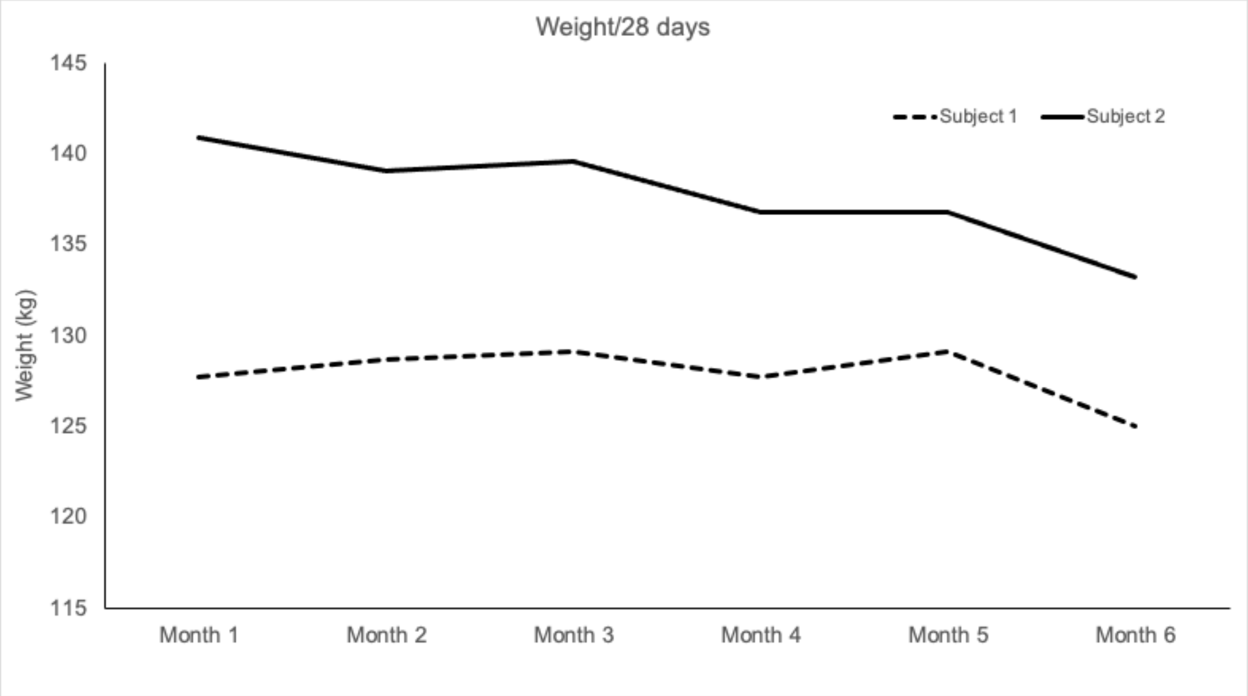

*Figure S3.6* - **BMI by Month during Open Label Stimulation Phase**

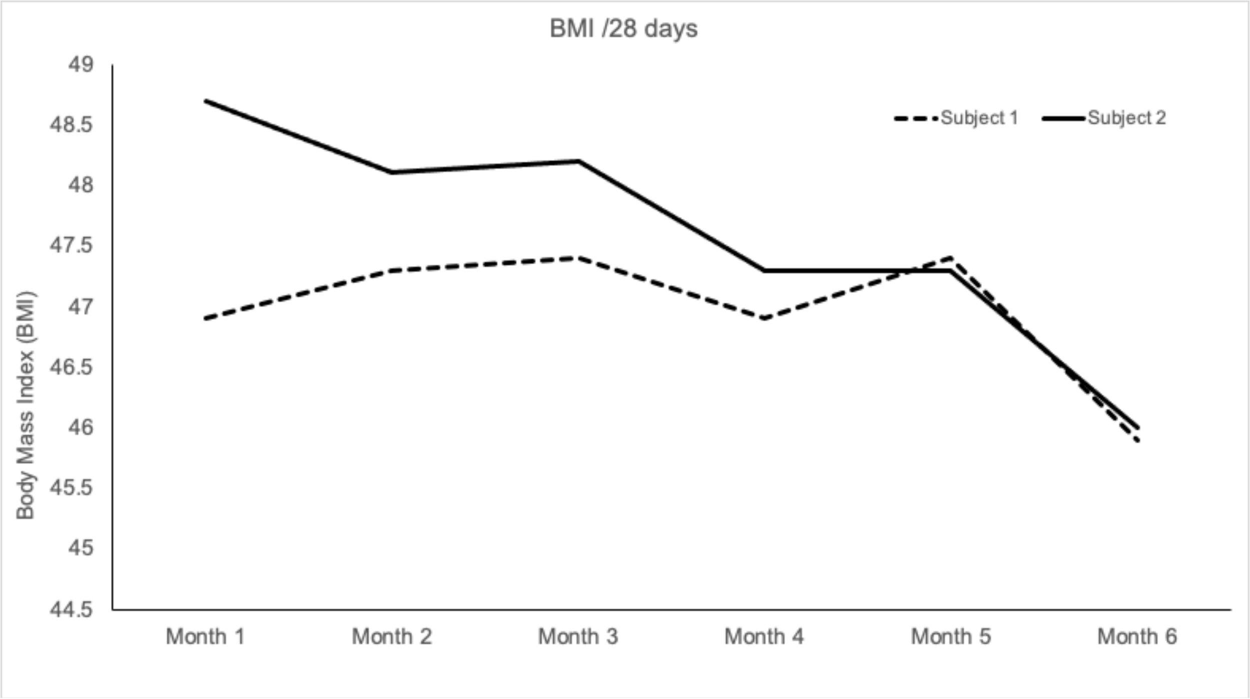


*Figure S4.1* – **Number of Detections per month (6 month snapshot) – Subject 1**

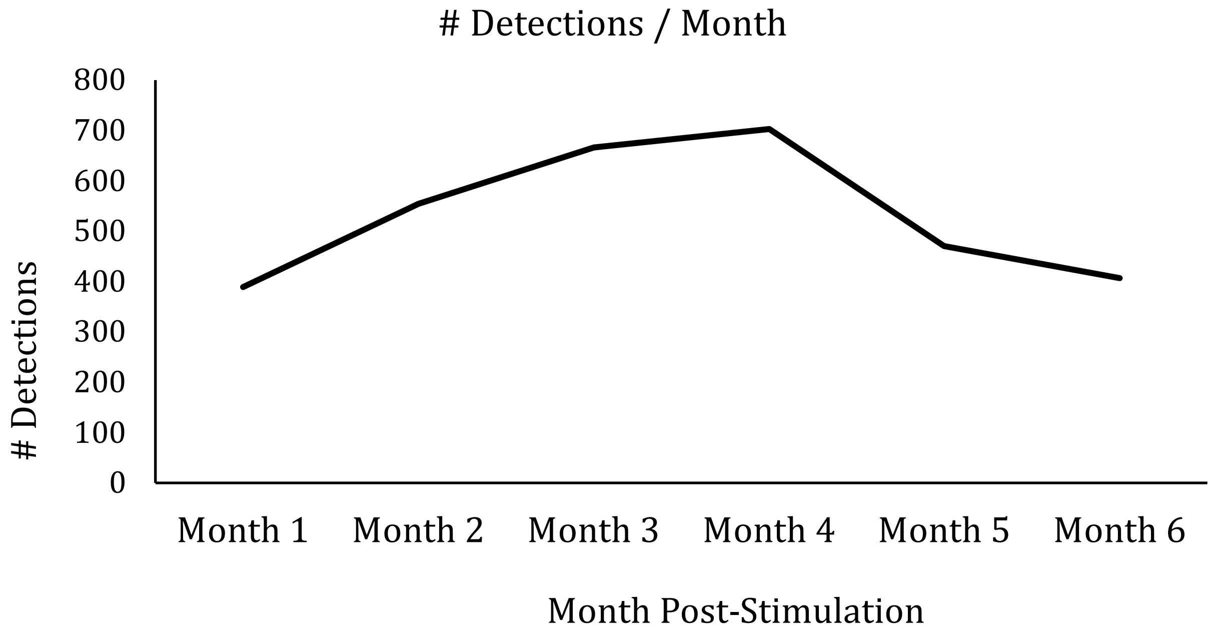

*Figure S4.2* – **Number of Detections per month (6 month snapshot) – Subject 2**

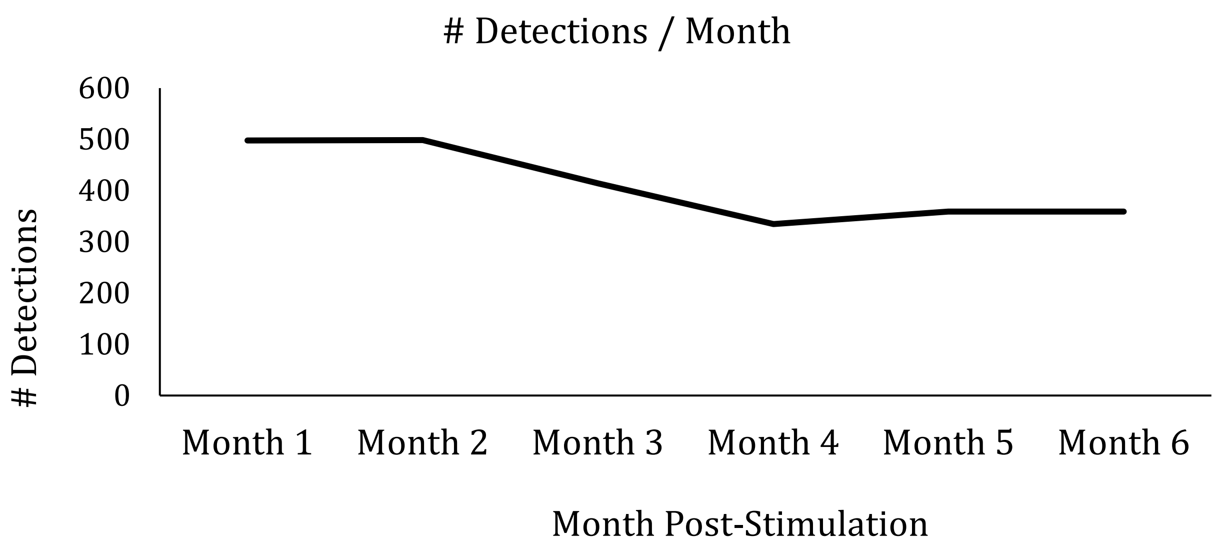


***Table S1*- Stimulation-Related Adverse Events**

| Subject # | Days on Intervention | Adverse Event | Relationship to Intervention* | Severity | Serious (Y/N) | Outcome** |
|---|---|---|---|---|---|---|
| 1 | 0 | Headache | Definite (surgery) | Moderate | N | RWT |
| 1 | 0 | Incision pain | Definite (surgery) | Moderate | N | RWT |
| 1 | 0 | Edema | Definite (surgery) | Mild | N | RWT |
| 1 | 0 | Anxiety | Possible (surgery) | Moderate | N | RWOT |
| 1 | 8 | Sadness | Not related | Mild | N | RWOT |
| 1 | 20 | Pain behind ear/jaw | Not related | Moderate | N | RWT |
| 1 | 27 | Zoning out | Not related | Mild | N | RWOT |
| 1 | 30 | Insomnia | Possible | Mild | N | RWOT |
| 1 | 102 | "Fluttering"/anxiety | Not related | Mild | N | RWOT |
| 2 | 0 | Headache | Definite (surgery) | Moderate | N | RWT |

| | | | | | | |
|---|---|---|---|---|---|---|
| 2 | 0 | Incision pain | Definite (surgery) | Mild | N | RWT |
| 2 | 0 | Edema | Definite (surgery) | Moderate | N | RWT |
| 2 | 1 | Lightheade d | Possible | Mild | N | RWOT |
| 2 | 77 | Swelling legs/feet | Not related | Mild | N | RWT |
| 2 | 84 | Insomnia | Not related | Mild | N | RWOT |

* *Definite, Possible, Not Related*

** *Outcome: Recovered, without treatment (RWOT); Recovered, with treatment (RWT)*

***Table S2*- Contact location coordinates**

| | | | | | |
|---|---|---|---|---|---|
| **Subject 1** | | | | | |
| AC-PC distance (mm) | 23.49 | | | | |
| Points of interst | Lateral (distance to midline) | A-P (anterior to AC) | Vert (from above AC-PC plane) | Angle from mid-Sagittal | Angle from Axial |
| Left_Target (0.0mm) | -6.47 | 3.46 | -6.27 | 41.5 | 58.9 |
| Left_C1 | -7.58 | 4.22 | -5 | 41.5 | 58.9 |
| Left_C2 | -9.82 | 5.75 | -2.48 | 41.5 | 58.9 |
| Left_C3 | -11.81 | 7.11 | -0.23 | 41.5 | 58.9 |
| Left_C4 | -13.62 | 8.34 | 1.82 | 41.5 | 58.9 |
| Right_Target (0.0mm) | 3.11 | 4.54 | -4.41 | 40 | 88.7 |
| Right_C1 | 4.27 | 4.57 | -3.03 | 40 | 88.7 |
| Right_C2 | 6.64 | 4.63 | -0.2 | 40 | 88.7 |
| Right_C3 | 8.57 | 4.68 | 2.1 | 40 | 88.7 |
| Right_C4 | 11.14 | 4.75 | 5.16 | 40 | 88.7 |
| **Subject 2** | | | | | |
| AC-PC distance (mm) | 24.67 | | | | |
| Points of interst | Lateral (distance to midline) | A-P (anterior to AC) | Vert (from above AC-PC plane) | Angle from mid-Sagittal | Angle from Axial |
| Left_Target (0.0mm) | -2.56 | 5.1 | -4.77 | 37.3 | 73 |
| Left_C1 | -3.46 | 5.46 | -3.59 | 37.3 | 73 |
| Left_C2 | -5.81 | 6.41 | -0.5 | 37.3 | 73 |
| Left_C3 | -7.58 | 7.11 | 1.81 | 37.3 | 73 |
| Left_C4 | -9.64 | 7.94 | 4.52 | 37.3 | 73 |
| Right_Target (0.0mm) | 5.03 | 5.72 | -4.58 | 36.3 | 72.5 |
| Right_C1 | 5.9 | 6.09 | -3.39 | 36.3 | 72.5 |
| Right_C2 | 8.2 | 7.08 | -0.27 | 36.3 | 72.5 |
| Right_C3 | 9.92 | 7.82 | 2.07 | 36.3 | 72.5 |
| Right_C4 | 11.93 | 8.68 | 4.8 | 36.3 | 72.5 |

*Contact (C) coordinates reflect center of electrode contact

## References.

1. Tomko RL, Solhan MB, Carpenter RW, et al. Measuring impulsivity in daily life: the momentary impulsivity scale. Psychol Assess 2014;26:339-49.

# PROTOCOL

**TITLE:** **RESPONSIVE NEUROSTIMULATION FOR LOSS OF CONTROL OVER EATING**

**PROTOCOL NUMBER:**

**VERSION NUMBER:** 1.1

**DATE:** 30 October 2018

**IDE NUMBER:** Q171158

**TEST PRODUCT:** NeuroPace RNS® System

**INVESTIGATOR:** Dr. Casey Halpern, M.D

TABLE OF CONTENTS

1. BACKGROUND

1.1 Treatment refractory obesity

1.2 Rationale for NAc-RNS

1.3 Loss of control over eating in treatment-refractory obesity: Target patient population

1.4 Role of NAc in gating loss of control over eating

1.5 Pre-clinical Data

1.5.1 Identifying a predictive biomarker

1.5.2 Pre-clinical studies of NAc-RNS in mice

1.6 Preliminary findings in humans

2. DEVICE DESCRIPTION

2.1 RNS® System Product Description

2.2 System Overview

3. OBJECTVES AND ENDPOINTS

3.1 Study Objectives

3.2 Study Endpoint

4. STUDY DESIGN

4.1 Overview and Design

4.2 Subject Selection

4.2.1 Inclusion Criteria

4.2.2 Exclusion Criteria

4.3.0 Study Assessments

4.3.1 Pre-screen and Informed Consent

4.3.2 Pre-operative Baseline

4.3.3 Surgical Procedure

4.3.4 Monopolar Assessment and Randomization

4.3.5 Recording Phase

4.3.6 Follow-up Assessment

4.3.6 Stimulation Phase

4.4 Staggered Enrollment

**5. ASSESSMENT OF SAFETY**
5.1. Identification of Risks
5.1.1 Mitigation of Risks
5.1.2 Potential Benefits and Alternatives
5.2 Safety Parameters and Definitions
5.2.1 Adverse Events
5.2.1.1 Assessment of Severity of Adverse Event
5.2.1.2 Assessment of Causality of Adverse Event
5.2.1.3 Abnormal Lab Values
5.2.1.4 Abnormal Vital Sign Values
5.2.2 Serious Adverse Events
5.2.3 Unanticipated Adverse Device Effect (Immediately Reportable)
5.3 Monitoring and Oversight
5.3.1 Safety Monitoring Committee
5.3.2 Patient, Treatment, and Study Discontinuation
5.3.3 Psychiatric Conditions
5.4 Protocol Deviations
**6. STATISTICAL CONSIDERATIONS AND ANALYSIS PLAN**
6.1 Selection of Sample Size
6.2 Data Analysis
**7. DATA COLLECTION AND MANAGEMENT**
7.1 Data Quality Assurance
7.2 Electronic Case Report Forms
7.3 Source Data Documentation
7.4 Confidentiality of Data
7.5 Resource and Data Sharing Policy
7.6 Compliance with Laws and Regulations
7.7 Conflict of Interest Policy
7. 8 Informed Consent Process
7.9 Retention of Records
**8. REFERENCES**

**9. LIST OF TABLES AND FIGURES**

9.1 Mouse Model NAc Stimulation

9.2 Mouse Model Predictive Biomarker

9.3 Result Summary of Different Electrical Stimulation Protocols

9.4 Human Subject Preliminary Electrographic Findings

9.5 The RNS System and Components

9.6 RNS System Overview

9.7 Schedule of Assessments

9.8 Adverse Event Severity Rating Scale

**10. APPENDIX A: SUMMARY OF FUTILITY PHASE**

10.1 Extended Recording

10.2 Novel Software Testing

10.3 Self-directed Stimulation

10.4 Scheduled Stimulation

10.5 Futility

**List of Abbreviations**

| | |
|---|---|
| AE | Adverse Event |
| BDI-II | Beck Depression Inventory-2 |
| BMI | Body Mass Index |
| CFR | Code of Federal Regulations |
| C-SSRS | Columbia Suicide Severity Rating Scale |
| CT | Computerized Tomography |
| DEBQ | Dutch Eating Behavior Questionnaire |
| DSMC | Date Safety Monitoring Committee |
| eCFR | Electronic Case Report Form |
| EDC | Electronic Data Capture |
| EDE | Eating Disorder Examination |
| ELOCS | Eating Loss of Control Scale |
| EMA | Ecological Momentary Assessment |
| FDA | Federal Drug Administration |
| GCP | Good Clinical Practice |
| HF | High Fat |
| HIPAA | Health Insurance Portability and Accountability Act |
| HRPP | Human Research Protection Program |
| ICF | Informed Consent Form |
| ICH | International Conference on Harmonisation |
| IRB | Institutional Review Board |
| LFP | Local Field Potential |
| LOC | Loss of control |
| MacCAT-CR | MacArthur Competence Assessment Tool |
| MID | Monetary Incentive Delay |
| MOP | Manual of Procedures |
| MRI | Magnetic Resonance Imaging |
| NAc-RNS | Nucleus accumbens responsive neurostimulation |
| PANAS | Positive and Negative Affect Scale |
| PDMS | Patient Data Management System |
| SAE | Severe Adverse Event |
| SCID | Structured Clinical Interview |
| SMC | Safety Monitoring Committee |
| UADE | Unanticipated Adverse Device Effect |

## 1. BACKGROUND:

This is a single site, feasibility study to support development of a novel therapeutic approach for utilizing the NeuroPace RNS® System for nucleus accumbens responsive neurostimulation (NAc-RNS) to ameliorate loss of control over (LOC) eating in persons with treatment-refractory obesity, specifically those who have failed gastric bypass surgery. NAc-RNS targets well-defined neuronal populations within the human ventral striatum that have known anatomical and physiological relationships with food reward processing that provide both a key role in homeostatic regulation during meals and a particular vulnerability to dysfunction in the setting of the obese state (Volkow et al, 2008). This study leverages an already commercially available device, and development and regulatory work already completed or committed to by NeuroPace, and builds upon preclinical studies that have been performed utilizing an off-the-shelf, commercially available system adapted to mice. The pre-clinical studies identified NAc-RNS as a promising intervention for LOC eating that exhibited behavioral specificity and no detected adverse effects. In addition to clear translational value, these studies have revealed that stimulating this brain region involves rescuing receptor cell-types already thought to be impaired by the obese state (i.e. D2-receptor expressing medium spiny neurons) (Halpern et al, 2013; Wang et al, 2014).

### 1.1 Treatment-refractory Obesity

Over the past several decades obesity has increased in prevalence worldwide to epidemic proportions. In the U.S. alone, 80 million adults are categorized as obese (BMI >30 kg/m) (Ogden et al, 2012). Along with this alarming rise in obesity, the U.S. has experienced a concomitant rise in obesity-related comorbidities, including hypertension, dyslipidemia, type-2 diabetes, atherosclerotic heart disease, obstructive sleep apnea, and several malignancies (Guh et al, 2009), all adversely affecting quality of life and decreasing overall lifespan (Fontaine and Barofsky, 2001). Thus, obesity is a major public health challenge costing the U.S. $150 billion annually with obese patients experiencing 46% higher inpatient costs, 27% more physician visits and outpatient costs, and 80% higher spending on prescription drugs than normal-weight individuals (Finkelstein et al, 2009). Despite improved preventative care and treatment efforts, the obesity epidemic continues, suggesting the need for novel therapeutics.

Of the current obesity prevalence, 30 million obese adults should at least be considered for bariatric surgery, though only about 250,000 bariatric surgeries are currently performed per year (Flegal et al, 2016; Jensen et al, 2014). LOC eating appears to be related to failure of bariatric surgery in at least 50% of cases, that is, about 125,000 cases per year. Moreover, LOC eating is seen in all obese binge

eaters (and non-obese binge eaters), with estimates of 50% of all obese individuals suffering from LOC eating. This LOC over food has been reported to be associated with significantly lower health-related quality of life and more emotional and stress eating (Engstrom et al, 2015).

### 1.2 Rationale for NAc-RNS

This intervention is intended to ameliorate this unmet need of many adult obese Americans with intractable LOC eating. Not surprisingly, cognitive-behavioral therapy and weight-control medications have had moderate success at best in controlling LOC eating which often manifests as binge eating (Bray and Tartaglia, 2000; Grilo et al, 2011; McElroy et al, 2015). Bariatric surgery is the most effective treatment for refractory morbid obesity, but does not work for all patients; in fact, several Stanford investigators have examined patient characteristics that predict unsuccessful postsurgical outcomes (Robinson et al, 2014). Preoperative variables were found to predict suboptimal surgical outcomes, and one was poor dietary adherence such as LOC eating. Interventions targeting episodic behaviors that influence obesity that is treatment-refractory thus demand consideration and rigorous study.

Responsive neurostimulation, or RNS, is now a standard FDA-approved treatment for well-selected cases of refractory epilepsy, and holds great promise for other disorders of the brain, because of its ability to detect and respond to a predefined electrographic pathophysiological signature. Such a candidate electrographic biomarker for RNS for LOC eating has already been characterized preclinically (Wu et al, manuscript in press for *PNAS*; Wu et al, 2016). Notably, the NAc will only be stimulated intermittently, and based on NeuroPace's experience in epilepsy and on the Stanford Research Group's mouse model, the total amount of stimulation time "on" is anticipated to be less than 5 minutes per day.

FDA-approval for the RNS® System was based on data from 2 clinical trials including a feasibility study to demonstrate safety (N=65) and a 2-year multicenter double-blind randomized controlled pivotal trial to assess safety and efficacy (n=191). These studies demonstrated RNS of defined seizure-genic structures is safe and effective at controlling epilepsy severity (Morrell et al, 2011). As of 12/01/15, mean follow-up was almost 7 years with an accumulated 1715 patient implant years and 1613 stimulation years (Bergey et al, 2015). These studies found the device to be well-tolerated, and efficacy was significantly improved at long-term follow-up. Moreover, significant improvements in naming, verbal learning, and quality of life were reported, and mood was not negatively impacted (Loring et al, 2015; Meador et al, 2015). Although the large majority of these implants are placed in the mesial temporal structures including the amygdala (Heilbronner et al, 2016), we propose to target

the NAc with RNS, which is a different region though it receives direct projections from the amygdala. Given no adverse changes in mood have been seen in RNS for epilepsy, we are reassured that RNS applied to areas involved in reward processing is safe (Meador et al, 2015).

### 1.3 Loss of control over eating in treatment-refractory obesity: Target patient population

Loss of control over (LOC) eating is extremely common in obesity and complicates treatment in all binge eaters. Animal, viral tracing, and slice electrophysiology studies, as well as neuroimaging studies in humans, have provided insight into the neuroanatomy and pathophysiological mechanisms of LOC eating, which is largely due to the hedonic properties of calorically dense food mediated by the dopamine system's projections to the NAc (Beaver et al, 2006). The obese state has been reported to be associated with attenuated dopamine signaling in the NAc that predispose to LOC eating (Teegarden and Bale, 2007).

### 1.4 Role of NAc in gating loss of control over eating

The scientific basis for the NAc-RNS approach taken here has evolved over an ongoing 10-year period of study lead by Dr. Casey Halpern. These efforts include multidisciplinary investigations to examine the unmet medical and socioeconomic needs in obesity, the pathophysiological and neuroanatomical mechanisms of impulsivity towards food, the ethical implications for trial development for this and similar conditions, and translation of RNS to human subjects with LOC over food and associated treatment-refractory obesity (Halpern et al, 2008; Pisapia et al, 2010; Halpern et al, 2011; Attiah et al, 2012; Halpern et al, 2013; Pisapia et al, 2013; Halpern et al, 2014; Ho et al, 2015; Ali et al, 2016; Wu et al, 2016; Cartmell et al, 2017; Wu et al, in press).

One potential predictor of bypass failure is binge-like behaviors due to LOC eating, which is seen in at least 50% of bypass patients (Hsu et al, 1997; White et al, 2010). This is likely due, at least in part, to decreased dopamine type 2 receptor availability in portions of the striatum, including the NAc, which may predispose to LOC eating to compensate for attenuated dopamine signaling (Broft et al, 2012; Johnson and Kenny, 2010; Stice et al, 2008; Wang et al, 2001). What this manifests as is impulsivity for anticipated incentives, such as highly palatable food, to which obese patients are known to be hypersensitive. Temporally specific activity within the NAc has been reported to occur during anticipatory periods preceding consummatory behavior. This period represents a critical opportunity for intervention, but no available therapy is capable of automatically sensing and therapeutically responding to this vulnerable moment in time when anticipation-related neural signals may be present.

### 1.5 Preclinical Data

A preclinical trial, utilizing a mouse model of limited-exposure to high fat food (HF) known to induce binge-like LOC eating, found that electrically stimulating the NAc (130Hz, 60us, 150uA) immediately before and during exposure to a high-fat (HF) diet attenuated the subsequent binge (Fig. 9.1), and this effect exhibited an expected dose response (Halpern et al, 2013). Notably, chronic studies in obese mice revealed reduced intake with continuous stimulation like DBS, but a tolerance effect was evident (Fig. 9.1c). Nevertheless, NAc stimulation using the same parameters did not induce a place preference, suggesting binge blockade did not involve reward substitution (Fig. 9.1d).

Binge eating is episodic in mice much like LOC eating in humans (Fig. 9.1e). This episodic behavior, combined with an apparent tolerance effect to continuous stimulation supports stimulating only when necessary. Moreover, chronic continuous stimulation appeared to alter normal social interaction behavior in mice, supporting further the need for responsive, intermittent stimulation. Indeed, we saw binge blockade in our preliminary studies with manually administering stimulation only during approach to HF food with >90% decrease in the cumulative dose of current (i.e. percent time-on) (Fig. 9.1f). However, in order to translate this to humans, a device capable of anticipating an upcoming binge is needed. The Stanford closed-loop rig integrated a prototype NeuroPace RNS device to test whether this already approved system could effectively detect a physiological predictive biomarker of a HF meal in a mouse.

#### 1.5.1 Identifying a predictive biomarker

To identify translatable biomarkers for an off-the-shelf RNS system, Stanford recorded local field potentials from the NAc of mice anticipating food reward (Fig. 9.2). This study utilized a model of limited exposure to HF food known to induce LOC-like eating within 10 days of the initial exposure (Halpern et al, 2013). Recordings of real-time LFP activity from the mouse NAc using a RNS-prototype revealed increased power in low frequency oscillations (delta) prior to binge onset (Fig.9.2 ). Delta specificity was assessed by examining LFPs prior to chow intake and social interaction, which were not associated with increased delta power (Fig.9.2).

#### 1.5.2 Pre-clinical Studies of NAc-RNS in mice

The preclinical studies examined multiple stimulation paradigms to assess potential efficacy of our proposed intervention: (1) continuous electrical stimulation during the entire 1 hour exposure to high fat food, a pattern of stimulation that mimics DBS (DBS; 130 Hz, 0.1 mA, bipolar, biphasic), (2) manually-triggered stimulation during which an experimenter remotely observes the behavior via video-monitoring and triggered the electrical stimulation (130 Hz, 0.1 mA, 10 seconds, bipolar, biphasic stimulation) at the immediate onset of a binge, (3) responsive neurostimulation during which

detection of a pre-defined oscillatory change (Fig. 9.2) triggered electrical stimulation (130 Hz, 0.1 mA, 10 seconds, bipolar, biphasic stimulation), and (4) random stimulation during which bouts of stimulation (130 Hz, 0.1 mA, 10 seconds, bipolar, biphasic stimulation) were delivered randomly throughout the entire 1-hour HF exposure such that the total number of stimulation bouts matched that delivered during the RNS protocol. All the stimulation protocols significantly reduced HF intake except random stimulation [Fig. 9.3C-F; DBS $T(5) = 2.58$, $P < 0.05$]. The reduction of HF intake was compared between each stimulation protocol. The reductions in HF intake induced by manual stimulation and RNS were significantly more robust than random stimulation ($F(4) = 7.034$, $P < 0.01$). Furthermore, the number of bouts of stimulation used for manual and RNS were lower than DBS ($F(1.566, 7.813) = 65.80$, $P <0.0001$) (Fig. 9.3F).

A number of behavioral assays were conducted to determine whether RNS of the NAc might have detrimental side effects. RNS using the same delta band power threshold as a trigger had no significant effect on interaction time (Fig. 9.3G), while DBS of the NAc significantly reduced time spent socially interacting in a juvenile interaction task ($F(2,21) = 4.557$, $P < 0.05$). Spontaneous locomotor behavior during the limited-exposure HF protocol was not affected by DBS nor RNS (Fig. 9.2H; $F(1.699, 8.493) = 0.891$, $P = 0.429$). Furthermore, NAc stimulation (130 Hz, 0.1 mA, continuous, bipolar, biphasic stimulation) did not induce real-time place preference, suggesting that blocking LOC eating did not involve reward substitution (Fig. 9.3I; $T(5) = 0.2283$, $P = 0.8285$). These results suggest that RNS of the NAc is neither reinforcing nor aversive, and its effects can block consumption of HF food while sparing normal locomotor and social behaviors.

### 1.6 Preliminary findings in humans

A study was performed, using an identical external RNS prototype, to record from the human NAc. Because in the operating room food rewards could not be provided, the study team elicited anticipation of monetary rewards with a well-established neuroimaging task (i.e., the Monetary Incentive Delay (MID) task) that preoperatively evoked BOLD signal responses in the NAc of the very same patient subject. Similar increases in power in the delta band were found that were specific to large reward anticipation that were not seen for smaller rewards or loss of gains (Fig. 9.4). Our preliminary human findings support that this oscillatory range appears active during high reward anticipation, and carries reward-specific specificity that should allow us to sense food reward anticipation out of the context of normal meals, as we saw in our preclinical model.

## 2. DEVICE DESCRIPTION

The RNS®System has been approved by the FDA for adjunctive treatment of adults with medically refractory partial-onset seizures related to no more than 2 epileptogenic foci. The RNS®System was approved via PMA P1000026, and no changes have been made for use in this study.

### 2.1 RNS® System Product Description

This system is the first FDA-approved, intracranial, closed-loop device, and includes a cranially-implanted responsive neurostimulator connected to 2 leads, a physician programmer, patient remote monitor, and an internet-based data repository for physicians to review stored electrographic data. This system is commercially available and utilized routinely for certain refractory epilepsy patients. The RNS® System has demonstrated safety and effectiveness in adults with medically refractory seizures.

The RNS® System is a novel, implantable therapeutic device that delivers responsive neurostimulation, an advanced technology designed to continuously monitor brain electrical activity, detect abnormal electrical activity and respond by delivering imperceptible levels of electrical stimulation to normalize that activity before an individual experiences seizures. The RNS® System includes implantable and external components:(Fig. 9.5):

- The implantable components are the RNS Neurostimulator and leads. The neurostimulator is a battery-powered, microprocessor-controlled device that is placed within the skull and beneath the scalp by a surgeon. It is connected to one or two leads that are placed within the brain or rest on the brain surface in the area of the seizure focus. These leads can be either a depth lead (if the focus is deep brain) or a cortical strip lead, which is placed on the cortex (if the focus is cortical). The neurostimulator and leads are implanted by a neurosurgeon during a procedure that typically takes two to three hours. For this study, we will be using the depth leads (DL-330-3.5) in order to target the NAc with tightly spaced contacts. Stimulation is delivered to a maximum of two implanted leads (one lead per hemisphere), with a maximum of 4 electrodes per lead. Rate is limited to 333 Hz, pulse width is limited to 1000 μsec, amplitude is limited to 12 mA and the charge density is limited to at or below 25 μC/cm2/phase.
- External components include a NeuroPace® Programmer, a NeuroPace® Remote Monitor, and a magnet. Both devices use proprietary software that enable communication

with an implanted RNS Neurostimulator. Physicians use the programmer to non-invasively program the detection and stimulation settings of an implanted RNS Neurostimulator to customize therapy for each individual. Physicians also use the programmer to retrieve data stored in the neurostimulator and transmit data to the NeuroPace® Patient Data Management System (PDMS), an interactive web-based database used for storage and clinician remote access. Patients use the remote monitor at home to transmit recordings of their brain electrical activity and other information stored in the neurostimulator to the PDMS. Physicians can review and analyze this information over the internet between the patient's office appointments to help with patient management.

The neurostimulator is programmed for initial use after the RNS® System is surgically implanted. For this particular trial, the initial programming will be performed by a psychiatrist with expertise in stimulation of the human NAc. Afterwards, the neurostimulator settings are adjusted on an ongoing basis as needed. A computer (called the NeuroPace® Programmer) allows the physician to perform the initial programming and follow-up adjustments to the neurostimulator. Adjustments are based on brain activity and response to stimulation, which are both stored in the neurostimulator.

A remote monitor lets the patient collect data from the neurostimulator. The remote monitor consists of a special software program installed on a laptop computer, a wand and accessories. After connecting the hand-held wand to the laptop, data in the neurostimulator are collected by placing the wand over the implant site. The wand uses Radio Frequency communication to collect the data, and send the data to a secure database. Data are stored in the laptop and then sent to a secure database over the internet. The database is called the PDMS (Patient Data Management System) and the treatment team can access the data remotely. The psychiatrist in concert with the sponsor and NeuroPace field engineers will review the data and use the results to adjust the neurostimulator settings during future office visits.

## 3. OBJECTIVES AND ENDPOINTS

### 3.1 Study Objectives

The primary objective of this trial is to assess device function and safety, with secondary objectives including the feasibility of the assessments and to inform an

advanced clinical study. The study objectives include:

- Feasibility of responsive neurostimulation by investigating NAc physiology and possible correlation with LOC eating using time series and spectral analysis methods.
- Biomarker identification, including the function of biomarker specific detection algorithms.
- Safety of the RNS System and NAc stimulation through evaluation of AE's, Patient Reported Outcome questionnaires, psychiatric interviews, C-SSRS data, and neuropsychological function.
- Proof-of-concept data suggestive of benefit in at least half of the patient participants with the proposed intervention as determined by a percentage change in frequency of LOC from baseline, body weight and serum markers of obesity.

### 3.2 **Study Endpoint**

- The primary efficacy endpoint will be assessed by at least 50% of subjects exhibiting a decrease in the number of LOC episodes per week. LOC episodes will be assessed by the currently available standard, ecological momentary assessment (EMA) diary entries using a smartphone.

## 4. **STUDY DESIGN**

### 4.1 Overview and Design

This is an Early Feasibility Study determining the safety and efficacy of NAc-RNS in the treatment of LOC eating in treatment-refractory obesity. Following rigorous consent and screening processes, obese subjects with LOC who have also failed gastric bypass surgery will be implanted with the RNS System (NeuroPace, Inc.) with depth leads in the NAc. This surgery will require a standard awake stereotactic procedure targeting the NAc. Once the NeuroPace depth leads and neurostimulator are implanted, the recording-only phase will be initiated. Subjects will maintain time-stamped logs of eating patterns using 3 ambulatory assessments: 1) ecological momentary assessment (EMA), 2) a commercially available wrist-worn bite counter, and 3) magnet swiping. Subjects will be instructed to swipe the NeuroPace magnet over the cranially implanted neurostimulator to trigger LFP storage whenever LOC is sensed, similar to what epilepsy patients routinely do when they sense a seizure. The neurostimulator will be programmed to store LFP data in response to 1) this magnet swipe, 2) scheduled times, and 3) detecting the Stanford candidate LFP biomarker. Behavioral laboratories

and computer tasks will be utilized to examine LOC eating and optimize the LFP biomarker and stimulation testing in controlled settings with video synchronized to the RNS System recording. Then, a 12-month responsive stimulation phase will test feasibility/safety of intermittently delivery stimulation to the NAc.

4.2 **Subject Selection**

Subjects will be selected for this trial if they are morbidly obese with LOC eating (≥4 LOC episodes per week or 16 per month), have failed gastric bypass surgery, and meet all other inclusion-exclusion criteria. In assessing the technical result of the bypass, an upper GI series will be obtained in all patients enrolled and we will compare the height and width of the gastric pouch as well as the diameter of the anastomosis with the height of the vertebral body. If deemed suitable for enrollment, the bariatric team will proceed with referring to our study. If the patient proceeds through all screening visits and wishes to proceed with surgery, an esohagogastroduodenoscopy will be performed to confirm the pouch size and more definitely assess the diameter of the anastomosis as well as rule out ulcers.

Notably, there are no standard of care options for these obese patients who have failed all available therapies. Once technical failures in the bypass surgery are ruled out, there is little evidence from controlled trials that available adjunctive therapies (i.e., patient support groups, dietary modification, behavioral therapy, or weight loss medications) are of benefit in inducing weight loss or preventing further weight regain. Given this unmet medical need, we are proposing this investigational option. Re-attempting failed therapies or introducing an available new therapy as an adjunct (e.g. weight loss medication) to what we are investigating would go beyond current practices. However, if the patient has enrolled in support groups and would like to continue this option, they could do so while participating in the trial.

Subjects will be recruited through the Bariatric and Metabolic Interdisciplinary Clinic. A retrospective chart review of gastric bypass failure patients, as well as local advertising (Stanford clinical trials website, clinic brochures, etc.), will occur in order to ease recruitment. A systematic and multi-disciplinary approach to our patient assessment will be used with a focus on contributory dietary, psychologic, medical, and surgical factors. This is standard in how patients are prepared for bariatric surgery at Stanford, thus we will leverage this already-in-place protocol in assessing patients

for this study.

Specifically, all patients getting enrolled into our study will have a consultation with a nutritionist, and a nutrition panel to assess vitamin deficiencies, thyroid stimulating hormone, lipids, HgbA1C will be performed. Patients will also have an eating disorder psychological assessment by our psychiatrist with expertise in this bariatric population. This is a comprehensive hour-long assessment performed in all bariatric patients at Stanford to assess for contributing psychological/psychiatric factors. In addition to confirming the presence of LOC eating, the psychiatrist will use a tool developed at Stanford that is now standard for all bariatric patients, the Stanford Integrated Psychosocial Assessment for Bariatric Surgery, adapted from the Stanford Integrated Psychosocial Assessment for Transplantation (Maldonado et al. 2012). Using this tool, psychological stability, the presence of psychopathology, personality disorders, and substance abuse will be assessed to complement our battery of questionnaires. Contact with current providers may be needed to best assess and confirm stability of any ongoing treatments, and treatment history will be reviewed. A critical component to this assessment will be to examine personality traits and their potential influence to assess adherence and compliance to ecological momentary assessment. Lastly, an upper gastrointestinal series would be performed prior to referral to this study and evaluated by one of our bariatric surgery collaborators to confirm the absence of a gastro-gastric fistula, the anastomosis size ≥ 2 cm and/or gastric pouch size of ≥ 6 cm in length and ≥ 5 cm wide. If this is not well assessed on the upper gastrointestinal series, an esophagogastroduodenoscopy would be requested. The upper gastrointestinal series would also allow them to rule out obstruction, stricture or other anomaly that would require further investigation with esophagogastroduodenoscopy.

### 4.2.1 Inclusion Criteria

1. Male and female patients, 22 to 64 years of age, inclusive.2. BMI 45-60 kg/$m^2$
2. Failure of at least one pharmacological agent intended for weight loss or binge eating disorder (minimum trial of 6 months), one form of behavioral therapy (such as weight loss therapy and cognitive behavioral therapy; minimum trial of 6 months), and gastric bypass surgery. Failed bariatric surgery is determined using the modified Reinhold classification as patients who lost less than 50% of excess weight by 24 months after a technically successful surgery.
3. Assessment by a Stanford bariatric surgeon prior to referral to this study to rule out technical explanations for suboptimal outcome with an upper gastrointestinal

series within the months prior to consent (i.e. a pre-study referral assessment). The upper gastrointestinal series would be evaluated by a team bariatric surgeon for 3 purposes: 1) to rule out an obstruction, stricture or gastro-gastric fistula, or other anomaly; 2) to confirm the anastomosis size ≥ 2 cm and/or gastric pouch size of ≥ 6 cm in length and ≥ 5 cm wide. If these features characteristic of technical failures cannot be well assessed on the upper gastrointestinal series, an esophagogastroduodenoscopy would be requested.

4. Presence of Loss of Control over eating (≥4 LOC episodes per week or 16 per month (i.e. 28 days) and confirmed with a clinical evaluation by a Stanford eating disorder specialist.
   - The assessment of the presence of LOC will be made during the initial screening visit based on assessments of binge-like eating episodes ascertained from the Eating Disorder Examination (EDE) interview. Subjects will be asked to report the numbers of LOC episodes per week they recall over the past 28 days. All standard measures, including the EDE (which is the gold standard measure used in all major studies of eating disorders) depend on patient recall. While self-report is a limitation, studies have found the measure to be reliable (Mason et al. 2017; Berg et al. 2015). Both objective binge episodes (defined as eating unusually large amounts of food while experiencing a subjective sense of LOC) and subjective binge episodes (defined as experiencing LOC when eating small or normal amounts of food) will be classified as LOC episodes.

   - This recollection will be confirmed using a validated scale assessing LOC features (that is, the Eating Loss of Control Scale, ELOCS) (Blomquist et al. 2014). This scale has revealed significantly convergent validity and correlated with the Eating Disorder Examination Questionnaire (EDE-Q) and the Eating Disorder Examination Interview (EDE)(Fairburn and Beglin 1994).

   - Item 2a from the ELOCS is the most relevant question for this inclusion criterion and is copied here:
     - *During the past four weeks, how many times have you felt helpless to control your eating urges?*

5. Any medical (including psychiatric) conditions must be monitored actively by appropriate discipline and stable for the past 6 months. Related therapies or medications should be held stable for the study duration.
6. Surgical suitability confirmed by a psychiatric examination.
7. Subject is able to attend all scheduled clinic appointments on their own or with a caregiver.
8. Adequate social support (e.g. stable housing, identified family member or close friend as emergency contact) without acute or subacute psychosocial stressors based on screening interview.
9. Premenopausal women must agree to use acceptable methods of birth control.
10. Participants provide voluntary, decisionally capable, and appropriately informed consent. Subject is able to comply w/ all testing and follow-up requirements

defined by the study protocol.
11. Participant has no immediate plan for relocation beyond 6 hours of the study site.
12. Proficiency with the English language.

**4.2.2 Exclusion Criteria**

1. Subject has an implanted medical device that delivers electrical energy to the brain.
2. Subject has an implantable cardiac pacemaker, defibrillator, or neurostimulator.
3. Subject requires diathermy treatments.
4. Subject requiring transcranial magnetic stimulation or electroconvulsive therapy should be excluded.
5. Subject is likely to require repeat MR imaging after implant of the RNS Neurostimulator and Leads.
6. Subject is unable to fit into CT scanner (500lb upper weight limit for CT scanner).
7. Subject is pregnant or intends to become pregnant during the course of the study.
8. Subject is participating in a therapeutic investigational drug or device study.
9. Medical contraindications for surgery including but not limited to severe cardiovascular, pulmonary, renal, liver, hematological disease, severe coagulopathy, or an acute infectious process.
10. Evidence of neurological disorders, e.g. seizure disorder, multiple sclerosis, severe acquired brain injury, severe brain atrophy, subdural hematoma, history of hemorrhagic stroke, or other clinically relevant abnormality on preoperative imaging.
11. Current physical or medical condition that could affect eating behavior (e.g., cancer, pregnancy).
12. Active use of medication known to affect eating (e.g., appetite suppressants).
13. Clinically significant or unstable psychiatric condition based on psychiatric screening interview.
14. Clinical diagnosis (past or present) severe anxiety disorder, major depression, psychosis, or anorexia based on diagnostic interview.
15. Any lifetime history of suicide attempt, intent or engagement in other forms of self-harm behaviors (e.g. cutting).
16. History of drug abuse or dependence, including nicotine and alcohol.
17. Current use of alcohol at the rate of > 14 drinks per week or > 4 drinks per occasion or any diagnosis of substance abuse/dependence disorder based on DSM-5.
18. Evidence of incipient dementia or cognitive impairment on Neuropsychological assessment by any score on memory, executive functioning, intellectual functioning, language, or visuospatial domains falling 2SD below the

normative mean.

19. Evidence of comprehension difficulties (Token Test <36).
20. Inability to provide informed consent to treatment.
21. Obesity secondary to another medical condition, a medication side effect, or a genetic syndrome.
22. Less than 80% compliance with ecological momentary assessment at the baseline/Pre-Op visit.
23. Patients who are candidates for revision of their bariatric surgery.

## Schedule of Events

| Closed-Loop Neurostimulation for Loss of Control Eating | Expected time (min) | Safer LOC Interview | General Screening | Baseline/Pre-Op | Surgery | Post-Implant appointments | | | | | | | | | | | | | | | | | |
|---|---|---|---|---|---|---|---|---|---|---|---|---|---|---|---|---|---|---|---|---|---|---|---|
| | | | | | | Recording | | | | | | Stimulation Testing | | | | | | | | | | | |
| | | | | | 0 | 1 | 2 tc | 3 | 4 | 5 | 6 | 7 | 8 | 9 | 10 | 11 | 12 | 13 | 14 | 15 | 16 | 17 |
| Videoed informed consent process (offline ethics review by LD and LR) | 45 | ✓ | | | | | | | | | | | | | | | | | | | | | |
| MacCAT-CR | 15 | ✓ | | | | | | | | | | | | | | | | | | | | | |
| Eating disorder assessment | 30 | ✓ | | | | | | | | | | | | | | | | | | | | | |
| EDE-Q | 10 | ✓ | | | | ✓ | | ✓ | | | ✓ | ✓ | ✓ | ✓ | ✓ | ✓ | ✓ | ✓ | ✓ | ✓ | ✓ | ✓ |
| Grilo LOC (ELOCS) | 10 | ✓ | | | | ✓ | | ✓ | | | ✓ | ✓ | ✓ | ✓ | ✓ | ✓ | ✓ | ✓ | ✓ | ✓ | ✓ | ✓ |
| Dutch Eating Behavior Questionnaire-Emotional Eating subscale | 10 | ✓ | | | | ✓ | | ✓ | | | ✓ | ✓ | ✓ | ✓ | ✓ | ✓ | ✓ | ✓ | ✓ | ✓ | ✓ | ✓ |
| Beck Depression Inventory-2 (BDI-II) | 10 | ✓ | | | | ✓ | | ✓ | | | ✓ | ✓ | ✓ | ✓ | ✓ | ✓ | ✓ | ✓ | ✓ | ✓ | ✓ | ✓ |
| C-SSRS | 10 | ✓ | | | | ✓ | | ✓ | | | ✓ | ✓ | ✓ | ✓ | ✓ | ✓ | ✓ | ✓ | ✓ | ✓ | ✓ | ✓ |
| SCID1&2 | 90 | ✓ | | | | | | | | | | | | | | | | | | | | | |
| Handheld EMA intro | 15 | ✓ | | | | | | | | | | | | | | | | | | | | | |
| Pregnancy Test (urine) | 1 | ✓ | | | | | | | | | | | | | | | | | | | | | |
| Urine Tox Screen | 1 | ✓ | | | | | | | | | ✓ | | ✓ | | | ✓ | | | ✓ | | | ✓ |
| Body weight | 1 | ✓ | | ✓ | | ✓ | | ✓ | | | ✓ | ✓ | ✓ | ✓ | ✓ | ✓ | ✓ | ✓ | ✓ | ✓ | ✓ | ✓ |
| Vitals | 1 | ✓ | | | | ✓ | | ✓ | | | ✓ | ✓ | ✓ | ✓ | ✓ | ✓ | ✓ | ✓ | ✓ | ✓ | ✓ | ✓ |
| Fasting Glucose, Glucose Tolerance Testing | 1 | ✓ | | | | | | ✓ | | | ✓ | | ✓ | | | ✓ | | | ✓ | | | ✓ |
| Nutrition Panel (e.g. chemistry, lipids, A1C) | 1 | ✓ | | | | | | ✓ | | | ✓ | | ✓ | | | ✓ | | | ✓ | | | ✓ |
| General Demographics | 5 | ✓ | | | | | | | | | | | | | | | | | | | | | |
| Review disease history | 10 | ✓ | ✓ | | | | | | | | | | | | | | | | | | | | |
| Review medical history | 10 | ✓ | ✓ | | | | | | | | | | | | | | | | | | | | |
| Review concomitant treatments (other meds) | 5 | ✓ | ✓ | ✓ | ✓ | ✓ | ✓ | ✓ | ✓ | ✓ | ✓ | ✓ | ✓ | ✓ | ✓ | ✓ | ✓ | ✓ | ✓ | ✓ | ✓ | ✓ |
| Adverse Event Review | 5 | | | ✓ | | ✓ | ✓ | ✓ | ✓ | ✓ | ✓ | ✓ | ✓ | ✓ | ✓ | ✓ | ✓ | ✓ | ✓ | ✓ | ✓ | ✓ |
| Nutrition appointment | 30 | | ✓ | | | | | | | | | | | | | ✓ | | | | | | ✓ |
| EMA compliance review | 10 | | ✓ | ✓ | | | | | | | | | | | | | | | | | | |
| Neurological/Physical exam | 10 | | ✓ | ✓ | | ✓ | | ✓ | | | ✓ | ✓ | ✓ | ✓ | ✓ | ✓ | ✓ | ✓ | ✓ | ✓ | ✓ | ✓ |
| Structured psychiatric interview | 30 | | ✓ | | | | | | | | | | | | | | | | | | | |
| Interventional psychiatric evaluation | 30 | | ✓ | ✓ | | ✓ | | ✓ | | ✓ | ✓ | ✓ | ✓ | ✓ | ✓ | ✓ | ✓ | ✓ | ✓ | ✓ | ✓ | ✓ |
| Neuropsychology (formal) | 60 | | ✓ | | | | | | | | | | | | | | | | | | | ✓ |
| Bariatric Monitoring Visit | 40 | | | | | | | ✓ | | | | | | | | | | | | | | |
| LOC EMA review (1 week per month of EMA) | 5 | | ✓ | ✓ | | ✓ | ✓ | ✓ | ✓ | ✓ | ✓ | ✓ | ✓ | ✓ | ✓ | ✓ | ✓ | ✓ | ✓ | ✓ | ✓ | ✓ |
| Ambulatory bite-counter intro | 10 | | ✓ | | | | | | | | | | | | | | | | | | | |
| Bite-counter review | 10 | | | ✓ | | ✓ | ✓ | ✓ | ✓ | ✓ | ✓ | ✓ | ✓ | ✓ | ✓ | ✓ | ✓ | ✓ | ✓ | ✓ | ✓ | ✓ |
| Ambulatory bite-counter intro (1 week alternating with EMA) | 10 | | ✓ | | | ✓ | ✓ | ✓ | ✓ | ✓ | ✓ | ✓ | ✓ | ✓ | ✓ | ✓ | ✓ | ✓ | ✓ | ✓ | ✓ | ✓ |
| Monetary Incentive Delay | 20 | | | ✓ | | | | | | ✓ | | ✓ | | | ✓ | | | | | | | |
| Milkshake paradigm | 20 | | | ✓ | | | | | | ✓ | | ✓ | | | ✓ | | | | | | | |
| Multi-Item Buffet (includes questionaires and nutritionist) | 360 | | | | | | | | | ✓ | | ✓ | | | ✓ | | | | | | | |
| Fidicial placement | 30 | | | ✓ | | | | | | | | | | | | | | | | | | |
| SAU preop | 30 | | | ✓ | | | | | | | | | | | | | | | | | | |
| MRI with and without contrast | 60 | | | ✓ | | | | | | | | | | | | | | | | | | |
| CT Brain | 15 | | | ✓ | ✓ | | | | | | | | | | | | | | | | | |
| Monopolar assessment | 60 | | | | ✓ | | | | | ✓ | | | | | | | | | | | | |
| Initiate recording | 5 | | | | ✓ | | | | | | | | | | | | | | | | | |
| Distribute magnet/remote monitor training | 60 | | | | ✓ | | | | | | | | | | | | | | | | | |
| Manage neurostimulator/programming | 15 | | | | ✓ | ✓ | | ✓ | ✓ | ✓ | ✓ | ✓ | ✓ | ✓ | ✓ | ✓ | ✓ | ✓ | ✓ | ✓ | ✓ | ✓ |
| Wound check | 5 | | | | | ✓ | | ✓ | | | | | | | | | | | | | | |

### 4.3 Study Assessments

- MacArthur Competence Assessment Tool for Clinical Research (MacCAT-CR) -- This semi-structured interview instrument is designed to assist the assessment of capacity to consent to clinical research participation. It can be adapted for specific protocols. As directed MacCAT-CR developers, the tool will be administered after a full consent discussion. Each consent and MacCAT-CR session will be videotaped (a separate consent for videotaping will be performed) for post-hoc review by our Ethics Advisory Committee.
- General Demographics Sheet--Each subject will be assigned an independent ID number. The following information will be collected: age, weight, BMI, disease duration, all medical comorbidities, all medications and dosages, family history, smoking history, alcohol history and substance abuse history.
- Eating Disorder Examination (EDE) Questionnaire--The EDE-Q was adapted from the EDE and is a 28 item self-report questionnaire and has exhibited significant convergence with the EDE. It focuses on behaviors over a 28 day time period.
- Grilo Eating Loss of Control Scale (ELOCS) --The ELOCS is a self-report questionnaire that examines LOC-related behaviors on continuous Likert-type scales and the number of LOC episodes in the past 28 days. Given that LOC is a central diagnostic and clinical feature of multiple eating disorders, this comprehensive, validated, self-report measure of LOC was created to capture the varied experience of LOC among individuals by measuring different aspects of this construct as well as severity on continuous Likert- type scales. This scale does not diagnose the presence of LOC – this is done dichotomously (yes/no) and during a structured clinical interview for an eating disorder assessment, facilitated by the EDE-Q and ELOCS described above.
- Dutch Eating Behavior Questionnaire (DEBQ) –The scale on emotional eating has 2 subscales (1) eating in response to diffuse emotions and (2) eating in response to clearly labelled emotions.
- Beck Depression Inventory-2 (BDI-II; 70) – A brief self-report scale of depression.
- Structured Clinical Interview (SCID1&2; 50): This is a clinician-administered comprehensive and diagnostic psychiatric assessment of DSM-5 Axis I and II disorders.
- Columbia Suicide Severity Rating Scale (C-SSRS) – A scale that rates an individual's degree of suicidal ideation, ranging from "wish to be dead" to "active suicidal ideation with specific plan and intent."
- Neuropsychology Assessment – This 1-hr battery was designed by our clinical neuropsychologist, customized for LOC-related disorders, and assesses: executive functioning (CANTAB Go/ No-Go Test; cognitive flexibility and attention (Trail Making Test A &B; WMS-III Digit Span), working

memory (WMS-III mental control), memory (WMS-III Logical Memory and Hopkins Verbal learning Test (HVLT-R); language (Token Test), and overall neurocognitive functioning level (Dementia Rating Scale-2). Testing will be completed by a trained research assistant under direct supervision of the same neuropsychologist that designed this battery with an emphasis on identifying surgery readiness. Score interpretation and report writing will be performed by the neuropsychologist.

- Ecological Momentary Assessment (EMA)28:EMA allows for real-time assessment of variables of interest in the natural environment (minimizing retrospective response bias), and the repeated assessment of key variables multiple times per day for a period of days or weeks. Prior to surgery, after being trained on the EMA, participants will gain access to a web-based EMA compatible with all smartphones, and asked to practice for 1 week (http://retaine.org). Data review will occur remotely by a trained research coordinator and discussed with each subject by phone. After this practice period, participants complete EMA assessments over a 1-week compliance check period. After this 2-week assessment, a visit is scheduled with each participant, and participants are provided feedback. Surgery will then take place. So as to ensure compliance throughout the 18-month study, participants will be asked to maintain EMA data for only 1 week per month to minimize the burden of assessments. Assessment of LOC will involve several types of recordings to maximize the ability to capture episodes of LOC. These will include signal, interval contingent, and behavior contingent recordings. Signal contingent recording includes having the participant record their experiences whenever signaled at semi- random times by the researcher. In this study, participants will be signaled 5 semi- random times per day. Each response will require 2-3 minutes to complete. Compliance will be assessed by how often patients respond to signaled recordings (minimum inclusion criteria of 80% compliance). Behavior contingent recordings refer to having participants complete recordings before and after they engage in behaviors rather than in response to a text. Interval contingent recording includes recording at regular intervals determined ahead of time by the researcher. In this study, participants will complete an end of day recording as well. During monthly follow-ups, they will be queried about the details of each eating episode as well. All data will be automatically time- and date-stamped on smartphones and saved in a secure online database, providing the opportunity to delineate temporal relationships with LFP recordings and concordance with other assessments. Consistent with previous investigations, our EMA assessment protocol implements a daily self-report protocol related to LOC eating:
    - LOC eating logs -- Participants are asked to rate mood, stress, hunger, and LOC immediately before and after any eating episode. Negative mood is assessed using an abbreviated Positive and Negative Affect Scale (PANAS) as LOC eating is typically preceded by negative emotion. This scale comprises the sum of 5 items (afraid, scared, upset, distressed, and jittery), all of which are rated on a 5-point scale. Ratings for pre- meal LOC are also made on a 5-point

Likert-type scale. A rating of 1 on the scale corresponds to "complete control" and 5 signifies "complete LOC"; postmeal, participants respond to a "yes/no" question as to whether they had experienced LOC while eating. In addition, participants will be signaled at semi-random intervals several times a day to ask retrospectively about any episodes of LOC that may have taken place since the last signal. If the participant did not already fill out ratings about that episode, they are asked to do so at that time. In addition, participants are asked to fill out an end-of-the-day record to ensure that any and all LOC episodes are recorded.

- <u>Bite-Count Measure of Eating Activity</u> -- The bite counting device (Bite Counter, Bite Technologies) is a 64x38x25 mm black plastic rectangle weighing 75 g with an adjustable wrist band. The device operates as a digital watch that also connects to a USB port for uploading to a server for remote data review. At the beginning of each eating activity (normal and LOC meals), the users will be asked to press a button to activate “bite-count” mode. At the end of each eating activity, the user presses a button to return to “time” mode. In addition, the device automatically returns to “time” mode after 1 hr of operating in “bite-count” mode. The device is fully rechargeable and saves the date, time, duration, and bite count for each eating activity. The device has 90 percent sensitivity for bites in an ambulatory setting. Bite count directly correlates with kilocalories consumed. The use of body sensing for directly monitoring eating has only recently begun to be explored, but this device has an inconspicuous nature which we feel will make it feasible to incorporate into ambulatory assessments of LOC eating. It provides additional timestamps so that we can assess LFPs during eating episodes. Participants will be asked to utilize the bite counter 1 week/month throughout the clinical study (never simultaneously with EMA so as to avoid over-burdening participants). Like the EMA, data review will occur remotely by a trained research coordinator and discussed with each subject by phone. After this 2-week assessment, a visit is scheduled with each participant, and participants are provided feedback.
- <u>Milkshake Paradigm</u> --This laptop task was designed by Stanford psychiatrist (Dr. Cara Bohon) to examine neural activity during anticipation of a palatable taste (chocolate milkshake). Participants are asked to refrain from eating or drinking caffeinated beverages for 5 hours preceding their appointment. Stimuli are 2 images (glasses of milkshake and water) that signal impending delivery of either 0.5 mL of chocolate milkshake or tasteless solution. Cues are presented for

2 s and are followed by a jitter of 1–7 s during which time the screen is blank. On 40% of the trials the taste is not delivered after the cue to allow investigation of the neural response to anticipation of a taste not confounded with actual receipt of the taste. Tastes are delivered with programmable syringe pumps. Syringes filled with milkshake and tasteless solutions are connected via Tygon tubing to a manifold that fits into participants' mouths. Participants are instructed to swallow when the word "swallow" appears. During the paradigm, real- time video and LFP activity will be recorded by the RNS and assessed offline (and in real-time) for candidate biomarkers of expected milkshake.

- Monetary Incentive Delay (MID) -- This task was designed by Stanford investigator, Dr. Brian Knutson, and examines neural activity during anticipation of monetary gain. As individuals may vary in their affective responses to other incentives (e.g., food), money is thought to influence affect more consistently. This will allow us to assess specificity for our LFP biomarkers for LOC eating. A visual cue at the beginning of each trial indicates gain, loss-avoidance, or neutral (no monetary consequences) trials. After target presentation, participants receive feedback, whether they—depending on their reaction time—receive or fail to receive money on reward trials or whether they lose or successfully avoid losing money on loss-avoidance trials. To minimize learning effects, participants first complete a practice version. Subjects are also informed about the amount of money that they could earn if the task is successfully performed and cash will be shown to them. The MID task session consists of 2 runs of 72 trials each. The mean trial duration is 7.69 s, and mean inter-trial interval is 3.53 s. During the task, real-time video and LFP activity will be recorded by the RNS System and assessed offline (and in real-time) for candidate biomarkers of expected financial gains.
- Reward Questionnaire: Subjects will be asked to note, in a diary, whether they experienced any natural rewards (ex: food, water, and sex are stimuli that the brain interprets as intrinsically positive which produce pleasurable feelings, reinforcing the behavior so that it will be repeated in the future) on a daily basis that might confound the data analysis of the biomarker.
- LOC Lab Study – This behavioral laboratory assessment was designed by Stanford faculty member, W. Stewart Agras. This LOC laboratory is being used here to model the at-risk home environment of an LOC-eater in a controlled setting. We have derived this lab from our experience with Epilepsy Monitoring Units, where patients are actively weaned off their seizure medications to provoke seizures. This

allows us to monitoring electrographic activity in the brain with depth and/or surface electrodes while videoing patients simultaneously. In epilepsy, this synchronized video-electrographic data allows us to pinpoint the seizure focus and identify epileptiform activity that can be later detected by Neuropace's responsive neurostimulator, much like what we are proposing to do here for obese LOC-eaters. The time-locked video and electrographic recordings during a LOC-meal will be pivotal to characterizing the biomarker for this study. We expect this biomarker to be in the low-frequency range, but based on our recently published study, it is largely detected immediately (<2 seconds) before a LOC event. As ambulatory logging of bouts of LOC-meals may not be temporally precise, these videoed events will provide a critical piece of data that we feel will ensure success of this study and provide the foundation from which our candidate biomarker is built.

- Subjects arrive at the "eating laboratory" in the early morning (~8:00 a.m. and only one subject at a time), having fasted since midnight. Subjects remain in the lab for the entire experimental day with continuous video monitoring to assess eating (mouthfuls/min) and drinking speeds (sips/min). Caloric content will be assessed by a nutritionist. A standard breakfast is served, and lunch around noon (~1000 kcal total). In the early afternoon, a buffet is provided, containing highly caloric and palatable foods controlled across the study (e.g. glazed donuts, pizza, M&Ms, etc.) enriched by foods personalized to each individual's preference. The multi-item buffet calories will total ~5000 kcal, confirmed by our nutritionist. Subjects are told "Feel free to let yourself go and eat as much as you want." To ensure safety during this lab, patients will be interrupted from any LOC meal within 15 minutes of initiation.
- In our experience, non-bulimic binge eaters tend to take in fewer than 1500 kcal during this lab session (Telch and Agras 1996). In the Telch and Agras study, the mean kcal from the multi-item buffet consumed ranged from 1053 kcal to 1241 kcal depending on the pre-meal affect (Berg et al. 2015). Thus, there would be only a remote chance that with a 15-minute stop point that anyone would eat more than 1500 kcal. On the whole, laboratory-induced binges tend to be smaller than reported binges in the natural environment - likely restrained by the lab environment. Consultation with Dania Saarony (personal communication), MS, RD, the clinical dietician of the bariatric clinic at Stanford, revealed that the concern for patients status post bariatric surgery with LOC eating is weight regain, not any acute compromise of their metabolic syndrome or bypass. There were no other

concerns from a nutritional standpoint with this proposed protocol as outlined above. Dumping syndrome is dependent on macronutrient composition and can happen with a very low calorie intake. The amount of food patients are exposed to should have no impact on whether or not they experience dumping syndrome. Should a patient experience dumping syndrome, they will likely feel ill for a short time, but there is no long term effect of experiencing dumping syndrome. From a bariatric surgeon's perspective (personal communication with Dr. Dan Azagury), concerns regarding the gastric bypass pouch itself at 2 years after surgery are minimal, as risks of anastomic leak or staple line disruption are minimal so far out from surgery.

- The study's research coordinator will watch the video and record bites and drinks using a simple event recorder. Immediately following the buffet, when participants claim they are finished eating, or if the meal is interrupted, there will be a debriefing. Both pre- and post-buffet questionnaires will be completed. The tapes will be viewed independently by the trained research assistant and dietician, as well as the Stanford Research Group who will also be reviewing the LFP changes synchronized to the video that will be streamed in real-time from the NAc using the RNS neurostimulator and programmer.
- Pre-buffet Questionnaire. A 7-item questionnaire to assess hunger, empty stomach, fullness, and stomach discomfort. This is rated on a 9-point Likert scale.
- Post-buffet Questionnaire. This questionnaire repeats the items targeted on the pre-buffet questionnaire and includes several additional items such as (1) degree of control (or a LOC) while eating at the buffet, (2) palatability of the food, (3) classification of buffet eating episode as most like a meal, snack, binge, or overeating episode, and (4) degree of self- consciousness experienced while eating at the buffet. This is rated on a 9-point Likert Scale.

- Preoperative MRI of the brain -- A preoperative contrast enhanced volumetric MRI throughout the entire cranial volume will be obtained for surgical planning and screening.
- CT scan of the brain -- Pre- and post-operative CT scan will be obtained. Co-registration of the CT image with the preoperative MRI will allow for the assessment of lead location.
- Vital Signs – Vital signs will include Blood Pressure, Temperature, Heart Rate, and Oxygen Saturation
- Body Weight – Body weight will recorded using a calibrated scale. Participants will

be shoeless, and values will be rounded to the nearest pound and converted to kilograms.

- Laboratory Values – Blood and urine samples will be obtained and processes by the Stanford Anatomic Pathology and Clinical Laboratories. Laboratory tests include, Chemistry Panel, Hematology Panel, Lipid Protein Panel, HA1C, fasting glucose, Glucose Tolerance Testing, Serum Iron levels, Serum B12, Serum B1, Acid-base disturbances, Urinalysis, Urine Toxicology Screen
- Urine Pregnancy Test – A urine pregnancy test will be performed to determine that Women of Child-Bearing potential are not pregnant. If a urine pregnancy test results in a positive finding, a serum Hcg test will be performed.
- Bariatric Monitoring Visit – This visit should follow the Standard of Care post-surgical bariatric assessment, per the schedule of events, and additionally as needed.
- Surgical Ambulatory Unit (SAU) Pre-Operative Visit – This visit should follow the Standard of Care pre-operative visit requirements.

### 3.3.1 Pre-Screen and Informed Consent

Subjects will be vigorously pre-screened, and potentially eligible subjects identified will have a consent meeting that is videotaped and reviewed off-line by the Ethics Advisory Team to assess capacity to consent. At the informed consent meeting the informed consent form will be reviewed, all study procedures, potential benefits, alternative therapies (if available) will be clearly explained and any questions the participant may have regarding the study will be answered. Consent for release of medical records will be obtained during the meeting to acquire additional information and medical records related to study eligibility.

### 3.3.2 Pre-Operative Baseline

This visit is to occur no more than 30 days prior to the Surgery per local standards. Final confirmation of study eligibility and surgical eligibility will occur and be signed off on by multiple-Investigators after reviewing the screening visit data. A pre-operative visit will occur as Standard Health Care.

### 3.3.3 Surgical Procedure

The Stanford standard frameless stereotactic approach will be used to target the NAc. Each depth lead has 4 independently programmable cylindrical electrode contacts. Per

the targeting protocol, the distal-most contact (0) is expected to be in the ventral-most region of the NAc. Given the distance between contacts on this depth lead, contact 1 will likely be in the dorsal NAc, and contact 2 in the dorsal NAc and anterior commissure. This placement will be confirmed intraoperatively with intraoperative CT and an adjustment in electrode positioning will be made if deemed necessary by the implanting surgeon (Dr. Casey Halpern). The dorsal-most contact (3) will be in the anterior limb of the internal capsule. Sedation is administered, and the head is prepped and draped. Incisions are made, and disposable stereotactic navigation hardware is placed. The outer cannula is placed into the brain 20 mm above the target on the first side. A microelectrode is advanced stepwise, continuously recording single- and multi-unit activity for 1 min every 1 mm to physiologically define the NAc region. This is repeated contralaterally and is our standard in targeting this same region for OCD. Once the depth leads are implanted, intraoperative imaging is obtained to confirm accuracy (<2mm radial error). Leads are then secured in place, and a small right parietal craniotomy (2 x 4cm) is made for the neurostimulator. The depth leads are connected to the device, and an impedance check and real-time LFPs are performed to confirm a functioning system.

**Pulse generator replacements**

Our previous experience with RNS System indicates that the neurostimulator battery last 3-4 years, depending on the stimulation settings. As DBS for OCD tends to require frequent battery changes, we have budgeted for all patients to undergo a neurostimulator replacement if needed during the study protocol. RNS Neurostimulator replacements are routinely performed either under sedation or general anesthesia per patient preferences and are outpatient procedures. The procedure consists of reopening a small part of the scalp incision over the neurostimulator, dissection of the subcutaneous and scar tissue, disconnection of the generator from the implanted leads and connection of the new generator to these leads. Once the new neurostimulator is implanted, the layers are approximated in the routine fashion and a dressing is placed.

### 3.3.4 Monopolar Assessment and Randomization.

Immediately prior to initiating stimulation in the EFS, a monopolar stimulation assessment will be performed. Stimulation is initiated and titrated using current intensity ranges from our OCD protocol (initial parameters: 130 Hz, 90 μs, and 0.5 mA). The anticipated and desired responses are elevations in mood, and facial expressiveness.

Patients who will be blinded to test conditions are asked to report their mood, anxiety, and alertness verbally using 10-point scales. Stimulation is tested for approximately 2 min with the subjects blinded, interspersed with periods of no stimulation. Initially, the current amplitude will be set at 0.5 mA less than the lowest amplitude needed to induce a positive affective response at a single contact (to be assessed up to 4 mA).

To enhance the objectiveness of behavioral ratings and measuring side effects of stimulation initially, a randomized blinded staggered-onset design of stimulation initiation will be carried out. This strategy was adapted from prior studies of DBS for psychiatric disease, one of which Dr. Mahendra Bhati served as PI at the University of Pennsylvania – Dr. Bhati will be performing this monopolar assessment and managing the stimulation in this study (Dougherty et al. 2015; Goodman et al. 2010; Okun et al. 2013). Once the optimal settings for stimulation are determined as described above, we will wait a period of one week prior to beginning the randomized component of the study. This is intended to reduce the risk that the patient's experience during the monopolar assessment will break the blind (i.e. the patient may know what active stimulation feels like). At the planned stimulation start date, subjects will be randomized to either RNS or sham. Half of the patients will have stimulation turned on, and the other half will have stimulation initiated 2 months later such that all subjects will experience stimulation for 10-12 months. Our Maintenance of the Blind Plan will involve blinding all subjects and the study team members to the assigned grouping until the second cohort has had at least 2 months of follow-up to assess at least initial safety and placebo effects. All programming will be performed by an unblinded physician with expertise in NAc stimulation (Dr. Bhati). In the event of a serious side effect, we will unblind the involved subject.

#### 4.3.5 **Recording Phase (6 months with potential need for additional 3 months for troubleshooting)**

After implantation, subjects will enter a 6-month recording period, during which continuous, real-time NAc LFPs will be performed in the ambulatory setting using multiple modalities to target timestamped LFP analyses and in the LOC laboratory with video surveillance. Congruence of the controlled and ambulatory assessments of LOC eating and LFPs will be measured. During this phase, recording/detection parameters will be set based on prior experience with the RNS System and signals obtained in prior mouse and human studies.

Ambulatory assessments:

**Multiple 1-3 min snapshots of LFP activity will be recorded daily.**

- Recordings will be triggered by detection of our candidate LFP biomarker that we expect to refine during this recording phase based on individual data.

**Snapshots of LFP will also be stored in response to magnet swipes and at scheduled times-of-day.**

- EMA and bite-counter timestamps will also approximate times-of- day of LOC and normal meals as well as periods without eating to direct off-line LFP analyses for an indication-specific biomarker.
- Subjects magnet swipe when LOC is sensed (this is typically a sense of negative affect) (Berg et al. 2015), as is routine for EMA logging.
- Fidelity of magnet swiping will be assessed by reviewing EMA timestamps.
- The bite-counter may also provide precise timestamps of meal onsets.
- Multimodal timestamps will facilitate a targeted offline LFP analysis.

Controlled assessments:

**Laptop tasks (Milkshake Paradigm, Monetary Incentive Delay-described above) that evoke NAc signal will be used to assay NAc LFPs.**

- Real-time LFP will be recorded by the programmer for offline analyses.

**Behavioral laboratory assessment of LOC eating (validated LOC eating lab with multi- item buffet) with real-time recording of LFP.**

- Responsive stimulation will be initiated during the lab to assess safety of RNS during LOC and normal meals in a controlled setting

Biomarker identification: LFP snapshots will be recorded and downloaded daily from the device by each subject and uploaded to a secure web-based data management system as is standard for epilepsy patients treated with this device. We provide specifics here as to how an electrographic biomarker is expected to be determined.

The off-the-shelf RNS® System allows for recording from 4 independent channels of local field potential (LFP) activity sampled at 250 Hz. Bandpass filters are 2nd order (-3 dB at cutoff frequency) and are programmable; the high pass filter can be set to 0.5 Hz. The off-the-shelf RNS® System provides responsive stimulation. The

detection algorithms implemented in the neurostimulator are designed to be computationally efficient and are optimized to perform real-time LFP pattern detection. The neurostimulator will initially be configured to detect our candidate biomarker. Overtime, we will assess whether this biomarker is specific to LOC eating in humans based on multi-modality timestamps provided by each patient, and optimization in detection is expected to be needed. Clustering algorithms may be used to differentiate LFP features associated specifically with LOC eating. Identical methods will be used to identify biomarkers in the continuous real-time LFP data collected during laboratory assessments with videoed timestamps of meal onset synchronized with LFP recordings. In addition, the milkshake and monetary incentive delay tasks will be combined with tasks probing illicit drug and sex rewards to provoke a sense of anticipation that is hypothesized to drive peaks in delta power. Using these different types of rewarding stimuli is expected to improve our understanding and specificity of our candidate biomarker for LOC eating.

These behavioral assays will provide initial characterization of our biomarker such that its storage is automated in the ambulatory setting – multiple different detectors derived from these assays can be programmed simultaneously. Participants will also be asked to signify onsets of LOC by swiping a magnet over the neurostimulator to store LFPs, as is routine when seizures are sensed by epilepsy patients. Multiple studies have revealed that obese patients can reliably predict out-of-control meals. Self-reported LOC is associated with significantly high levels of premeal negative affect, and obese patients have been shown to be compliant with logging this pre-meal affect [22]. Delays in triggering recordings will complicate the analysis. To address this concern, the device will be set to allow the magnet swipe to trigger the recording of LFP activity 2 minutes prior to and 1 minute after the magnet swipe, for a total of 3 minutes. If those settings are not sufficient, the device can also be set to record up to 17 minutes after the magnet swipe. While this will not be the full resolution LFP time series data, it will still provide information about the LFP signal and inform the configuration of detectors. Patients will also be educated on the importance of swiping when the urge to LOC is sensed. An off- line LFP analysis using all timestamps will be performed to assess for the presence of peaks of delta.

Concordance with automatic detections based on our candidate biomarker will be examined. The goal will be to find electrographic features that differentiate between LFP data recorded during times associated with LOC eating (magnet LFP storage and LFP activity stored during laboratory tasks) and times not associated with LOC eating (LFP activity stored based on time-of-day). Based on results from this off-line analysis, and what is feasible with the existing firmware, the most suitable biomarker candidate will be selected for the stimulation phase of the study. If no LFP biomarker is detected, 3 months for troubleshooting have been allotted. If a biomarker is still not identified, software development will be initiated, and we have expanded the software development phase from 3 to 6 months.

Data will be analyzed by the Stanford Research group in collaboration with NeuroPace to assess LFP changes associated with LOC onsets as is routinely done for epilepsy. Identical methods will be used to identify biomarkers in the continuous real-time LFP data collected during controlled assessments. LFP analyses informed by LOC eating logs (magnet swiping and EMA) will be conducted in all stages of the clinical study. We will be able to assess LFP activity between stimulations. Over the course of the day, the total amount of stimulation is anticipated to be less than 5 minutes based on our mouse model and clinical epilepsy experience. During stimulation, the RNS settings will be adjusted to minimize sample loss during stimulation allowing for near-concurrent sense and stimulation. This capability for simultaneous sensing and stimulation may also provide a physiologic read-out for titrating stimulation parameters in the clinic using laptop-based tasks.

Programming Adjustments (3 months): If a biomarker is not initially identified during the Recording phase in the first 2 subjects, parameters will first be adjusted within the currently approved ranges with no additional software development. These adjustments may include changing the recording pathway from the standard contact-to-contact differential recording to a contact-to-can (neurostimulator housing) differential recording. This would act similar to monopolar recording since the signal from the housing is expected to be relatively flat, thus improving detections in local LFP changes.

Additionally, detector settings (line length, area or bandpass) and time-of-

day for recording can be adjusted to further increase the likelihood of identifying an LOC biomarker. Controlled assessments described above will also be repeated.

**4.3.5 Follow-up Period**

Follow-up visits will occur monthly with the contact Investigator and psychiatrist according to the schedule of events. A post-operative clinical visit in the bariatric clinic occurs at 3 months and 1 year postoperatively.

**4.3.6 Stimulation Phase**

Stimulation will be initiated in the ambulatory setting for 1-week duration, followed by a 1-week evaluation period by our study's interventional psychiatrist and PI's. If there are no serious adverse effects, stimulation will be re-initiated for 4-weeks, followed by another 1-week duration for PI review. If there are no serious adverse effects, stimulation will be re-initiated for the remainder of the 12-month assessment.

**4.4 Staggered enrollment**

Group 1: After implantation, the initial 2 subjects will enter a 6-month recording period, with continuous, real-time NAc LFPs will be performed in the ambulatory setting using multiple modalities to target time-stamped LFP analyses and in the LOC laboratory with video surveillance. Congruence of the controlled and ambulatory assessments of LOC eating and LFPs will be measured. During this phase, recording/detection parameters will be set based on prior experience with the RNS System and signals obtained in prior mouse and human studies.

During this 6 month period, an interim analysis will begin to identify the LFP candidate biomarker. If a biomarker is identified, this data will be submitted to the SMC for review. An additional 6 months of stimulation will then occur. After this period a database lock will occur, and safety data will be sent to the SMC to confirm it is feasible and safe to continue to additional subjects. This data will be submitted to the FDA prior to continuing with enrollment. Group 1 will continue to be stimulated for the 12-month period assuming there are no serious or unexpected adverse events.

Group 2: Subjects 3 and 4 will be implanted and enter a 6-month recording period with additional 6 months of stimulation. At this time a database lock will occur and interim analysis will be performed to determine it is feasible and safe to continue with enrollment. Analysis will be sent to the FDA and approval will occur prior to continuing with Group 3. Group 2 will continue to be stimulated for the 12-month period assuming there are no serious or unexpected adverse events.

Group 3: Subjects 5 and 6 will be implanted and enter a 6-month recording period and 6- month stimulation period. At this time a database lock will occur and interim analysis will occur. Safety data will be sent to the SMC to determine if it is feasible and safe to continue with enrollment. This data will be sent to the FDA and approval will occur prior to continuing to Group 4. Group 3 will continue to be stimulated for the 12-month period assuming there are no serious or unexpected adverse events.

Group 4: Subjects 7 through 10 will be implanted and enter a 6 month recording period and complete the 12 month stimulation period. A final database lock will occur after all subjects have completed the study and final analysis will occur.

## 5. ASSESSMENT OF SAFETY

### 5.1 Identification of Risks

Overall, risks associated with the RNS System include, but are not limited to, the acute risks of surgery to implant the neurostimulator and leads, risks associated with the chronic implantation of the neurostimulator and leads, risks of brain stimulation and the risk of hardware-related complications. Importantly the risks of surgical implantation of the neurostimulator and leads are well understood and do not exceed the risks associated with surgical implantation of deep brain stimulation for obsessive-compulsive disorder or movement disorders. In addition, deep brain stimulation of the NAc has been undertaken in clinical investigations for depression, obsessive compulsive disorder, alcoholism, and obesity. These investigations provide insight into the potential risks associated with delivering electrical stimulation to the NAc. The risks specific to the RNS® System, the anticipated risks of intermittently stimulating the NAc (unlike deep brain stimulation which is continuous and chronic), and the risks associated with participating in the study are described further below.

Any study subjects enrolled in this study will suffer from severe obesity that failed gastric bypass surgery and LOC eating as defined by our inclusion criteria. Specifically, subjects will be selected for this trial if they are morbidly obese with LOC eating (≥4 LOC episodes per week or 16 per month), have failed gastric bypass surgery, and meet all other inclusion-exclusion criteria. Assessment by a Stanford bariatric surgeon prior to referral to this study will occur to rule out technical explanations for suboptimal outcome with an upper gastrointestinal series within the 6 months prior to consent (i.e. a pre-study referral assessment). The upper gastrointestinal series would be evaluated by a team bariatric surgeon for 3 purposes: 1) to rule out an obstruction, stricture or gastro-gastric fistula, or other anomaly; 2) to confirm the anastomosis size ≥ 2 cm and/or gastric pouch size of ≥ 6 cm in length and ≥ 5 cm wide. If these features characteristic of technical failures cannot be well assessed on the upper gastrointestinal series, an esophagogastroduodenoscopy would be requested but this would be for clinical purposes prior to referral to this study.

Notably, there are no standard of care options for these obese patients who have failed all available therapies. Once technical failures in the bypass surgery are ruled out, and these assessments will be performed prior to and outside the context of this research study, there is little evidence from controlled trials that available adjunctive therapies (i.e., patient support groups, dietary modification, behavioral therapy, or weight loss medications) are of benefit in inducing weight loss or preventing further weight regain. Given this unmet medical need, we are proposing this investigational invasive option that may not have any benefit. Re-attempting failed therapies or introducing an available new therapy as an adjunct (e.g. weight loss medication) to what we are investigating would go beyond current practices. However, if the patient has enrolled in support groups and would like to continue this option, they could do so while participating in the trial.

<u>General Surgery:</u>
The risks associated with general surgery include but are not limited to:

- Infection at the operation site
- Blood clots, including pulmonary emboli (blood clot in your lung)
- Medication and anesthesia reactions
- Phlebitis (blood clots in the leg)
- Pneumonia (infection in the lungs)

- Atelectasis (collapsed lung)
- Soft tissue damage
- Septicemia (whole-body inflammatory state)
- Hemorrhage (excessive bleeding) possibly requiring a blood transfusion, with possible transfusion reaction
- Myocardial infarction (heart attack)
- Paralysis (part of or all of body not able to move)
- Poor tissue healing
- CVA (Stroke)
- Death

RNS® System:

The study involves at least one surgical procedure to implant the neurostimulator and leads. This surgery will be done entirely under local anesthetic. There is the possibility of complications, injury, and even death from brain surgery. The long-term effects of brain stimulation past 5 years in general are still not known and may involve risks to the patient that are unanticipated (have not been identified). In the original clinical studies with the RNS® System in medically refractory patients with partial onset epilepsy, the following side effects were experienced.

The most common side effects (experienced by more than 8 out of 100 patients) were:

- Headaches in general or after surgical procedures
- Implant site pain
- Sensations like pins and needles or burning

Less common side effects (experienced by 2 to 8 out of 100 patients) were:

- Abnormal temporary sensations
- An altered state of consciousness experienced after a seizure
- Bleeding within the brain
- Brief mild sensations that temporarily make you aware of the device (such as buzzing, clicking, vibrating, popping, prickling)
- Briefly seeing flashing lights
- Depression
- Difficulty reading, using or understanding words
- Dizziness
- Eye pain that was temporary
- Injury from a seizure
- Mild memory impairment
- Muscle twitching
- Reduced sense of touch
- Scarring, prickling, swelling, or infection of the implant site
- Surgery to fix a damaged lead

- Surgery to remove the neurostimulator or leads
- Surgery to replace the neurostimulator due to an early battery depletion

Rare side effects (experienced by less than 2 out of 100 patients) were:

- Abnormal coordination; abnormal sweating; auditory or visual hallucinations; aura before a headache; bleeding below or above the membrane around the brain; blood clot in a vein deep in the body; blurred or double vision; brain swelling; cerebrospinal fluid leak; changes in feeling in your hands or feet; changes in mood (aggression, agitation, anger, anxiety, irritability, panic attack, personality); chronic facial pain; confusion; death; decreased appetite; difficulty communicating or understanding written words; difficulty speaking or understanding words (related to epilepsy); difficulty with complex movements; discharge, skin breakdown, pain, or tissue formation at implant site; disturbance in attention; dizziness when standing; dizziness, nausea, or vomiting after surgery; excessive urination; eye or facial swelling; eyelid drooping; facial paralysis; falling; fatigue; feeling abnormal; feeling unsteady or dizzy; head deformity; head injury; hiccups; high blood pressure; inability to control emotions; infection (abscess) or complication at a stitch; involuntary eye, body, or muscle movements; irregular periods; itching; loss of hearing; loss of smell; low blood pressure when standing; medication side effects; migraine; motor speech disorder; muscle twitching; pain in the face, head or neck, hands or feet, skin, or stomach; problems hearing; problems walking; problems with healing; problems with the neurostimulator battery; psychosis after seizure; receding gums; ringing in the ears; sensitivity to light; sinus pain; sleepwalking; status epilepticus (convulsive or nonconvulsive); stuttering or stammering; suicidal depression; surgery to change a lead; surgery to fix an opening in the skull; swelling in the legs or feet; temporary (transient) blindness; thinking about hurting or killing yourself; tongue biting; trying to kill yourself; water on the brain; weakness in a limb or on one side of the body; whirling or spinning feeling.

Risks to pregnant women, embryos, fetuses

- The safety and effectiveness of the RNS® System has not been studied in pregnant women. It is unknown whether there are any risks to pregnant women, embryos, fetuses or breastfeeding infants. Therefore, women who are pregnant cannot enroll in the study.

Medical environment warnings.

- Lithotripsy, radiation, electrolysis, CT scans, and TMS have not been studied with the RNS System. Exposure may damage the RNS System. This could result in loss of therapy, and additional surgery to remove or replace components of the RNS System.

Stimulation of NAc: There is also the potential for disordered eating related risks that could be affected by treatment with the RNS System. These include the potential for stimulation to cause new or worsened symptoms, and to increase the risk for development or exacerbation of psychiatric symptoms. Studies in humans of chronically stimulating the NAc for obsessive compulsive disorder and depression have demonstrated largely efficacious results with tolerable side effects and a low rate of complications. Potential complications of this surgery are expected to mimic the surgery we propose although the pattern of stimulation (intermittent vs. chronic) will be different and the overall cumulative "dose" of stimulation much less. Complications can be separated into those related to surgical implantation, stimulation, and device failure or interruption. No device failures beyond the expected stimulation interruptions, owing to implantable neurostimulator battery depletion are anticipated. Effects of stimulation may be seen during intraoperative testing or subsequent optimization, in which a wide range of settings are surveyed that in some instances may lead to unwanted or unusual emotional, perceptual, or somatic experiences. All these effects are expected to occur within seconds or minutes of stimulation onset, and can be reversed, typically within seconds and always within minutes of changing the stimulation parameters. Transient emotional effects may include euphoria, giddiness, anxiety, panic attacks, or sadness. Contralateral smiles have been induced intraoperatively in patients, and ventral stimulation is more likely to induce anxiety/panic. Transient olfactory and gustatory sensations such as smelling popcorn or tasting metal have been reported with deep ventral contacts only. Hypomania has been observed at some point during chronic NAc stimulation in obsessive-compulsive disorder patients. In all cases, the degree of mood elevation abated over time or responded to device adjustment. Difficulty falling asleep is a common complaint that can be improved by device adjustment. Serious adverse events have been found to be rare, though if required should be managed by a change in the device settings. Any significant changes in device settings will lead to longer in-clinic observational periods (up to 1 hour).

Neuropsychological Battery, Psychological Assessments, and Patient Reported Outcomes

The completion of the psychological assessments and questionnaires may cause emotional stress or anxiety. The subject will be provided breaks as necessary and can refuse to complete any assessment that does not assess safety or

suicidality.

### 5.1.1 Mitigation of Risks

One mechanism to mitigate risk of stimulation is to slowly increase the amplitude in 0.5 mA increments under direct observation. With RNS, once stimulation is initiated, bursts are brief (e.g. 100ms - 10 seconds), and thus, any detectable side effects will likely be brief as well. Patients will be monitored in clinic until side effects have disappeared such that adjustments can be made prior to the patient subjects departing from the clinic. As is routine for programming in epilepsy, we will test new stimulation settings during office visits to ensure that subjects have no clinical symptoms or after-discharges on real-time local field potential (LFP) recordings. In addition to weekly follow-up (or more if needed), subjects will log any noticeable side effects using handheld wireless ecological momentary assessment (EMA), the time-stamped data of which is accessible remotely to the study team. Subjects will be instructed to return to clinic immediately if experiencing a concerning side effect such as hypomania or acute depression. In the case of an emergency, subjects and their health care proxy will be instructed on how to stop stimulation if directed by our interventional psychiatrist. Stimulation (and detection) settings will be adjusted initially at weekly clinic visits based on clinical history, EMA logs, our modified eating disorder battery (see below), stored detections, and side effects.

As these patients will be obese (BMI as high as 60 kg/m$^2$), it is reasonable to be concerned with procedural risks. These will be mitigated by doing the surgery awake with the head of bed at 30 degrees by the guidance of our bariatric colleagues, and local anesthetic and monitored anesthesia care will be provided. This is our typical procedure for DBS surgery in movement disorders and OCD, so we have a very experienced intraoperative team. Given there is no tunneling as is routine for DBS, we feel patients will tolerate this awake surgery well.

Additionally, the RNS System was designed to use standard surgical techniques to implant the neurostimulator and leads, including craniectomy and stereotactic placement of depth leads. The implantable portions of the RNS System employ known implantable materials generally considered to be biocompatible and safe for permanent implant use. In addition, the neurostimulator is designed to turn off if

over-stimulation or excess current occurs or the device is not functioning properly.

Patients will be given a patient manual including, but not limited to, information regarding contraindications, concerns regarding medical environments, and potential risks of the RNS System. In addition, following surgery patients will be provided a medical implant ID card. The intent of a medical implant ID card is to inform individuals (e.g. medical provider, TSA, caregiver) about the implanted product and provide contact information if further detail is needed.

In order to mitigate additional risks due to procedures or the surgery, stopping rules will be followed as noted in Section 5.3.2.

The study sponsor and programming psychiatrist will work with the patient, their family, and collaborating investigators to ensure there is open communication about any adverse events from the time of enrollment and surgery to the perioperative period and programming visits. The study sponsor who will be the implanting surgeon, will also be present for all follow-up programming sessions after surgery. This continuity of care will be essential to ensure point of contact is clear to all patients and family members. If needed, the patient will be encouraged to come to the clinic more frequently than every month – and this will be required per our protocol during initial 6 weeks of programming – if any new symptoms or adverse effects are reported. We will give patients and family instructions on possible adverse effects they may see after leaving the clinic (summarized below), so they should know what to identify and contact us immediately. We will also ask for patients to call the clinic should any question arise about their surgical recovery or response to stimulation.

The study sponsor's clinic will be the point of contact for all patients' needs, and an After Visit Summary will be provided to each patient after each visit as is standard protocol for clinical care at Stanford Health Care. Notably, acute side effects during programming are expected to be seen, and patients will remain in the clinic for 30 minutes after any programming adjustment to ensure no delayed effects occur. However, patients and their family will be educated on possible delayed effects of stimulation, which based on our experience from similar treatment in a different patient population would most commonly be the following:

1) Mania
2) Depression
3) Suicidality

While unexpected but more relevant to this patient population, the following side effects may also be noticed by patients or their family, for which they will be asked to call the clinic immediately:

4) Anorexia
5) Worsened Loss of Control over Eating

While the patient indication and brain targets are different, these are the most common side effects in epilepsy patients treated with responsive stimulation:

6) Headache
7) Dysesthesia/Paresthesia
8) Photopsia
9) Cognitive changes
10) Muscle twitching
11) Dizziness

A typical After Visit Summary for a neurosurgical visit is copied here and will be adapted for these trial patients with these side effects above:

## AFTER VISIT SUMMARY

## Instructions

### Need Help?

Notify MD

If you develop symptoms listed below, please seek medical attention:

- new weakness or numbness
- severe headache
- new seizure
- severe nausea/vomiting
- fever > 101.5
- headache with neck stiffness and fatigue
- increasing redness around or discharge from incisions

If your symptoms are concerning, but you are not sure if you should come to the hospital, you may call the neuroscience clinic at 650-723-6469. After hours, you may call 650-723-6661 and ask to speak with the neurosurgery resident on call.

If your symptoms are more severe, you are concerned you may be having a stroke, or if you have chest pain or difficulty breathing, please call 911 or go to the emergency room.

Staggered enrollment will also be incorporated to reduce the risk of implanting additional subjects should a LFT not be identified or AEs be noted.

If a woman is pregnant at the time of screening, she will not participate in this study. To confirm to the extent medically possible that a woman is not pregnant, she will agree to have a pregnancy test done before beginning this research study. She must agree to avoid sexual intercourse or use two methods of birth control method judged to be effective by the investigator and which will not interfere with the proposed investigation. She must accept the risk that pregnancy could still result despite the responsible use of reliable method of birth control. She

must agree to notify the investigator as soon as possible of any failure of proper use of her birth control methods, or if she becomes pregnant.

For women of childbearing age who have not had definitive surgical sterilization such as hysterectomy and/or oophorectomy, it is expected that she will use two effective methods of birth control to prevent exposing a fetus to any potential risk during the course of the study. If the woman is pregnant or currently breast feeding, she may not participate in this study. The woman must understand that if she is pregnant, if she becomes pregnant, or if she is breast-feeding during this study, she or her child may be exposed to potential risks. Thus, without evidence that robustly supports that RNS of the NAC will not harm a developing fetus, if a study subject becomes pregnant, stimulation will be stopped for the duration of the pregnancy.

<u>CT Scans, Electrolysis, Lithotripsy, and Radiation</u>

In addition to the outlined information below, whenever a CT scan is obtained, we will ask patients to do so at Stanford Health Care when possible, so that an in-clinic real-time recording and a programmer-commanded test stimulation can be tested to ensure that the device is functioning properly. If a CT scan is needed for any emergent reason and is obtained at an outside facility, we will coordinate a clinic or in-hospital visit as soon as possible with the patient to do a test stimulation for verification purposes.

<u>COMPUTERIZED TOMOGRAPHY (CT) SCANS</u>

For CT procedures on a patient with an implanted RNS® Neurostimulator, the operator should:

- Ask the patient to have the neurostimulator temporarily shut off with a programmer while the scan is performed, if possible.
- Minimize x-ray exposure to the implanted electronic medical device by:
  - Using the lowest possible x-ray tube current consistent with obtaining the required image quality.
  - Making sure that the x-ray beam does not dwell over the device for more than a few seconds.

<u>After CT scanning, the operator should:</u>

- Ask the patient to have the neurostimulator turned back on with a programmer.
- Advise the patient to contact their healthcare provider as soon as possible if

they have questions or suspect their device is not functioning properly after any medical procedure.

Regarding electrolysis, lithotripsy, and radiation:

ELECTROLYSIS

The effects of electrolysis on the RNS® System have not been studied. Electrolysis on the head or neck should be avoided in this study.

LITHOTRIPSY

The effects of extracorporeal shock wave lithotripsy on the RNS® System have not been studied. Exposure to high-output ultrasonic frequencies may damage the RNS® System. This could result in loss of therapy, and additional surgery to remove or replace components of the RNS® System. Patients will likely have to be explanted and removed from this study should lithotripsy become necessary during the trial.

RADIATION

The effects of high radiation sources (such as cobalt 60 or gamma radiation used in cancer therapy) on the RNS® System have not been studied. Exposure to high levels of radiation may damage the RNS® System. This could result in loss of therapy, and additional surgery to remove or replace components of the RNS® System. Patients will likely have to be explanted and removed from this study should radiation therapy become necessary during the trial.

### 5.1.2 Potential Benefit and Alternatives

The potential benefit to participating in the study is the attenuation of disordered eating behaviors. This could lead to improved quality of life, health and weight loss. However, the likelihood of attaining these benefits is unknown. If successful, this study could lead to the further development of an alternative treatment option for obese patients and even non-obese patients who suffer from compulsive eating. Overall, the purpose of this trial is to assess feasibility and safety of the intervention.

Participation in this study is not required to receive treatment for obesity with binge- eating-like disorders. Some example alternative treatments available to the human subjects including:

i. Continuation of current medical and/or behavioral treatment
ii. Support groups
ii. Other surgical procedures:
- Gastric band
- Gastric sleeve
- Gastric bypass surgery

**Our inclusion criteria require that all of these treatment alternatives have been Attempted (with the exception of support groups).**

## 5.2 SAFETY PARAMETERS AND DEFINITIONS

### 5.2.1 Adverse Events

An Adverse Event (AE) is any untoward medical occurrence in a clinical investigation subject implanted with a study device, regardless of causal attribution. An adverse event can therefore be any of the following:

- Any unfavorable and unintended sign (including an abnormal laboratory finding), symptom, or disease temporally associated with the use of the investigational device
- Any new disease or exacerbation of an existing disease (a worsening in the character, frequency, or severity of a known condition)
- Adverse events that are related to a protocol-mandated intervention, including those that occur prior to assignment of study treatment

Any AE that occurs during the subject's participation in the study will be recorded on an AE case report form. All AEs will be followed until the event is resolved or considered stable. AEs will be reported to the IRB as required by local policy.

Adverse of Special Interest (AESI) include device malfunction resulting in the removal or revision of the device, including but not limited to lead replacement, or infection as a result of the device placement. Any AESI recorded will require immediate hold of the trial until adjudicated by the SMC.

#### 5.2.1.1 Assessment of Severity of AE

The Investigator will characterize the severity of each AE as mild, moderate, or severe. The assessment is subject and the Investigator will use medical judgment to compare the reported AE to similar observed events in the clinical practice.

Guidance for assessing AE severity is located in TABLE 9.3.

| Severity | Description |
|---|---|
| Mild | Discomfort noticed, but no disruption in normal daily activities |
| Moderate | Discomfort sufficient to reduce or affect normal daily activities |
| Severe | Incapacitating or considerable interference in ability to perform normal daily activities |

**5.2.1.2 Assessment of Causality of AE**

The relationship of all AEs to the device or the surgery will be classified by the Investigator as not related, possibly related, probably related, or definitely related.

Not related: The AE is due to an underlying or concurrent illness or effect of another device, drug or intervention and is not related to the study device or surgery.

Possibly related: The causal and/or temporal relationship to the study device or surgery, is equally or less likely than other plausible explanations.

Probably related: The causal and/or temporal relationship to the study device or surgery, is likely or significantly more likely than other plausible explanations.

Definitely related: A clinical event that can only be attributed to the device or the surgery.

**5.2.1.3 Abnormal Lab Values**

Throughout the course of the trial, any abnormal lab values, as assessed by the Stanford Anatomic Pathology and Clinical Laboratories, will be defined as clinically significant or not clinically significant by the Investigator. All clinically significant laboratory values will be recorded as adverse events.

**5.2.1.4 Abnormal Vital Sign Values**

Vital signs will be reviewed by the Investigator throughout the course of the trial for abnormality. Abnormal weight will not be considered as this is expected for this particular patient population, however the following criteria will determine if a vital sign is considered abnormal:

Blood Pressure: Any Systolic measurement > 180 mmHg or < 100 mmHg, or any Diastolic measurement >110 mmHG or < 40 mmHg

Heart Rate: Any heart rate measurement >100 bbp or <50 bbp

Temperature: Any oral temperature >38.5 C or < 36.0 C
Oxygen Saturation: Any measurement that is a respiratory rate of < 93%.

All abnormal vital sign values will be assessed as clinically significant or not clinically significant and recorded as an AE if considered clinically significant.

#### 5.2.2 Serious Adverse Event

Any AE that results in one or more of the following is considered a Serious Adverse Event (SAE): death, life threatening situation, inpatient hospitalization, persistent or significant disability/incapacity, and other medically important events. Definitions of SAEs are:

- Death: the subject dies during participation in the study.
- Life threatening situation: the subject is at risk of death at the time of the event, but does not refer to the hypothetical risk of death if the AE were more severe or were to progress.
- Inpatient hospitalization: a subject requires hospitalization or prolongation of an existing hospitalization, including medical or surgical intervention to prevent permanent impairment to a body structure or body function during participation in a clinical study.
- Persistent or significant disability/incapacity: any AE having an outcome that is associated with a substantial disruption of the ability to carry out normal life functions. This includes the inability to work, but is not intended to include transient interruptions of daily activities.
- Other medically important events: important medical events that may not result in death, be life-threatening, or require hospitalization (including emergency room visits) but may be considered a serious AE when, based upon medical judgment, they may jeopardize the subject.

All SAEs will be reported to the SMC for review and adjudication to determine association with the treatment procedure and/or implanted device system. The Investigator must confirm the SAE in appropriate source documentation and provide detailed information pertaining to the event if required. Additionally all SAEs will be reported to the IRB as required by local policy.

### 5.2.3 Unanticipated Adverse Device Effect (UADE)

An Unanticipated Adverse Device Effect (UADE) is defined as any serious adverse effect on health or safety or any life-threatening problem or death caused by, or associated with, a device, if that effect, problem, or death was not previously identified in nature, severity, or degree of incidence in the investigational plan or application (including a supplementary plan or application), or any other unanticipated serious problem associated with a device that relates to the rights, safety, or welfare of subjects.

The Investigator is required to submit a report of a UADE to the FDA and IRB as soon as possible, but no later than 10 working days after first learning of the event.

## 5.3 MONITORING AND OVERSIGHT

Stanford will perform internal monitoring to ensure the protocol is followed. In-services will be conducted to review pertinent information about and train on the requirements of the protocol. The contact PI will sign off on all training, and all training records will be maintained in the regulatory binder. This must occur before subject screening or receipt of devices. Internal monitoring will verify 100% of data entered into the EDC system and other study site documentation will be evaluated for completeness and accuracy against source documents. After a subject is enrolled, monitoring activities will confirm the subject consenting and screening procedures for GCP and adherence to the protocol requirements. All information on the eCFRs must correlate to the source documents in the subject's file. Throughout all monitoring, Stanford will evaluate protocol compliance and adherence to regulations, and report to the appropriate authorities as necessary.

### 5.3.1 Safety Monitoring Committee

A Safety Monitoring Committee (SMC) composed of independent arbitrators with expertise in the surgery, obesity, and eating disorders. The SMC will be assembled to monitor the safety of the study and to make recommendations about safeguarding the interests of subjects regarding stopping, modifying or continuing the study. Study data are made available to the SMC. The SMC should oversee the effectiveness data at after 6-months of stimulation in each subject to determine if there's enough evidence of efficacy to justify the risk associated with the device and procedure. These outcome data will also be shared with the FDA prior to

proceeding with the next phase of the study.

The SMC will also assess the ongoing validity of the study. A minimum of 1 meeting is scheduled every month after the first implant with ad hoc meetings as required depending on the progress of the study. The type of data typically monitored may also include any of the following: demographic variables including comorbid conditions, number of patients screened and exclusion reasons, recruitment data, behavioral and electrographic data quality, assessment of dropouts with reasons for each, treatment visit compliance, hospitalizations for any reason, all adverse events including study withdrawals. Recommendations regarding the study are deliberated in closed sessions. These data will be provided to the SMC prior to each meeting to allow time for review. At each meeting the SMC will first review and discuss any aspect of the trial data and its overall progress, followed by a vote as to whether or not the trial should continue. The SMC will then discuss any problems with the trial and methods to resolve them. As designated by the SMC, if any surgical or stimulation-related side effects lead to adverse events and are of serious concern (i.e. hospital admission, irreversible or life- threatening), study will be halted for SMC review. The minutes will be forwarded to the PIs.

#### 5.3.2 Patient, Treatment, and Study Discontinuation

Patient Discontinuation: Prior to surgery, a subject will be discontinued in the trial if they meet any of the exclusion criteria or are less than 80% compliant with the ecological momentary assessment (EMA) prior to surgery. After surgery, a patient can be discontinued if the Investigator feels continuation would be harmful or if they are not compliant with attending visits. Moreover, patients must remain 80% compliant at least 1 week per month with the EMA, as this is the primary efficacy outcome.

Treatment Discontinuation: Recording and Stimulation will cease if an adverse event is irreversible, life threatening, or not tolerable after reprogramming. The subject will continue to be followed for safety assessments. Should the Investigator feel that the device needs removal, another surgery will occur to remove the device.

Study Discontinuation: A stopping rule will be put in place should a serious effect, as adjudicated by the SMC, occur in one subject (i.e. hospital admission, irreversible or life- threatening) or if one subject experiences any unexpected adverse events related to surgery, the device, or stimulation as determined by the SMC.

#### 5.3.3 Psychiatric Conditions

All monitoring in this study during the stimulation phase will be performed by the study team, including the sponsor (neurosurgeon) and interventional psychiatrist. Following a 6-month recording phase, stimulation will be initiated in the clinic setting during a standard monopolar stimulation assessment with parameters informed by our prior experience in psychiatric diseases specific to the nucleus accumbens. Patients will be blinded to test conditions and asked to report mood, anxiety, and alertness verbally using 10-point scales. Anticipated side effects are well-known and abate over time or quickly respond to parameter adjustment. Patients will be asked to remain in the clinic for 30 minutes to ensure symptom stability. They and their family will also be informed of the common delayed side effects (listed above) of stimulating the accumbens that may occur once they leave the clinic. They will be asked to call the study sponsor's clinic should these effects develop. Patients and their families will also be told to call the study sponsor's clinic with any questions about their recovery or symptoms they may be having, even if they are not related to the stimulation.

Should any psychological adverse events be reported to the study sponsor's clinic, patients will be immediately seen at the same time by the study's sponsor and interventional psychiatrist. A programming adjustment will be performed, which is expected to ameliorate the side effect. For the most part, side effects are expected during programming, and adjusting the parameters is expected to ameliorate the effect. Delayed, relatively minor side effects (i.e. not requiring hospitalization and not acutely life-threatening), such as mood changes, suicidal ideation, anorexia, worsened loss of control over eating, and common side effects of RNS (listed above) will be addressed in the sponsor's clinic with programming readjustment. Should a patient or family member call in with a major adverse event deemed serious by the study sponsor such as acute suicidality with a plan (i.e. requiring hospitalization and acutely life-threatening), we will advise the patient to call 911

and hospitalize the patient. The programming plan would be to shut off the device and observe the patient for about 30 minutes. We would then restore the settings at a lower current amplitude.

In order to avoid stimulation-induced side effects, current amplitude will be set at 0.5 mA less than the lowest amplitude needed to induce a positive affective response at a single contact (to be assessed up to 4 mA). In another attempt to ensure safety, prior to initiating stimulation in the ambulatory setting, a multi-item buffet validated as a means to provoke loss of control eating will be used to assess effects of initial stimulation parameters in a setting that is designed to mimic an at-risk environment. Stimulation will only be initiated if no adverse effects are seen and then the randomized evaluation phase (described below) in the ambulatory setting will begin. If adverse effects are seen during the lab, patients will return to clinic for reassessment of monopolar stimulation at all contacts and readjustment. In addition to clinical observations of side effects and weekly-monthly visits with the contact PI and the study's interventional psychiatrist, we also plan for bariatric, neuropsychology, and nutrition visits throughout the study.

To enhance the objectiveness of behavioral ratings and measuring side effects of stimulation initially, we propose a randomized blinded staggered-onset design adapted from prior studies of DBS for psychiatric disease. At the planned stimulation start date, subjects will be randomized to either RNS or sham. Half of the patients will have stimulation turned on, and the other half will have stimulation initiated 2 months later. Our Maintenance of the Blind Plan will involve blinding all subjects and the study team members to the assigned grouping until the second cohort has had at least 2 months of follow-up to assess at least initial safety and placebo effects. All programming will be performed by an unblinded physician with expertise in NAc stimulation. In the event of a serious side effect (i.e. hospital admission, irreversible or life-threatening), we will unblind the involved subject.

Stimulation will first be initiated in the ambulatory setting for 1-week duration, followed by a 1-week evaluation period by our study's interventional psychiatrist and PI's. If there are no serious adverse effects, stimulation will be re-initiated for 4-weeks, followed by another 1-week duration. If there are no serious adverse effects, stimulation will be re-initiated for the duration of the assessment. Safety

assessments include treatment-emergent adverse event documentation, weekly-monthly monitoring visits, psychiatric assessments, the Columbia–Suicide Severity Rating Scale, vital signs, body weight, labs, and overall nutrition throughout the study. Weight is recorded using a calibrated scale in shoeless participants, rounded to the nearest 0.5 pounds, and converted to kilograms. Telemetry and CPAP will be available as needed for all subjects in house. In addition to weekly-monthly visits with the contact PI and psychiatrist, a postoperative clinical visit in the bariatric clinic is planned at 3 months and 1 year postoperatively. Labs and an electrocardiogram will be obtained, and vital signs, urinalysis, and serum levels of iron, B12, B1, calcium, acid-base disturbances, amylase, glucose tolerance testing, and a lipid panel will be evaluated. We budgeted for 3 additional clinical evaluations during the stimulation phases to support any untoward side effects, as well as neuropsychology and nutrition visits for unanticipated effects, including worsening of LOC or even overly restrictive eating consistent with post-surgical eating avoidance disorder. Routine eating disorder assessments will ensure these are detected early.

### 5.4 Protocol Deviations

A protocol deviation is any noncompliance with the clinical trial protocol, International Conference on Harmonisation Good Clinical Practice (ICH GCP), or Manual of Procedures (MOP) requirements. The noncompliance may be either on the part of the participant, the investigator, or the study site staff. As a result of deviations, corrective actions are to be developed by the site and implemented promptly.

The practices are consistent with ICH GCP:

- 4.5 Compliance with Protocol, sections 4.5.1, 4.5.2, and 4.5.3
- 5.1 Quality Assurance and Quality Control, section 5.1.1
- 5.20 Noncompliance, sections 5.20.1, and 5.20.2

## 6. STATISTICAL CONSIDERATION AND ANALYSIS PLAN

### 6.1 Sample Size

The study cohort size is small, only 10 patients with intent to treat. Outcome measures will be included as described above, but this is not a controlled trial, limiting statistical comparisons. Results, per patient, will be described using

standard summary statistics, including evaluating outcomes at each visit and assessing changes from baseline.
Summary statistics will also be generated for results across the 10 subjects. The analysis will be primarily performed on a per patient basis.

### 6.2 Data Analysis

This study will be used to inform future power calculations for a formal evaluation of safety and efficacy, but the primary intention here is feasibility and non-futility testing, as no prior data has ever been collected on the use of this device for this indication.
Descriptive statistics will be provided to evaluate changes within subject for LOC and mood assessments taken before and after an eating episode. Results obtained from multimodal ambulatory assessments of LOC episodes will be assessed for congruence and evaluated graphically. Spearman and Pearson correlation coefficients will be calculated. Feasibility of calculating the performance of LFPs as predictors of LOC episodes will be examined using each of these 3 separate approaches. Diagnostic accuracy will be calculated for each method. Additionally, safety will be assessed based on AEs, SAEs, and UADEs.

## 7. DATA COLLECTION AND MANAGEMENT

### 7.1 Data Quality Assurance

The Investigator will be responsible for data management of this study, including quality checking of the data. The Investigator will designate Stanford representatives to be responsible for managing, monitoring, coordinating and performing data analyses for the study. The data collected by the RNS System will be sent to a secure server at NeuroPace, Inc, as is done routinely for epilepsy patients who are currently treated clinically with this FDA-approved device. The data will be made available for visual inspection by the researchers at Stanford identified in the IRB protocol and informed consent via the NeuroPace Patient Data Management System. In addition, the raw LFP files will be shared with the researchers at Stanford for offline analyses. Scientists at both Stanford and NeuroPace will be involved in the offline analyses of the LFP data.
Appropriate legal agreements will be drafted to ensure HIPAA compliance and patient consent to data sharing. Only HIPAA-certified users will be authorized to

access identifiable patient data. A web-based data sharing system will be developed by NeuroPace to provide access to clinical data collected under the project. This system will allow authorized access and downloading of device data. Data will be indexed, cataloged, identified and de-identified as necessary. Data will be updated at least weekly. Additionally, a set of software tools for importing the data into analysis programs will be provided.

Ongoing source data verification will occur to confirm that critical protocol data entered into the eCRF by authorized site personnel are accurate, complete, and verifiable.

**7.2 Electronic Case Report Forms**

The majority of data collected during the study will be captured using a 21 CFR Part 11 compliant Electronic Data Capture (EDC) system, which will be appropriately tested to ensure compliance with study requirements. The research team is responsible for entering the required data into the data collection forms on the EDC system in a timely manner. Data collected during the study are captured using this EDC system. Original observations (e.g. medical history) are those values that represent the first recording of study data gathered during clinic appointments. Original observations documented on data collection forms are the official record (e.g. source).Source documents are required to verify the accuracy of the data submitted. Data revisions are captured within the EDC system, which provides a complete audit trail. This audit trail includes by whom the data was entered, the date of time the entry was made, and if data is changed, the trail will not obscure the original entry but show what it was changed from and to.

Database locks will occur prior to each interim report throughout the trial. The database lock will occur after 6 months of stimulation for each staggered enrollment group. Only data that is entered prior to a database lock will be reviewed by the DMC for the interim analysis.

**7.3 Source Document Data**

Ongoing source data verification will occur to confirm that critical protocol data entered into the EDC by authorized site personnel are accurate, complete, and verifiable. Source documents that are required to verify the validity and

completeness of data entered into the EDC must not be obliterated or destroyed and should be retained per the requirements noted in Section 7.6.

### 7.4 Confidentiality

The Investigator maintains confidentiality standards by coding each patient enrolled in the study through assignment of a unique patient identification number. Protected Health Information obtained by this study is confidential and may be disclosed to third parties only as permitted by the Informed Consent Form signed by the patient, unless permitted or required by law.

Per IRB policy, any protected health information and confidential information obtained for pre-screening will be appropriately discarded per Stanford policy. Additionally, a Waiver of HIPAA Authorization will be IRB approved to allow for the collection of the PHI in prescreening.

### 7.5 Resource and Data Sharing Policy

The proposed experiments will produce substantial data regarding the efficacy and safety of neurostimulation in an animal model of maladaptive eating behavior and from clinical studies of disordered eating in refractory obese patients. In addition, these studies will provide analyses of electrographic activity during disordered feeding behavior and in response to high calorie food cues. To advance the goal of widespread data sharing among researchers, data generated from this study will be shared via the National Database for Clinical Trials (NDCT). Enrollment and consent procedures will incorporate requirements to meet this objective. The budget also contains necessary funding to support the costs of data submission, including effort needed by the PIs and data center staff. The applicants further certify that all data will be reviewed for accuracy and submitted to the NDCT semi-annually (January 15 and July 15) and that submission of all other data will be made at the time of publication of the main outcome results or prior to the end of the grant, whichever comes first. All results, whether positive or negative, specific to the cohorts and outcome measures will be submitted. Data generated by this project will be presented annually at national meetings (e.g. American Association of Neurological Surgeons, The Obesity Society) and the results from the completed non-clinical experiments and the clinical study will also be shared via peer- reviewed publications.

### 7.6 Compliance with Laws and Regulations

This study will be conducted in full conformance with the ICH E6 guideline for Good Clinical Practice and the principles of the Declaration of Helsinki, as well as with FDA regulations and the local IRB.

Before initiating the trial, the investigator will have written and dated approval from the IRB for the trial protocol, written informed consent form, consent form updates, subject recruitment procedures (e.g., advertisements), and any other written information to be provided to subjects. Additionally, the Investigator is responsible for providing written summaries of the status of the study to the IRB annually or more frequently in accordance with the requirements, policies, -and procedures established by the IRB.

The investigator will not implement any deviation from, or changes of, the protocol without prior review and documented approval from the IRB of an amendment, except where necessary to eliminate an immediate hazard(s) to trial subjects.

### 7.7 Conflict of Interest Policy

The objectivity of this study from any actual of perceived influence, such as NeuroPace Inc., is critical. Therefore, any actual conflict of interest (COI) of persons who have a role in the design, conduct, analysis, publication, or any aspect of the trial will be disclosed and managed. If any study personnel have a perceived COI, it will be managed appropriately in accordance with their participation in the design and conduct of this study. All COIs will be managed according to all federal and local regulations.

The Stanford IRB requires additional language relating to any potential conflict of interest by the investigators involved with the protocol or the institution. After the relevant conflict of interest review process, the IRB requires a clear statement in the consent document concerning any conflict of interest that has not been eliminated when such investigator is allowed to continue. Potential conflicts of interest and the review process are addressed in detail in HRPP Chapters 3 and 14.

## 7.8 Informed Consent Process

The informed consent process will follow the Stanford University Human Research Protection Program (HRPP) Guidance based on the legally required Common Rule 45 CFR 46.116(a) and FDA Regulations 21 CFR 50.25(a).

Informed consent if a process that is initiated prior to an individual's agreeing to participate in the study and continues throughout the individual's study participation especially if there are changes to the safety profile of the study procedures and/or device, The informed consent information must include the following: 1. A statement that the study involves research, 2. An explanation of the purposes of the research, 3. An explanation of the expected duration of participant's participation, 4. A description of what procedures will be followed, and 5. Identification of any procedures that are experimental.

The informed consent will be approved by the Stanford Institutional Review Board (IRB) before use by the key personnel. Only key personnel designated the role to consent research participants on the Delegation of Authority (DOA) can participate in the consenting process. The research participant will be asked to review the document and the investigator will explain the research study to the participant and answer any questions that may arise. Participants will have the opportunity to carefully review the written consent form and ask questions prior to signing. The participants should have the opportunity to discuss the study with their family and/or surrogates or take time to consider participating prior to signing the consent document. It is crucial that participants must be informed that participation in voluntary and that they may withdraw from the study at any time without penalty and without any consequences to their medical care.

A copy of the consent document will be given to the research participant for their records. The informed consent process will be conducted and documented in the source document, including the date and signature of the participant and the investigator or the key personnel conducting the informed consent process.

### 7.9 Retention of Records

Per FDA guidelines, the investigator shall retain the study records for a period of 2 years following the date a marketing application is approved for the device for the indication for which it is being investigated; or, if no application is to be filed or if the application is not approved for such indication, until 2 years after the investigation is discontinued and FDA is notified. The study records include but are not limited to study data, regulatory documents such as IRB approval letters and FDA correspondence, and informed consent documents. In order to ensure these records can be easily access in the case of an audit, a record archiving facility will be used to house all study records once they leave the Stanford facility.

## 8. REFERENCES

**Bibliography** 1. Ardestani A, Rhoads D, Tavakkoli A: Insulin cessation and diabetes remission after bariatric surgery in adults with insulin-treated type 2 diabetes. **Diabetes Care 38:**659-664, 2015

2. Beaver JD, Lawrence AD, van Ditzhuijzen J, Davis MH, Woods A, Calder AJ: Individual differences in reward drive predict neural responses to images of food. **J Neurosci 26:**5160-5166, 2006

3. Benton AL, Hamsher K (eds): **Multilingual Aphasia Examination, ed 2nd Edition.** Iowa City: AJA Associates, 1989

4. Bergey GK, Morrell MJ, Mizrahi EM, Goldman A, King-Stephens D, Nair D, Srinivasan S, Jobst B, Gross RE, Shields DC, Barkley G, Salanova V, Olejniczak P, Cole A, Cash SS, Noe K, Wharen R, Worrell G, Murro AM, Edwards J, Duchowny M, Spencer D, Smith M, Geller E, Gwinn R, Skidmore C, Eisenschenk S, Berg M, Heck C, Van Ness P, Fountain N, Rutecki P, Massey A, O'Donovan C, Labar D, Duckrow RB, Hirsch LJ, Courtney T, Sun FT, Seale CG: Long-term treatment with responsive brain stimulation in adults with refractory partial seizures. **Neurology 84:**810-817, 2015 PMC4339127

5. Bewernick BH, Hurlemann R, Matusch A, Kayser S, Grubert C, Hadrysiewicz B, Axmacher N, Lemke M, Cooper-Mahkorn D, Cohen MX, Brockmann H, Lenartz D, Sturm V, Schlaepfer TE: Nucleus accumbens deep brain stimulation decreases ratings of depression and anxiety in treatment-resistant depression. **Biol Psychiatry 67:**110-116, 2010

6. Bohon C: Greater emotional eating scores associated with reduced frontolimbic activation to palatable taste in adolescents. **Obesity (Silver Spring) 22:**1814-1820, 2014 PMC4115016

7. Bohon C, Stice E, Spoor S: Female emotional eaters show abnormalities in consummatory and anticipatory food reward: a functional magnetic resonance imaging study. **Int J Eat Disord 42:**210-221, 2009 PMC2739233

8. Bray GA, Tartaglia LA: Medicinal strategies in the treatment of obesity. **Nature 404:**672-677, 2000

9. Broft A, Shingleton R, Kaufman J, Liu F, Kumar D, Slifstein M, Abi-Dargham A, Schebendach J, Van Heertum R, Attia E, Martinez D, Walsh BT 10. Bronte-Stewart H, Louie S, Batya S, Henderson JM: Clinical motor outcome of bilateral subthalamic nucleus deep-brain stimulation for Parkinson's disease using image-guided frameless stereotaxy. **Neurosurgery 67:**1088-1093; discussion 1093, 2010

11. Christoffel DJ, Golden SA, Walsh JJ, Guise KG, Heshmati M, Friedman AK, Dey A, Smith M, Rebusi N, Pfau M, Ables JL, Aleyasin H, Khibnik LA, Hodes GE, Ben-Dor GA, Deisseroth K, Shapiro ML, Malenka RC, Ibanez-Tallon I, Han MH, Russo SJ: Excitatory transmission at thalamo-striatal synapses mediates susceptibility to social stress. **Nat Neurosci 18:**962-964, 2015 4482771

12. Christopher PP, Leykin Y, Appelbaum PS, Holtzheimer PE, 3rd, Mayberg HS, Dunn LB: Enrolling in deep brain stimulation research for depression: influences on potential subjects' decision making. **Depress Anxiety 29:**139-146, 2012

13. Colles SL, Dixon JB, O'Brien PE: Loss of control is central to psychological disturbance associated with binge eating disorder. **Obesity (Silver Spring) 16:**608-614, 2008

14. Denys D, Mantione M, Figee M, van den Munckhof P, Koerselman F, Westenberg H, Bosch A, Schuurman R: Deep brain stimulation of the nucleus accumbens for treatment-refractory obsessive-compulsive disorder. **Arch Gen Psychiatry 67:**1061-1068, 2010

15. Desai SA, Rolston JD, McCracken CE, Potter SM, Gross RE: Asynchronous Distributed Multielectrode Microstimulation Reduces Seizures in the Dorsal Tetanus Toxin Model of Temporal Lobe Epilepsy. **Brain Stimul 9:**86-100, 2016 PMC4724241

16. Dong Y, Hoover A, Scisco J, Muth E: A new method for measuring meal intake in humans via automated wrist motion tracking. **Appl Psychophysiol Biofeedback 37:**205-215, 2012 PMC4487660

17. Dougherty DD, Rezai AR, Carpenter LL, Howland RH, Bhati MT, O'Reardon JP, Eskandar EN, Baltuch GH, Machado AD, Kondziolka D, Cusin C, Evans KC, Price LH, Jacobs K, Pandya M, Denko T, Tyrka AR, Brelje T, Deckersbach T, Kubu C, Malone DA, Jr.: A Randomized Sham-Controlled Trial of Deep Brain Stimulation of the Ventral Capsule/Ventral Striatum for Chronic Treatment-Resistant Depression. **Biol Psychiatry 78:**240-248, 2015

18. Engel J, Jr., Wiebe S, French J, Sperling M, Williamson P, Spencer D, Gumnit R, Zahn C, Westbrook E, Enos B: Practice parameter: temporal lobe and localized neocortical resections for epilepsy: report of the Quality Standards Subcommittee of the American Academy of Neurology, in association with the

American Epilepsy Society and the American Association of Neurological Surgeons. **Neurology 60:**538-547, 2003
19. Engstrom M, Forsberg A, Sovik TT, Olbers T, Lonroth H, Karlsson J: Perception of control over eating after bariatric surgery for super-obesity--a 2-year follow-up study. **Obes Surg 25:**1086-1093, 2015
20. Finkelstein EA, Trogdon JG, Cohen JW, Dietz W: Annual medical spending attributable to obesity: payer-and service-specific estimates. **Health Aff (Millwood) 28:**w822-831, 2009
21. Fisher CE, Dunn LB, Christopher PP, Holtzheimer PE, Leykin Y, Mayberg HS, Lisanby SH, Appelbaum PS: The ethics of research on deep brain stimulation for depression: decisional capacity and therapeutic misconception. **Ann N Y Acad Sci 1265:**69-79, 2012 3624886
22. Fisher R, Salanova V, Witt T, Worth R, Henry T, Gross R, Oommen K, Osorio I, Nazzaro J, Labar D, Kaplitt M, Sperling M, Sandok E, Neal J, Handforth A, Stern J, DeSalles A, Chung S, Shetter A, Bergen D, Bakay R, Henderson J, French J, Baltuch G, Rosenfeld W, Youkilis A, Marks W, Garcia P, Barbaro N, Fountain N, Bazil C, Goodman R, McKhann G, Babu Krishnamurthy K, Papavassiliou S, Epstein C, Pollard J, Tonder L, Grebin J, Coffey R, Graves N: Electrical stimulation of the anterior nucleus of thalamus for treatment of refractory epilepsy. **Epilepsia 51:**899-908, 2010
23. Fontaine KR, Barofsky I: Obesity and health-related quality of life. **Obes Rev 2:**173-182, 2001
24. Fontaine KR, Redden DT, Wang C, Westfall AO, Allison DB: Years of life lost due to obesity. **JAMA 289:**187-193, 2003
25. Fray PJ, Robbins TW: CANTAB battery: proposed utility in neurotoxicology. **Neurotoxicol Teratol 18:**499-504, 1996
26. Ghazizadeh A, Ambroggi F, Odean N, Fields HL: Prefrontal cortex mediates extinction of responding by two distinct neural mechanisms in accumbens shell. **J Neurosci 32:**726-737, 2012
27. Giel KE, Zipfel S, Schweizer R, Braun R, Ranke MB, Binder G, Ehehalt S: Eating disorder pathology in adolescents participating in a lifestyle intervention for obesity: associations with weight change, general psychopathology and health-related quality of life. **Obes Facts 6:**307-316, 2013: Striatal dopamine in bulimia nervosa: a PET imaging study. **Int J Eat Disord 45:**648-656, 2012 PMC3640453
28. Goldschmidt AB, Engel SG, Wonderlich SA, Crosby RD, Peterson CB, Le Grange D, Tanofsky-Kraff M, Cao L, Mitchell JE: Momentary affect surrounding loss of control and overeating in obese adults with and without binge eating disorder. **Obesity (Silver Spring) 20:**1206-1211, 2012 PMC3816927
29. Goodman JH, Berger RE, Tcheng TK: Preemptive low-frequency stimulation decreases the incidence of amygdala-kindled seizures. **Epilepsia 46:**1-7, 2005
30. Goodman WK, Foote KD, Greenberg BD, Ricciuti N, Bauer R, Ward H, Shapira NA, Wu SS, Hill CL, Rasmussen SA, Okun MS: Deep brain stimulation for intractable obsessive compulsive disorder: pilot study using a blinded, staggered-onset design. **Biol Psychiatry 67:**535-542, 2010
31. Gorin AA, Niemeier HM, Hogan P, Coday M, Davis C, DiLillo VG, Gluck ME, Wadden TA, West DS, Williamson D, Yanovski SZ: Binge eating and weight loss outcomes in overweight and obese individuals with type 2 diabetes: results from the Look AHEAD trial. **Arch Gen Psychiatry 65:**1447-1455, 2008 PMC2791958
32. Grilo CM, Masheb RM, Wilson GT: Different methods for assessing the features of eating disorders in patients with binge eating disorder: a replication. **Obes Res 9:**418-422, 2001
33. Grilo CM, Masheb RM, Wilson GT, Gueorguieva R, White MA: Cognitive-behavioral therapy, behavioral weight loss, and sequential treatment for obese patients with binge-eating disorder: a randomized controlled trial. **J Consult Clin Psychol 79:**675-685, 2011 PMC3258572
34. Groen WG, Kuijpers W, Oldenburg HS, Wouters MW, Aaronson NK, van Harten WH: Empowerment of Cancer Survivors Through Information Technology: An Integrative Review. **J Med Internet Res 17:**e270, 2015 PMC4704924
35. Guh DP, Zhang W, Bansback N, Amarsi Z, Birmingham CL, Anis AH: The incidence of co-morbidities related to obesity and overweight: a systematic review and meta-analysis. **BMC Public Health 9:**88, 2009 PMC2667420
36. Halperin F, Ding SA, Simonson DC, Panosian J, Goebel-Fabbri A, Wewalka M, Hamdy O, Abrahamson M, Clancy K, Foster K, Lautz D, Vernon A, Goldfine AB: Roux-en-Y gastric bypass surgery

or lifestyle with intensive medical management in patients with type 2 diabetes: feasibility and 1-year results of a randomized clinical trial. **JAMA Surg 149:**716-726, 2014 PMC4274782
37. Halpern C, Attiah M, Bale T: **Deep brain stimulation for the treatment of binge eating: mechanisms and preclinical models. In: Animal Models of Eating Disorders.** Totowa N.J.: Humana Press, 2013
38. Halpern C, Hurtig H, Jaggi J, Grossman M, Won M, Baltuch G: Deep brain stimulation in neurologic disorders. **Parkinsonism Relat Disord 13:**1-16, 2007
39. Halpern CH, Tekriwal A, Santollo J, Keating JG, Wolf JA, Daniels D, Bale TL: Amelioration of binge eating by nucleus accumbens shell deep brain stimulation in mice involves D2 receptor modulation. **J Neurosci 33:**7122-7129, 2013 PMC3703148
40. Halpern CH, Torres N, Hurtig HI, Wolf JA, Stephen J, Oh MY, Williams NN, Dichter MA, Jaggi JL, Caplan AL, Kampman KM, Wadden TA, Whiting DM, Baltuch GH: Expanding applications of deep brain stimulation: a potential therapeutic role in obesity and addiction management. **Acta Neurochir (Wien) 153:**2293-2306, 2011
41. Halpern CH, Wolf JA, Bale TL, Stunkard AJ, Danish SF, Grossman M, Jaggi JL, Grady MS, Baltuch GH: Deep brain stimulation in the treatment of obesity. **J Neurosurg 109:**625-634, 2008
42. Hamani C, Diwan M, Isabella S, Lozano AM, Nobrega JN: Effects of different stimulation parameters on the antidepressant-like response of medial prefrontal cortex deep brain stimulation in rats. **J Psychiatr Res 44:**683-687, 2010
43. Haq IU, Foote KD, Goodman WG, Wu SS, Sudhyadhom A, Ricciuti N, Siddiqui MS, Bowers D, Jacobson CE, Ward H, Okun MS: Smile and laughter induction and intraoperative predictors of response to deep brain stimulation for obsessive-compulsive disorder. **Neuroimage 54 Suppl 1:**S247-255, 2011 PMC2907450
44. Heck CN, King-Stephens D, Massey AD, Nair DR, Jobst BC, Barkley GL, Salanova V, Cole AJ, Smith MC, Gwinn RP, Skidmore C, Van Ness PC, Bergey GK, Park YD, Miller I, Geller E, Rutecki PA, Zimmerman R, Spencer DC, Goldman A, Edwards JC, Leiphart JW, Wharen RE, Fessler J, Fountain NB, Worrell GA, Gross RE, Eisenschenk S, Duckrow RB, Hirsch LJ, Bazil C, O'Donovan CA, Sun FT, Courtney TA, Seale CG, Morrell MJ: Two-year seizure reduction in adults with medically intractable partial onset epilepsy treated with responsive neurostimulation: final results of the RNS System Pivotal trial. **Epilepsia 55:**432-441, 2014 PMC4233950
45. Heilbronner SR, Rodriguez-Romaguera J, Quirk GJ, Groenewegen HJ, Haber SN: Circuit-Based Corticostriatal Homologies Between Rat and Primate. **Biol Psychiatry 80:**509-521, 2016
46. Hsu LK, Sullivan SP, Benotti PN: Eating disturbances and outcome of gastric bypass surgery: a pilot study. **Int J Eat Disord 21:**385-390, 199747. Hudson JI, Hiripi E, Pope HG, Jr., Kessler RC: The prevalence and correlates of eating disorders in the National Comorbidity Survey Replication. **Biol Psychiatry 61:**348-358, 2007 PMC1892232
48. Ivezaj V, Kessler EE, Lydecker JA, Barnes RD, White MA, Grilo CM: Loss-of-control eating after sleeve gastrectomy surgery. **Surg Obes Relat Dis**, 2016
49. Jarcho JM, Tanofsky-Kraff M, Nelson EE, Engel SG, Vannucci A, Field SE, Romer AL, Hannallah L, Brady SM, Demidowich AP, Shomaker LB, Courville AB, Pine DS, Yanovski JA: Neural activation during anticipated peer evaluation and laboratory meal intake in overweight girls with and without loss of control eating. **Neuroimage 108:**343-353, 2015 PMC4323624
50. Johnson PM, Kenny PJ: Dopamine D2 receptors in addiction-like reward dysfunction and compulsive eating in obese rats. **Nat Neurosci 13:**635-641, 2010 PMC2947358
51. Kerrigan JF, Litt B, Fisher RS, Cranstoun S, French JA, Blum DE, Dichter M, Shetter A, Baltuch G, Jaggi J, Krone S, Brodie M, Rise M, Graves N: Electrical stimulation of the anterior nucleus of the thalamus for the treatment of intractable epilepsy. **Epilepsia 45:**346-354, 2004
52. Kiernan M, Moore SD, Schoffman DE, Lee K, King AC, Taylor CB, Kiernan NE, Perri MG: Social support for healthy behaviors: scale psychometrics and prediction of weight loss among women in a behavioral program. **Obesity (Silver Spring) 20:**756-764, 2012 PMC4718570

53. King-Stephens D, Mirro E, Weber PB, Laxer KD, Van Ness PC, Salanova V, Spencer DC, Heck CN, Goldman A, Jobst B, Shields DC, Bergey GK, Eisenschenk S, Worrell GA, Rossi MA, Gross RE, Cole AJ, Sperling MR, Nair DR, Gwinn RP, Park YD, Rutecki PA, Fountain NB, Wharen RE, Hirsch LJ, Miller IO, Barkley GL, Edwards JC, Geller EB, Berg MJ, Sadler TL, Sun FT, Morrell MJ: Lateralization of mesial temporal lobe epilepsy with chronic ambulatory electrocorticography. **Epilepsia 56:**959-967, 2015 PMC4676303
54. Knutson B, Westdorp A, Kaiser E, Hommer D: FMRI visualization of brain activity during a monetary incentive delay task. **Neuroimage 12:**20-27, 2000
55. Kombian SB, Malenka RC: Simultaneous LTP of non-NMDA- and LTD of NMDA-receptor-mediated responses in the nucleus accumbens. **Nature 368:**242-246, 1994
56. Krause M, German PW, Taha SA, Fields HL: A pause in nucleus accumbens neuron firing is required to initiate and maintain feeding. **J Neurosci 30:**4746-4756, 2010 PMC2878763
57. Kroemer NB, Sun X, Veldhuizen MG, Babbs AE, de Araujo IE, Small DM: Weighing the evidence: Variance in brain responses to milkshake receipt is predictive of eating behavior. **Neuroimage 128:**273-283, 2016
58. Lee SH, Kim KH, Cheong SM, Kim S, Kooh M, Chin DK: A comparison of the effect of epidural patient-controlled analgesia with intravenous patient-controlled analgesia on pain control after posterior lumbar instrumented fusion. **J Korean Neurosurg Soc 50:**205-208, 2011 PMC3218179
59. Liu SY, Wong SK, Lam CC, Yung MY, Kong AP, Ng EK: Long-term Results on Weight Loss and Diabetes Remission after Laparoscopic Sleeve Gastrectomy for A Morbidly Obese Chinese Population. **Obes Surg**, 2015
60. Loring DW, Kapur R, Meador KJ, Morrell MJ: Differential neuropsychological outcomes following targeted responsive neurostimulation for partial-onset epilepsy. **Epilepsia 56:**1836-1844, 2015
61. Lucas-Neto L, Reimao S, Oliveira E, Rainha-Campos A, Sousa J, Nunes RG, Goncalves-Ferreira A, Campos JG: Advanced MR Imaging of the Human Nucleus Accumbens--Additional Guiding Tool for Deep Brain Stimulation. **Neuromodulation 18:**341-348, 2015
62. Maling N, Hashemiyoon R, Foote KD, Okun MS, Sanchez JC: Increased thalamic gamma band activity correlates with symptom relief following deep brain stimulation in humans with Tourette's syndrome. **PLoS One 7:**e44215, 2012 PMC3435399
63. Mantione M, van de Brink W, Schuurman PR, Denys D: Smoking cessation and weight loss after chronic deep brain stimulation of the nucleus accumbens: therapeutic and research implications: case report. **Neurosurgery 66:**E218; discussion E218, 2010
64. McCreery DB, Agnew WF, Yuen TG, Bullara L: Charge density and charge per phase as cofactors in neural injury induced by electrical stimulation. **IEEE Trans Biomed Eng 37:**996-1001, 1990
65. McElroy SL, Hudson JI, Mitchell JE, Wilfley D, Ferreira-Cornwell MC, Gao J, Wang J, Whitaker T, Jonas J, Gasior M: Efficacy and safety of lisdexamfetamine for treatment of adults with moderate to severe binge-eating disorder: a randomized clinical trial. **JAMA Psychiatry 72:**235-246, 2015
66. Meador KJ, Kapur R, Loring DW, Kanner AM, Morrell MJ: Quality of life and mood in patients with medically intractable epilepsy treated with targeted responsive neurostimulation. **Epilepsy Behav 45:**242-247, 201567. Moize V, Andreu A, Flores L, Torres F, Ibarzabal A, Delgado S, Lacy A, Rodriguez L, Vidal J: Long-term dietary intake and nutritional deficiencies following sleeve gastrectomy or Roux-En-Y gastric bypass in a mediterranean population. **J Acad Nutr Diet 113:**400-410, 2013
68. Morrell MJ: Responsive cortical stimulation for the treatment of medically intractable partial epilepsy. **Neurology 77:**1295-1304, 2011
69. O'Connor EC, Kremer Y, Lefort S, Harada M, Pascoli V, Rohner C, Luscher C: Accumbal D1R Neurons Projecting to Lateral Hypothalamus Authorize Feeding. **Neuron 88:**553-564, 2015
70. Ogden CL, Carroll MD, Kit BK, Flegal KM: Prevalence of childhood and adult obesity in the United States, 2011-2012. **JAMA 311:**806-814, 2014 PMC4770258
71. Ogden CL, Carroll MD, Kit BK, Flegal KM: Prevalence of obesity in the United States, 2009-2010. **NCHS Data Brief:**1-8, 2012

72. Okun MS, Foote KD, Wu SS, Ward HE, Bowers D, Rodriguez RL, Malaty IA, Goodman WK, Gilbert DM, Walker HC, Mink JW, Merritt S, Morishita T, Sanchez JC: A trial of scheduled deep brain stimulation for Tourette syndrome: moving away from continuous deep brain stimulation paradigms. **JAMA Neurol 70:**85-94, 2013
73. Ooms P, Mantione M, Figee M, Schuurman PR, van den Munckhof P, Denys D: Deep brain stimulation for obsessive-compulsive disorders: long-term analysis of quality of life. **J Neurol Neurosurg Psychiatry 85:**153-158, 2014
74. Pisapia JM, Halpern CH, Williams NN, Wadden TA, Baltuch GH, Stein SC: Deep brain stimulation compared with bariatric surgery for the treatment of morbid obesity: a decision analysis study. **Neurosurg Focus 29:**E15, 2010
75. Reitan RM, Wolfson D: **The Halstead-Reitan neuropsychological test battery : theory and clinical interpretation.** Tucson, Ariz.: Neuropsychology Press, 1985
76. Risinger MW, Gumnit RJ: Intracranial electrophysiologic studies. **Neuroimaging Clin N Am 5:**559-573, 1995
77. Robinson AH, Adler S, Stevens HB, Darcy AM, Morton JM, Safer DL: What variables are associated with successful weight loss outcomes for bariatric surgery after 1 year? **Surg Obes Relat Dis 10:**697-704, 2014 PMC4125556
78. Safer DL, Robinson AH, Jo B: Outcome from a randomized controlled trial of group therapy for binge eating disorder: comparing dialectical behavior therapy adapted for binge eating to an active comparison group therapy. **Behav Ther 41:**106-120, 2010 PMC3170852
79. Salanova V, Witt T, Worth R, Henry TR, Gross RE, Nazzaro JM, Labar D, Sperling MR, Sharan A, Sandok E, Handforth A, Stern JM, Chung S, Henderson JM, French J, Baltuch G, Rosenfeld WE, Garcia P, Barbaro NM, Fountain NB, Elias WJ, Goodman RR, Pollard JR, Troster AI, Irwin CP, Lambrecht K, Graves N, Fisher R: Long-term efficacy and safety of thalamic stimulation for drug-resistant partial epilepsy. **Neurology 84:**1017-1025, 2015 PMC4352097
80. Siebenhofer A, Jeitler K, Horvath K, Berghold A, Posch N, Meschik J, Semlitsch T: Long-term effects of weight-reducing drugs in people with hypertension. **Cochrane Database Syst Rev 3:**CD007654, 2016
81. Sillay KA, Rutecki P, Cicora K, Worrell G, Drazkowski J, Shih JJ, Sharan AD, Morrell MJ, Williams J, Wingeier B: Long-term measurement of impedance in chronically implanted depth and subdural electrodes during responsive neurostimulation in humans. **Brain Stimul 6:**718-726, 2013
82. Stenner MP, Litvak V, Rutledge RB, Zaehle T, Schmitt FC, Voges J, Heinze HJ, Dolan RJ: Cortical drive of low-frequency oscillations in the human nucleus accumbens during action selection. **J Neurophysiol 114:**29-39, 2015 PMC4518721
83. Stice E, Spoor S, Bohon C, Small DM: Relation between obesity and blunted striatal response to food is moderated by TaqIA A1 allele. **Science 322:**449-452, 2008 PMC2681095
84. Strand AD, Aragaki AK, Baquet ZC, Hodges A, Cunningham P, Holmans P, Jones KR, Jones L, Kooperberg C, Olson JM: Conservation of regional gene expression in mouse and human brain. **PLoS Genet 3:**e59, 2007 PMC1853119
85. Stunkard A, Berkowitz R, Tanrikut C, Reiss E, Young L: d-fenfluramine treatment of binge eating disorder. **Am J Psychiatry 153:**1455-1459, 1996
86. Tanriverdi T, Ajlan A, Poulin N, Olivier A: Morbidity in epilepsy surgery: an experience based on 2449 epilepsy surgery procedures from a single institution. **J Neurosurg 110:**1111-1123, 2009
87. Teegarden SL, Bale TL: Decreases in dietary preference produce increased emotionality and risk for dietary relapse. **Biol Psychiatry 61:**1021-1029, 2007
88. Teegarden SL, Bale TL: Effects of stress on dietary preference and intake are dependent on access and stress sensitivity. **Physiol Behav 93:**713-723, 2008 PMC248332889. Telch CF, Agras WS: Do emotional states influence binge eating in the obese? **Int J Eat Disord 20:**271-279, 1996
90. Tortorella A, Volpe U, Fabrazzo M, Tolone S, Docimo L, Monteleone P: From over- to under-weight: treatment of post-surgical anorexia nervosa in morbid obesity. **Eat Weight Disord 20:**529-532, 2015
91. Tulsky D, Zhu J, Ledbetter M, (eds): **WAIS-III WMS-III Technical Manual (Wechsler Adult Intelligence Scale & Wechsler Memory Scale), ed 3rd Edition**: Harcourt Brace & Company, 1997

92. Vassoler FM, Schmidt HD, Gerard ME, Famous KR, Ciraulo DA, Kornetsky C, Knapp CM, Pierce RC: Deep brain stimulation of the nucleus accumbens shell attenuates cocaine priming-induced reinstatement of drug seeking in rats. **J Neurosci 28:**8735-8739, 2008 2585378
93. Vidal P, Ramon JM, Goday A, Parri A, Crous X, Trillo L, Pera M, Grande L: Lack of adherence to follow-up visits after bariatric surgery: reasons and outcome. **Obes Surg 24:**179-183, 2014
94. Volkow ND, Wise RA: How can drug addiction help us understand obesity? **Nat Neurosci 8:**555-560, 2005
95. Wadden TA, Butryn ML, Byrne KJ: Efficacy of lifestyle modification for long-term weight control. **Obes Res 12 Suppl:**151S-162S, 2004
96. Wagenaar DA, Pine J, Potter SM: Effective parameters for stimulation of dissociated cultures using multi-electrode arrays. **J Neurosci Methods 138:**27-37, 2004
97. Wang GJ, Volkow ND, Logan J, Pappas NR, Wong CT, Zhu W, Netusil N, Fowler JS: Brain dopamine and obesity. **Lancet 357:**354-357, 2001
98. Weaver FM, Follett K, Stern M, Hur K, Harris C, Marks WJ, Jr., Rothlind J, Sagher O, Reda D, Moy CS, Pahwa R, Burchiel K, Hogarth P, Lai EC, Duda JE, Holloway K, Samii A, Horn S, Bronstein J, Stoner G, Heemskerk J, Huang GD: Bilateral deep brain stimulation vs best medical therapy for patients with advanced Parkinson disease: a randomized controlled trial. **JAMA 301:**63-73, 2009 PMC2814800
99. Weygandt M, Mai K, Dommes E, Leupelt V, Hackmack K, Kahnt T, Rothemund Y, Spranger J, Haynes JD: The role of neural impulse control mechanisms for dietary success in obesity. **Neuroimage 83:**669-678, 2013
100. White MA, Kalarchian MA, Masheb RM, Marcus MD, Grilo CM: Loss of control over eating predicts outcomes in bariatric surgery patients: a prospective, 24-month follow-up study. **J Clin Psychiatry 71:**175-184, 2010 PMC2831110
101. Wonderlich JA, Lavender JM, Wonderlich SA, Peterson CB, Crow SJ, Engel SG, Le Grange D, Mitchell JE, Crosby RD: Examining convergence of retrospective and ecological momentary assessment measures of negative affect and eating disorder behaviors. **Int J Eat Disord 48:**305-311, 2015
102. Wu H, Nica I, Tambuyzer T, Deprez M, Kuyck K, Aerts JM, VanHuffel S, Nuttin B, Malenka RC, Halpern C: Closed-loop brain stimulation for psychiatric disorders: evidence from rodent and human studies, in **European Society for Stereotactic and Functional Neurosurgery**, 2016
103. Wu H, Ravikumar V, Blumenfeld Z, Lee K, Bronte-Stewart H, Knutson B, Malenka RC, Halpern C: Low-frequency oscillations of the nucleus accumbens may close the loop on impulsivity in **American Academy of Neurological Surgeons**, 2016
104. Berg, K. C., R. D. Crosby, L. Cao, S. J. Crow, S. G. Engel, S. A. Wonderlich, and C. B. Peterson. 2015. 'Negative affect prior to and following overeating-only, loss of control eating-only, and binge eating episodes in obese adults', *Int J Eat Disord*, 48: 641-53.
105. Blomquist, K. K., C. A. Roberto, R. D. Barnes, M. A. White, R. M. Masheb, and C. M. Grilo. 2014. 'Development and validation of the eating loss of control scale', *Psychol Assess*, 26: 77-89.
106. Dougherty, D. D., A. R. Rezai, L. L. Carpenter, R. H. Howland, M. T. Bhati, J. P. O'Reardon, E. N.
107. Eskandar, G. H. Baltuch, A. D. Machado, D. Kondziolka, C. Cusin, K. C. Evans, L. H. Price, K. Jacobs, M. Pandya, T. Denko, A. R. Tyrka, T. Brelje, T. Deckersbach, C. Kubu, and D. A. Malone, Jr. 2015. 'A Randomized Sham-Controlled Trial of Deep Brain Stimulation of the Ventral Capsule/Ventral Striatum for Chronic Treatment-Resistant Depression', *Biol Psychiatry*, 78: 240-8.
108. Fairburn, C. G., and S. J. Beglin. 1994. 'Assessment of eating disorders: interview or self-report questionnaire?', *Int J Eat Disord*, 16: 363-70.
109. Goodman, W. K., K. D. Foote, B. D. Greenberg, N. Ricciuti, R. Bauer, H. Ward, N. A. Shapira, S. S. Wu, C. L. Hill, S. A. Rasmussen, and M. S. Okun. 2010. 'Deep brain stimulation for intractable obsessive compulsive disorder: pilot study using a blinded, staggered-onset design', *Biol Psychiatry*, 67: 535-42.
110. Maldonado, J. R., H. C. Dubois, E. E. David, Y. Sher, S. Lolak, J. Dyal, and D. Witten. 2012. 'The Stanford Integrated Psychosocial Assessment for Transplantation (SIPAT): a new tool for the psychosocial evaluation of pre-transplant candidates', *Psychosomatics*, 53: 123-32.
111. Mason, T. B., K. E. Smith, R. D. Crosby, S. A. Wonderlich, S. J. Crow, S. G. Engel, and C. B. Peterson. 2017. 'Does the eating disorder examination questionnaire global subscale adequately predict eating

disorder psychopathology in the daily life of obese adults?', *Eat Weight Disord*.
112. Okun, M. S., K. D. Foote, S. S. Wu, H. E. Ward, D. Bowers, R. L. Rodriguez, I. A. Malaty, W. K. Goodman, D. M. Gilbert, H. C. Walker, J. W. Mink, S. Merritt, T. Morishita, and J. C. Sanchez. 2013. 'A trial of scheduled deep brain stimulation for Tourette syndrome: moving away from continuous deep brain stimulation paradigms', *JAMA Neurol*, 70: 85-94.
113. Telch, C. F., and W. S. Agras. 1996. 'Do emotional states influence binge eating in the obese?', *Int J Eat Disord*, 20: 271-9.

## 9. LIST OF FIGURES AND TABLES

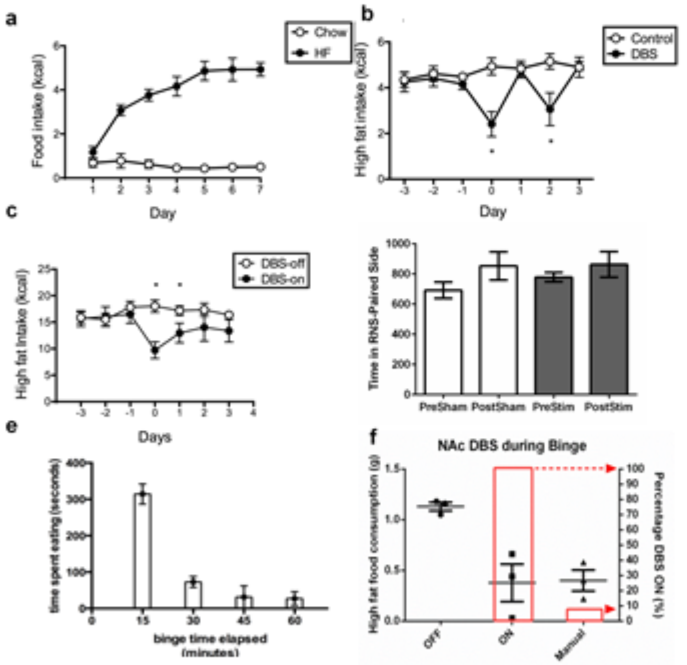


**Figure 1**: **a.** Mice exhibited increased intake until stabilizing by day 5. **b**. Stimulation administered only acutely blocked intake on 2 alternate days. **c.** NAc DBS suppressed daily food intake when delivered chronically, but there was loss of significance after Day 2. **d,** NAc stimulation did not induce a place preference, suggesting the effects were not reinforcing. **e,** Mice provided with a 1h exposure to high fat exhibit episodic binge behavior. **f,** Manually triggering stimulation at these binge onsets under synchronized video surveillance blocks intake with <10% less stim-on time. HF, high-fat; DBS, deep brain stimulation; *p<0.05

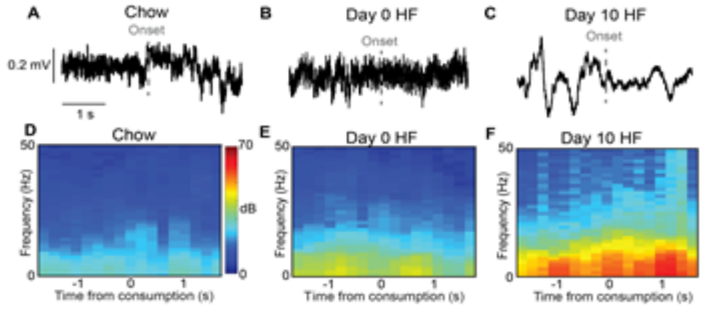


Fig. 2. Raw Local Field Potentials (LFPs), Power Spectral Density, Time-Frequency Analyses of the NAc LFPs. (A-C) Raw LFPs are shown during the onset (dotted grey line) of house chow as a control and high-fat (HF) consumption on days 0 and 10 (before and after the development of binge-like behavior, respectively). (D-F) Mean power spectrogram of LFPs immediately before and during chow and HF consumption.

Fig. 2, cont'd. Delta Power Characterization and System Block Diagram of the Responsive Neurostimulation Setup. (G) Power spectral density analysis of LFPs immediately before (2-second window) chow and HF intake on days 0 and 10, averaged across individual mice, revealing higher power in low-frequency oscillations immediately prior to the onset of HF intake on day 10. (H) Mean delta power significantly increased immediately prior to the onset of HF intake on day 10 compared to HF intake on day 0 and chow intake. (I) Delta power percent-change-over-baseline during the onset of HF intake on day 10 (normalized to the 1-hr period of HF exposure). (J) Delta power peak distribution before the onset HF consumption on day 10. (K) Mean power spectrogram of NAc LFP during the onset of juvenile interaction. (L) System block diagram of the responsive neurostimulation setup, which consisted of a 1x follower cable for unit amplification, a headstage for analog/digital conversion, a digital filter, a computer for synchronizing neural electrophysiological and behavioral data, a prototype biomarker detector (Neurostimulator, Model RNS-300), a constant-current stimulator, and a charge-coupled device camera for synchronized behavioral recording. **P < 0.01.

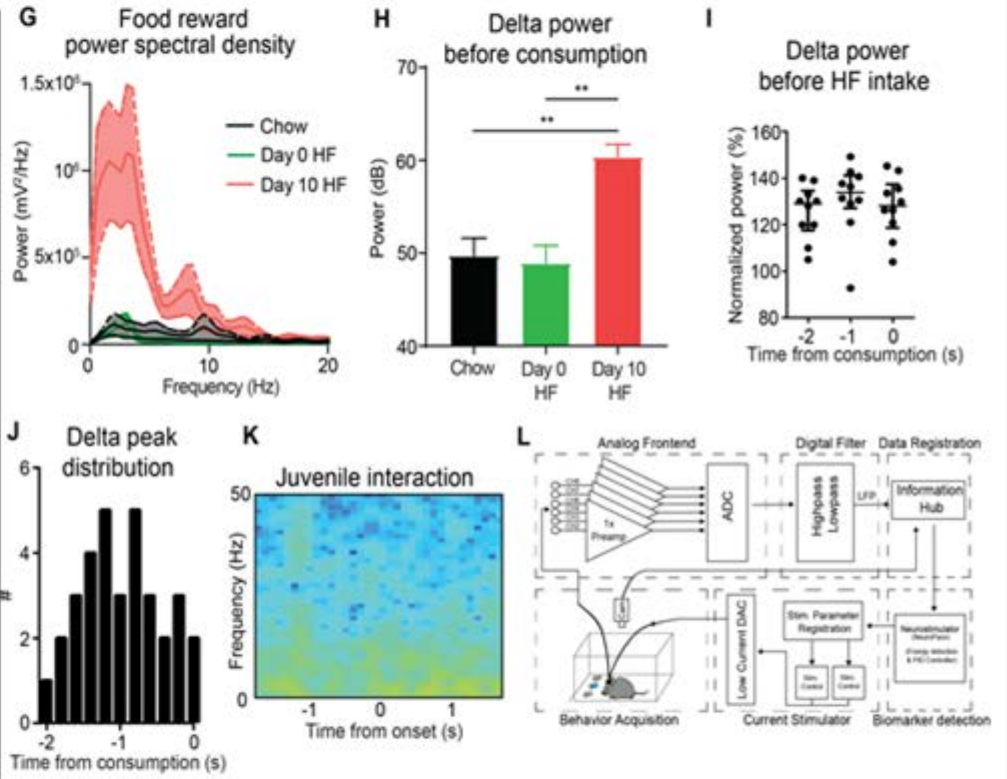

**9.3**

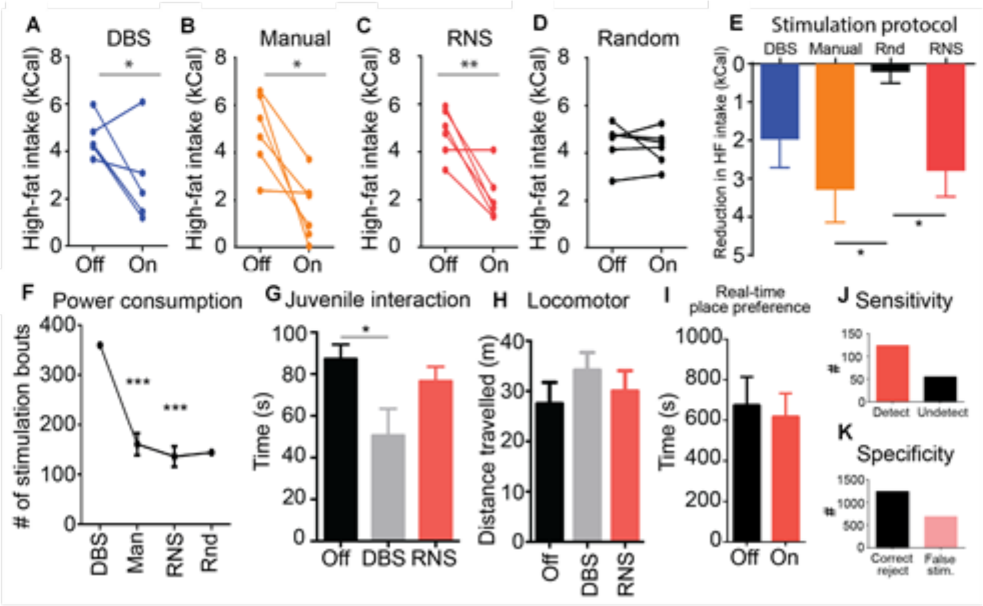


Fig. 3. Result Summary of Different Electrical Stimulation Protocols on HF Intake and Juvenile Interaction. (A-D) The effects of different stimulation protocols on HF consumption. (E) Reduction in HF intake induced by DBS, manually-triggered stimulation, randomly-applied stimulation (Rnd) and RNS. The reduction in HF intake induced by the manually-triggered stimulation and RNS was significantly higher than randomly-applied stimulation. (F) The number of stimulations bouts (1 bout=10 s) delivered during Manual and RNS was significantly lower than DBS. (G) DBS of the NAc significantly reduced juvenile interaction time, while RNS showed no effect on this behavior. (H) Neither DBS nor RNS of the NAc during HF exposure significantly altered locomotor activity. (I) Real-time place preference test suggested that NAc stimulation was neither rewarding nor aversive. (J-K) Sensitivity and specificity of delta on RNS. In total there were 179 HF pellet approaches, of which 124 were detected by the RNS system (sensitivity=0.693). There were also 1241 correct rejections (stimulation off when no HF approach occurred) and 685 stimulations triggered when no HF approaches were observed (false stimulation; specificity=0.644). *P < 0.05. **P < 0.01. ***P < 0.001.

**9.4**

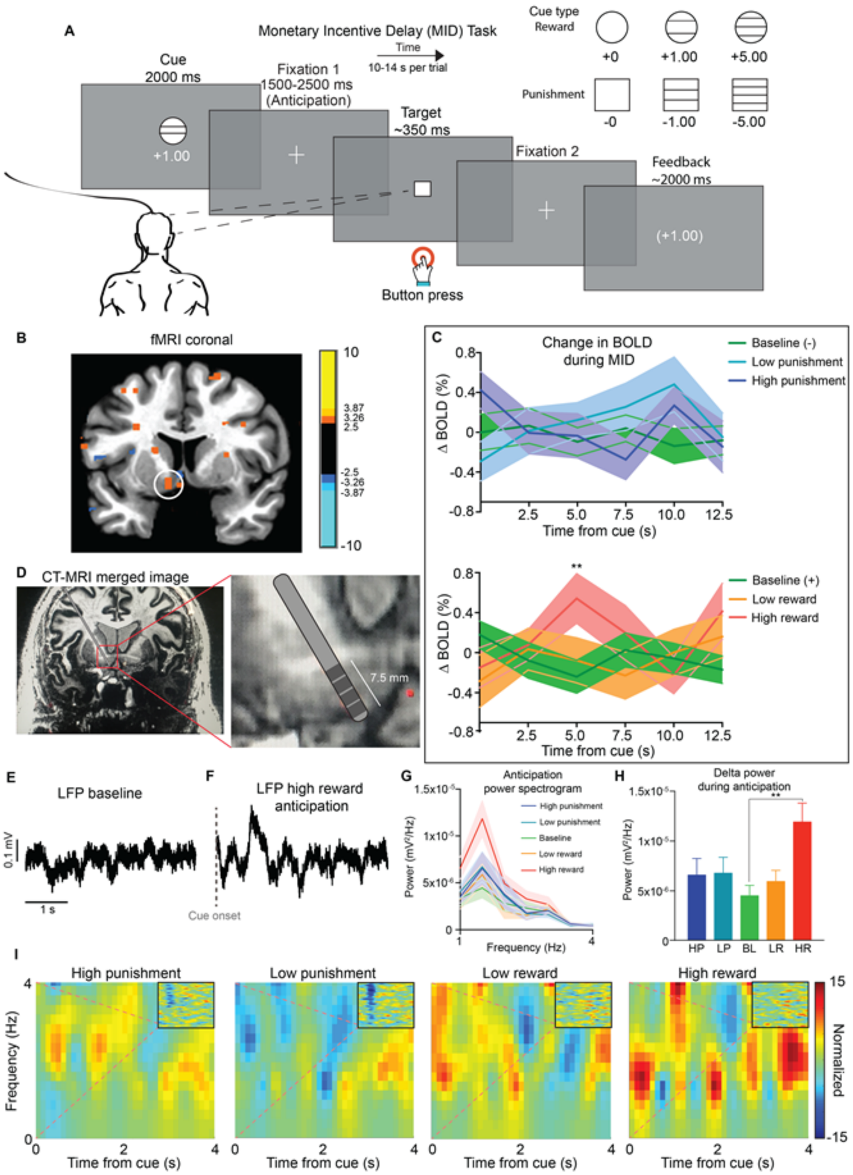

A
Monetary Incentive Delay (MID) Task
Time
10-14 s per trial
Cue type
Reward
+0
+1.00
+5.00
Punishment
-0
-1.00
-5.00
Cue
2000 ms
+1.00
Fixation 1
1500-2500 ms
(Anticipation)
Target
~350 ms
Fixation 2
Feedback
~2000 ms
(+1.00)
Button press
B
fMRI coronal
C
Change in BOLD
during MID
Baseline (-)
Low punishment
High punishment
Δ BOLD (%)
Time from cue (s)
Baseline (+)
Low reward
High reward
D
CT-MRI merged image
7.5 mm
E
LFP baseline
0.1 mV
1 s
F
LFP high reward
anticipation
Cue onset
G
Anticipation
power spectrogram
Frequency (Hz)
H
Delta power
during anticipation
HP
LP
BL
LR
HR
I
High punishment
Low punishment
Low reward
High reward
Frequency (Hz)
Time from cue (s)
Normalized

### 9.5 The RNS System and Components

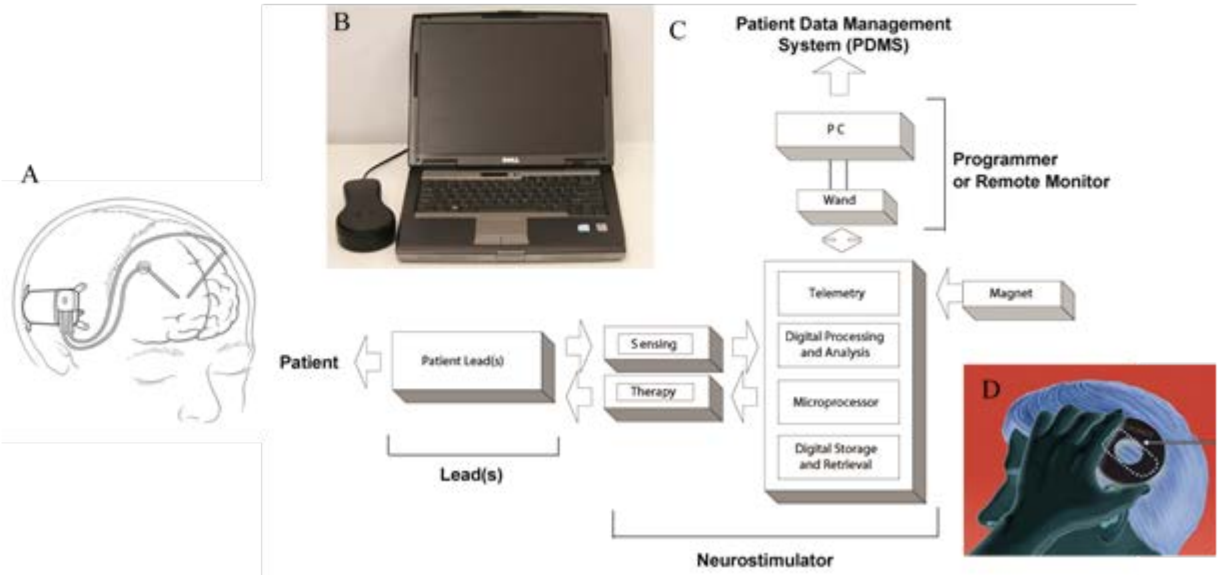


**System overview.** A. 3D schematic of neurostimulator and NAc depths. B. Patient programmer and remote monitor. C. Schematic of how the RNS® Neurostimulator senses and delivers therapy via the implanted patient leads.

### 9.6 RNS System Overview

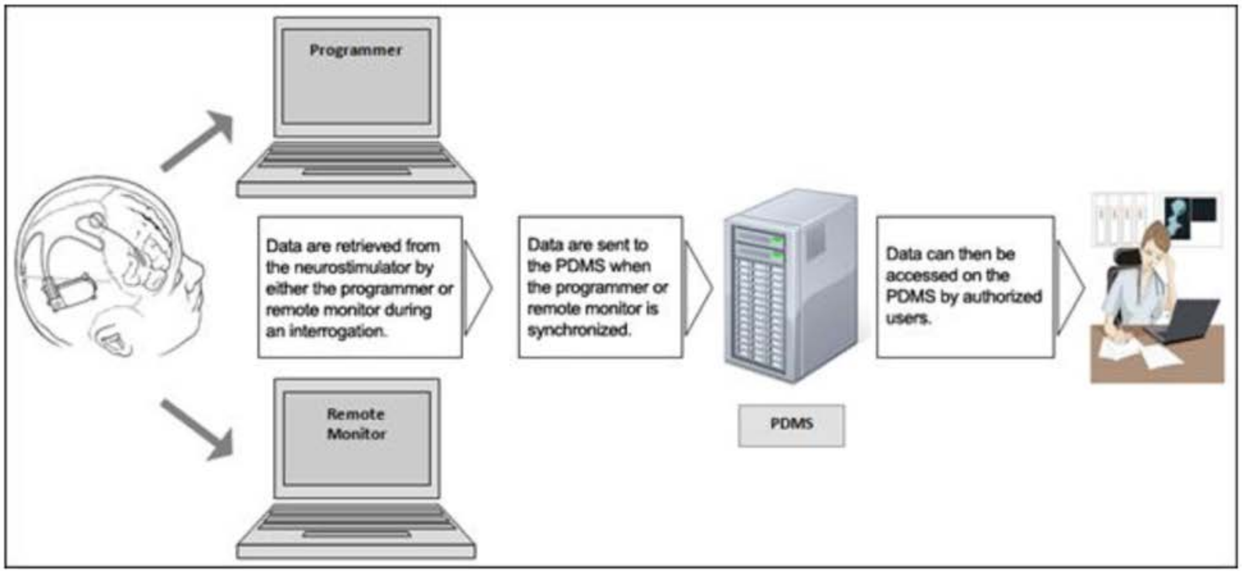


**System Overview.** Communication with the neurostimulator is available using the programmer (and remote monitor) as in B, as well as with the magnet. D, The magnet has two uses: 1) to instruct the neurostimulator to make a recording of brain activity; 2) to stop stimulation if need be.

## 9.7 Schedule of Assessments

| Closed-Loop Neurostimulation for Loss of Control Eating | Expected time (min) | Safer LOC Interview | General Screening | Baseline/Pre-Op | Surgery | Post-Implant appointments | | | | | | | | | | | | | | | | | |
|---|---|---|---|---|---|---|---|---|---|---|---|---|---|---|---|---|---|---|---|---|---|---|---|
| | | | | | | Recording | | | | | | Stimulation Testing | | | | | | | | | | | |
| | | | | | 0 | 1 | 2 tc | 3 | 4 | 5 | 6 | 7 | 8 | 9 | 10 | 11 | 12 | 13 | 14 | 15 | 16 | 17 |
| Videoed informed consent process (offline ethics review by LD and LR) | 45 | ✓ | | | | | | | | | | | | | | | | | | | | |
| MacCAT-CR | 15 | ✓ | | | | | | | | | | | | | | | | | | | | |
| Eating disorder assessment | 30 | ✓ | | | | | | | | | | | | | | | | | | | | |
| EDE-Q | 10 | ✓ | | | | ✓ | | ✓ | | | ✓ | ✓ | ✓ | ✓ | ✓ | ✓ | ✓ | ✓ | ✓ | ✓ | ✓ | ✓ |
| Grilo LOC (ELOCS) | 10 | ✓ | | | | ✓ | | ✓ | | | ✓ | ✓ | ✓ | ✓ | ✓ | ✓ | ✓ | ✓ | ✓ | ✓ | ✓ | ✓ |
| Dutch Eating Behavior Questionnaire-Emotional Eating subscale | 10 | ✓ | | | | ✓ | | ✓ | | | ✓ | ✓ | ✓ | ✓ | ✓ | ✓ | ✓ | ✓ | ✓ | ✓ | ✓ | ✓ |
| Beck Depression Inventory-2 (BDI-II) | 10 | ✓ | | | | ✓ | | ✓ | | | ✓ | ✓ | ✓ | ✓ | ✓ | ✓ | ✓ | ✓ | ✓ | ✓ | ✓ | ✓ |
| C-SSRS | 10 | ✓ | | | | ✓ | | ✓ | | | ✓ | ✓ | ✓ | ✓ | ✓ | ✓ | ✓ | ✓ | ✓ | ✓ | ✓ | ✓ |
| SCID1&2 | 90 | ✓ | | | | | | | | | | | | | | | | | | | | |
| Handheld EMA intro | 15 | ✓ | | | | | | | | | | | | | | | | | | | | |
| Pregnancy Test (urine) | 1 | ✓ | | | | | | | | | | | | | | | | | | | | |
| Urine Tox Screen | 1 | ✓ | | | | | | | | | ✓ | | ✓ | | | ✓ | | | ✓ | | | ✓ |
| Body weight | 1 | ✓ | | ✓ | | ✓ | | ✓ | | | ✓ | ✓ | ✓ | ✓ | ✓ | ✓ | ✓ | ✓ | ✓ | ✓ | ✓ | ✓ |
| Vitals | 1 | ✓ | | | | ✓ | | ✓ | | | ✓ | ✓ | ✓ | ✓ | ✓ | ✓ | ✓ | ✓ | ✓ | ✓ | ✓ | ✓ |
| Fasting Glucose, Glucose Tolerance Testing | 1 | ✓ | | | | | | ✓ | | | ✓ | | ✓ | | | ✓ | | | ✓ | | | ✓ |
| Nutrition Panel (e.g. chemistry, lipids, A1C) | 1 | ✓ | | | | | | ✓ | | | ✓ | | ✓ | | | ✓ | | | ✓ | | | ✓ |
| General Demographics | 5 | ✓ | | | | | | | | | | | | | | | | | | | | |
| Review disease history | 10 | ✓ | ✓ | | | | | | | | | | | | | | | | | | | |
| Review medical history | 10 | ✓ | ✓ | | | | | | | | | | | | | | | | | | | |
| Review concomitant treatments (other meds) | 5 | ✓ | ✓ | ✓ | ✓ | ✓ | ✓ | ✓ | ✓ | ✓ | ✓ | ✓ | ✓ | ✓ | ✓ | ✓ | ✓ | ✓ | ✓ | ✓ | ✓ | ✓ |
| Adverse Event Review | 5 | | | ✓ | | ✓ | ✓ | ✓ | ✓ | ✓ | ✓ | ✓ | ✓ | ✓ | ✓ | ✓ | ✓ | ✓ | ✓ | ✓ | ✓ | ✓ |
| Nutrition appointment | 30 | | ✓ | | | | | | | | | | | | | ✓ | | | | | | ✓ |
| EMA compliance review | 10 | | ✓ | ✓ | | | | | | | | | | | | | | | | | | |
| Neurological/Physical exam | 10 | | ✓ | ✓ | | ✓ | | ✓ | | | ✓ | ✓ | ✓ | ✓ | ✓ | ✓ | ✓ | ✓ | ✓ | ✓ | ✓ | ✓ |
| Structured psychiatric interview | 30 | | ✓ | | | | | | | | | | | | | | | | | | | |
| Interventional psychiatric evaluation | 30 | | ✓ | ✓ | | ✓ | | ✓ | | ✓ | ✓ | ✓ | ✓ | ✓ | ✓ | ✓ | ✓ | ✓ | ✓ | ✓ | ✓ | ✓ |
| Neuropsychology (formal) | 60 | | ✓ | | | | | | | | | | | | | | | | | | | ✓ |
| Bariatric Monitoring Visit | 40 | | | | | | | ✓ | | | | | | | | | | | | | | |
| LOC EMA review (1 week per month of EMA) | 5 | | ✓ | ✓ | | ✓ | ✓ | ✓ | ✓ | ✓ | ✓ | ✓ | ✓ | ✓ | ✓ | ✓ | ✓ | ✓ | ✓ | ✓ | ✓ | ✓ |
| Ambulatory bite-counter intro | 10 | | ✓ | | | | | | | | | | | | | | | | | | | |
| Bite-counter review | 10 | | | ✓ | | ✓ | ✓ | ✓ | ✓ | ✓ | ✓ | ✓ | ✓ | ✓ | ✓ | ✓ | ✓ | ✓ | ✓ | ✓ | ✓ | ✓ |
| Ambulatory bite-counter intro (1 week alternating with EMA) | 10 | | ✓ | | | ✓ | ✓ | ✓ | ✓ | ✓ | ✓ | ✓ | ✓ | ✓ | ✓ | ✓ | ✓ | ✓ | ✓ | ✓ | ✓ | ✓ |
| Monetary Incentive Delay | 20 | | | ✓ | | | | | | ✓ | | ✓ | | | ✓ | | | | | | | |
| Milkshake paradigm | 20 | | | ✓ | | | | | | ✓ | | ✓ | | | ✓ | | | | | | | |
| Multi-Item Buffet (includes questionaires and nutritionist) | 360 | | | | | | | | | ✓ | | ✓ | | | ✓ | | | | | | | |
| Fidicial placement | 30 | | | ✓ | | | | | | | | | | | | | | | | | | |
| SAU preop | 30 | | | ✓ | | | | | | | | | | | | | | | | | | |
| MRI with and without contrast | 60 | | | ✓ | | | | | | | | | | | | | | | | | | |
| CT Brain | 15 | | | ✓ | ✓ | | | | | | | | | | | | | | | | | |
| Monopolar assessment | 60 | | | | ✓ | | | | | ✓ | | | | | | | | | | | | |
| Initiate recording | 5 | | | | ✓ | | | | | | | | | | | | | | | | | |
| Distribute magnet/remote monitor training | 60 | | | | ✓ | | | | | | | | | | | | | | | | | |
| Manage neurostimulator/programming | 15 | | | | ✓ | ✓ | | ✓ | ✓ | ✓ | ✓ | ✓ | ✓ | ✓ | ✓ | ✓ | ✓ | ✓ | ✓ | ✓ | ✓ | ✓ |
| Wound check | 5 | | | | | ✓ | | ✓ | | | | | | | | | | | | | | |

**9.8**

**Adverse Event Severity Rating Scale**

| | |
|---|---|
| Unrelated | No temporal relationship to study product or surgical procedure, or the presence of a reasonable causal relationship to another drug, concurrent disease, or circumstance and the adverse event (AE). |
| Unlikely | A temporal relationship to study product or surgical procedure, but no reasonable causal relationship between study product or surgical procedure and the AE. |
| Possibly | A reasonable causal relationship between the study product or surgical procedure and the AE. Information related to withdrawal of cell treatment/procedure was lacking or unclear. |
| Probably | A reasonable causal relationship between the study product or surgical procedure and the AE. The event responded to withdrawal of cell treatment/procedure. Rechallenge was not required. |
| Definitely | A reasonable causal relationship between the study product or surgical procedure and the AE. The event responded to withdrawal of the cell treatment/procedure, and recurred with re-challenge, when clinically feasible. |

# 10. APPENDIX A: BIOMARKER FUTILITY PHASE

| Responsive Neurostimulation for Loss of Control Eating | Expected time (min) | LOC Interview | General Screening | Baseline/Pre-Op | Surgery | Recording | | | | | | Only if No Biomarker Detected: Extended Recording | | | Only if No Biomarker Detected: IDE/IRB Supplement | | | Only if No Biomarker Detected: Novel Software Testing | | | Stimulation Testing | | | | | | | | | | | |
|---|---|---|---|---|---|---|---|---|---|---|---|---|---|---|---|---|---|---|---|---|---|---|---|---|---|---|---|---|---|---|---|---|
| | | | | | 0 | 1 | 2 tc | 3 | 4 | 5 | 6 | 7 tc | 8 | 9 | 10 tc | 11 tc | 12 tc | 13 | 14 | 15 | 16 | 17 | 18 | 19 | 20 | 21 | 22 | 23 | 24 | 25 | 26 | 27 |
| Videoed informed consent process | 45 | ✓ | | | | | | | | | | | | | | | | | | | | | | | | | | | | | | |
| MacCAT-CR | 15 | ✓ | | | | | | | | | | | | | | | | | | | | | | | | | | | | | | |
| Eating disorder assessment | 30 | ✓ | | | | | | | | | | | | | | | | | | | | | | | | | | | | | | |
| EDE-Q | 10 | ✓ | | | | ✓ | | ✓ | | | ✓ | | | ✓ | | | | ✓ | ✓ | ✓ | ✓ | ✓ | ✓ | ✓ | ✓ | ✓ | ✓ | ✓ | ✓ | ✓ | ✓ | ✓ |
| Grilo LOC (ELOCS) | 10 | ✓ | | | | ✓ | | ✓ | | | ✓ | | | ✓ | | | | ✓ | ✓ | ✓ | ✓ | ✓ | ✓ | ✓ | ✓ | ✓ | ✓ | ✓ | ✓ | ✓ | ✓ | ✓ |
| Dutch Eating Behavior Questionnaire-Emotional Eating subscale | 10 | ✓ | | | | ✓ | | ✓ | | | ✓ | | | ✓ | | | | ✓ | ✓ | ✓ | ✓ | ✓ | ✓ | ✓ | ✓ | ✓ | ✓ | ✓ | ✓ | ✓ | ✓ | ✓ |
| Beck Depression Inventory-2 (BDI-II) | 10 | ✓ | | | | ✓ | | ✓ | | | ✓ | | | ✓ | | | | ✓ | ✓ | ✓ | ✓ | ✓ | ✓ | ✓ | ✓ | ✓ | ✓ | ✓ | ✓ | ✓ | ✓ | ✓ |
| C-SSRS | 10 | ✓ | | | | ✓ | | ✓ | | | ✓ | | | ✓ | | | | ✓ | ✓ | ✓ | ✓ | ✓ | ✓ | ✓ | ✓ | ✓ | ✓ | ✓ | ✓ | ✓ | ✓ | ✓ |
| SCID1&2 | 90 | ✓ | | | | | | | | | | | | | | | | | | | | | | | | | | | | | | |
| EMA intro | 15 | ✓ | | | | | | | | | | | | | | | | | | | | | | | | | | | | | | |
| Pregnancy Test (urine) | 1 | ✓ | | | | | | | | | | | | | | | | | | | | | | | | | | | | | | |
| Urine Tox Screen | 1 | ✓ | | | | | | | | | ✓ | | | | | | | ✓ | | ✓ | | | ✓ | | | ✓ | | | ✓ | | | ✓ |
| Body weight | 1 | ✓ | | ✓ | | ✓ | | ✓ | | | ✓ | | | ✓ | | | | ✓ | ✓ | ✓ | ✓ | ✓ | ✓ | ✓ | ✓ | ✓ | ✓ | ✓ | ✓ | ✓ | ✓ | ✓ |
| Vitals | 1 | ✓ | | ✓ | | ✓ | | ✓ | | | ✓ | | | ✓ | | | | ✓ | ✓ | ✓ | ✓ | ✓ | ✓ | ✓ | ✓ | ✓ | ✓ | ✓ | ✓ | ✓ | ✓ | ✓ |
| Fasting Glucose, Glucose Tolerance Testing | 1 | ✓ | | | | | | ✓ | | | ✓ | | | ✓ | | | | | | ✓ | | | ✓ | | | ✓ | | | ✓ | | | ✓ |
| Nutrition Panel (e.g. chemistry, lipids, A1C) | 1 | ✓ | | | | | | ✓ | | | ✓ | | | ✓ | | | | | | ✓ | | | ✓ | | | ✓ | | | ✓ | | | ✓ |
| General Demographics | 5 | ✓ | | | | | | | | | | | | | | | | | | | | | | | | | | | | | | |
| Review disease history | 10 | ✓ | ✓ | ✓ | ✓ | | | | | | | | | | | | | | | | | | | | | | | | | | | |
| Review medical history | 10 | ✓ | ✓ | ✓ | ✓ | | | | | | | | | | | | | | | | | | | | | | | | | | | |
| Review concomitant treatments (other meds) | 5 | ✓ | ✓ | ✓ | ✓ | ✓ | ✓ | ✓ | ✓ | ✓ | ✓ | ✓ | ✓ | ✓ | ✓ | ✓ | ✓ | ✓ | ✓ | ✓ | ✓ | ✓ | ✓ | ✓ | ✓ | ✓ | ✓ | ✓ | ✓ | ✓ | ✓ | ✓ |
| Adverse Event Review | 5 | | | ✓ | | ✓ | ✓ | ✓ | ✓ | ✓ | ✓ | ✓ | ✓ | ✓ | ✓ | ✓ | ✓ | ✓ | ✓ | ✓ | ✓ | ✓ | ✓ | ✓ | ✓ | ✓ | ✓ | ✓ | ✓ | ✓ | ✓ | ✓ |
| Nutrition appointment | 30 | | ✓ | | | | | | | | | | | | | | | | | | | | | | | ✓ | | | | | | ✓ |
| EMA compliance review | 10 | | ✓ | | | | | | | | | | | | | | | | | ✓ | | | | | | | | | | | | |
| Neurological/Physical exam | 10 | | ✓ | ✓ | | ✓ | | ✓ | | | ✓ | | | ✓ | | | | ✓ | ✓ | ✓ | ✓ | ✓ | ✓ | ✓ | ✓ | ✓ | ✓ | ✓ | ✓ | ✓ | ✓ | ✓ |
| Structured psychiatric interview | 30 | | ✓ | | | | | | | | | | | | | | | | | ✓ | | | | | | | | | | | | |
| Interventional psychiatric evaluation | 30 | | ✓ | ✓ | | ✓ | | ✓ | | ✓ | ✓ | | ✓ | ✓ | ✓ | ✓ | ✓ | ✓ | ✓ | ✓ | ✓ | ✓ | ✓ | ✓ | ✓ | ✓ | ✓ | ✓ | ✓ | ✓ | ✓ | ✓ |
| Neuropsychology (formal) | 60 | | ✓ | | | | | | | | | | | | | | | | | ✓ | | | | | | | | | | | | ✓ |
| Bariatric Monitoring Visit | 40 | | | | | | | ✓ | | | | | | | | | | | | | | | | | | | | | | | | |
| LOC EMA review (1 week per month of EMA) | 5 | | ✓ | ✓ | | ✓ | ✓ | ✓ | ✓ | ✓ | ✓ | ✓ | ✓ | ✓ | | | | ✓ | ✓ | ✓ | ✓ | ✓ | ✓ | ✓ | ✓ | ✓ | ✓ | ✓ | ✓ | ✓ | ✓ | ✓ |
| Bite-counter intro | 10 | | ✓ | | | | | | | | | | | | | | | | | | | | | | | | | | | | | |
| Bite-counter compliance review | 10 | | | ✓ | | | | | | | | | | | | | | | | ✓ | | | | | | | | | | | | |
| Ambulatory bite-counter (1 week alternating with EMA) | 10 | | | ✓ | | ✓ | ✓ | ✓ | ✓ | ✓ | ✓ | ✓ | ✓ | ✓ | | | | ✓ | ✓ | ✓ | ✓ | ✓ | ✓ | ✓ | ✓ | ✓ | ✓ | ✓ | ✓ | ✓ | ✓ | ✓ |
| Monetary Incentive Delay | 20 | | | ✓ | | | | | | ✓ | | | ✓ | | | | | | ✓ | | ✓ | | | ✓ | | | | | | | | |
| Milkshake paradigm | 20 | | | ✓ | | | | | | ✓ | | | ✓ | | | | | | ✓ | | ✓ | | | ✓ | | | | | | | | |
| Multi-Item Buffet (includes questionaires and nutritionist) | 360 | | | | | | | | | ✓ | | | ✓ | | | | | | ✓ | | ✓ | | | ✓ | | | | | | | | |
| Fidicial placement | 30 | | | ✓ | | | | | | | | | | | | | | | | | | | | | | | | | | | | |
| SAU preop | 30 | | | ✓ | | | | | | | | | | | | | | | | | | | | | | | | | | | | |
| MRI with and without contrast | 60 | | | ✓ | | | | | | | | | | | | | | | | | | | | | | | | | | | | |
| CT Brain | 15 | | | ✓ | ✓ | | | | | | | | | | | | | | | | | | | | | | | | | | | |
| Monopolar assessment | 60 | | | | ✓ | | | | | ✓ | | | | | | | | | | | ✓ | | | | | | | | | | | |
| Initiate recording | 5 | | | | ✓ | | | | | | | | | | | | | | | | | | | | | | | | | | | |
| Distribute magnet/remote monitor training | 60 | | | | ✓ | | | | | | | | | | | | | | | | | | | | | | | | | | | |
| Manage neurostimulator/programming | 15 | | | | ✓ | ✓ | | ✓ | ✓ | ✓ | ✓ | | ✓ | ✓ | | | | ✓ | ✓ | ✓ | ✓ | ✓ | ✓ | ✓ | ✓ | ✓ | ✓ | ✓ | ✓ | ✓ | ✓ | ✓ |
| Wound check | 5 | | | | | ✓ | | ✓ | | | | | | | | | | | | | | | | | | | | | | | | |

## 10.0 Summary of futility phase:

If no biomarker is identified during the initial 6 month recording phase in any of the subjects, additional measures will occur following the Schedule of Events noted in Appendix A. An additional informed consent document will be provided to any subject requiring additional testing outlying the additional procedures. No procedures will be performed or retesting without subject consent and approval from the FDA is received.

### 10.1 Extended Recording for troubleshooting:

After confirmation that no LFP biomarker was identified, detection algorithms will be modified within the currently approved settings and retesting will occur for an additional 3 months. Safety assessments will continue to be assessed during this time as well.

### 10.2 Novel Software Testing (3 months):

If after the extended recording, no detected quantifiable LFP biomarker is identified, a software update will occur. This 6-month period will include the updating of the software and retesting. Updating the software will not increase risk to subjects as the new software can be sent directly to the device through wireless telemetry. During the software update and retesting, safety assessments will continue to be assessed as defined by the SOE. All subsequent devices would be updated prior to implant.

### 10.3 Self-directed Stimulation:

In the absence of a well-defined biomarker associated with LOC eating, self-directed magnet triggering of stimulation to the NAc will occur for 6 months. Subjects will be limited in the number of times per day that they can magnet trigger for stimulation based on EMA data and prior magnet swipe timestamp data.

### 10.4 Scheduled Stimulation:

Following self-directed magnet triggered stimulation, scheduled stimulation will occur for 6 months. Scheduled stimulation is determined based on the schedule of LOC behavior and timing of stimulation delivery captured in the electrographic dataset of each individual subject.

### 10.5Futility:

hould an electrographic biomarker not be identified after these steps, no additional subjects will be implanted without an IDE supplement and FDA approval. Non-futility will be defined as a reduction in #LOC episodes/week from the period prior to initiating stimulation to study completion in 50% of the patients (we will define baseline as the #LOC episodes/week during the month prior to stimulation initiation). A statistically significant difference is not expected. Rationale: The purpose of this milestone is to demonstrate non-

futility based on improvement in #LOC episodes/week. We anticipate that intermittent NAc stimulation will attenuate LOC, but we believe it is essential to initially strike a balance between being overly conservative for this EFS (in which the magnitude and time course for improvement have not previously been studied) and being clinically insignificant. Should an increase be seen in 50% of these subjects, there will be no further advancement of this intervention as studied in this protocol.
