# Supplementary material for: Pilot study of responsive nucleus accumbens deep brain stimulation for loss-of-control eating
Source: Nat Med. 2022 Aug 29;28(9):1791–6. doi: 10.1038/s41591-022-01941-w (PMC9499853; doi:10.1038/s41591-022-01941-w)
Supplement: Supplementary file 2 — Reporting Summary [file 41591_2022_1941_MOESM2_ESM.pdf]

nature research

Corresponding author(s): Casey Halpern

Last updated by author(s): Jun 26, 2022

Reporting Summary

Nature Research wishes to improve the reproducibility of the work that we publish. This form provides structure for consistency and transparency in reporting. For further information on Nature Research policies, see our Editorial Policies and the Editorial Policy Checklist.

Statistics

For all statistical analyses, confirm that the following items are present in the figure legend, table legend, main text, or Methods section.

n/a	Confirmed	
☐	☒	The exact sample size (*n*) for each experimental group/condition, given as a discrete number and unit of measurement
☐	☒	A statement on whether measurements were taken from distinct samples or whether the same sample was measured repeatedly
☐	☒	The statistical test(s) used AND whether they are one- or two-sided<br>*Only common tests should be described solely by name; describe more complex techniques in the Methods section.*
☒	☐	A description of all covariates tested
☐	☒	A description of any assumptions or corrections, such as tests of normality and adjustment for multiple comparisons
☐	☒	A full description of the statistical parameters including central tendency (e.g. means) or other basic estimates (e.g. regression coefficient) AND variation (e.g. standard deviation) or associated estimates of uncertainty (e.g. confidence intervals)
☐	☒	For null hypothesis testing, the test statistic (e.g. F, t, r) with confidence intervals, effect sizes, degrees of freedom and P value noted<br>*Give P values as exact values whenever suitable.*
☒	☐	For Bayesian analysis, information on the choice of priors and Markov chain Monte Carlo settings
☒	☐	For hierarchical and complex designs, identification of the appropriate level for tests and full reporting of outcomes
☒	☐	Estimates of effect sizes (e.g. Cohen's d, Pearson's r), indicating how they were calculated

Our web collection on statistics for biologists contains articles on many of the points above.

Software and code

Policy information about availability of computer code

Data collection	Electrophysiologic data was obtained from intracranially placed depth leads by using commercially available Neuropace RNS system
Data analysis	All data analyses were performed in (R2014b, The Mathworks Inc., MA) using custom scripts built upon the FieldTrip Toolbox (11; Donders Institute for Brain, Cognition and Behaviour, Radboud University, the Netherlands. See http://fieldtriptoolbox.org). No new algorithm or pre-processing techniques were performed outside of standard toolbox usage. The code for analyzing the data of this study are available from the corresponding author on reasonable request. The electrode artifacts from the co-registered post-operative CT, together with the pre-operative MRI, and atlas-based regions of interest were then loaded in DSI Studio for 3D rendering (https://dsi-studio.labsolver.org/). The pre-operative MRI anatomical images were co-registered to post-surgical CT scan using Advanced Normalization Tools (ANTs [20]) for electrode localization [21]

For manuscripts utilizing custom algorithms or software that are central to the research but not yet described in published literature, software must be made available to editors and reviewers. We strongly encourage code deposition in a community repository (e.g. GitHub). See the Nature Research guidelines for submitting code & software for further information.

Data

Policy information about availability of data

All manuscripts must include a data availability statement. This statement should provide the following information, where applicable:

- Accession codes, unique identifiers, or web links for publicly available datasets
- A list of figures that have associated raw data
- A description of any restrictions on data availability

The data that support the findings of this study are available upon reasonable request to the corresponding author [CH]. The data are not publicly available due to them containing information that could compromise research participant privacy/consent. As this is part of an ongoing clinical trial, enrolling additional subjects

(Trial Registration # NCT03868670), all data and code will be deposited into Data Archive Brain Initiative (https://dabi.loni.usc.edu) as part of the BRAIN Initiative at the completion of the study. During this time, any request will be reviewed in a timely manner by the corresponding author, corresponding author's institution, and ultimately shared within reason of a signed data transfer agreement. All code has been made publicly available, and can be found https://github.com/rajatss123/LOC_NM_Code.

Field-specific reporting

Please select the one below that is the best fit for your research. If you are not sure, read the appropriate sections before making your selection.

☒ Life sciences ☐ Behavioural & social sciences ☐ Ecological, evolutionary & environmental sciences

For a reference copy of the document with all sections, see nature.com/documents/nr-reporting-summary-flat.pdf

Life sciences study design

All studies must disclose on these points even when the disclosure is negative.

Sample size	The sample was 2 patients, the first 2 of a N=6 clinical study. No sample size calculation was made. Two was sufficient in that it was the first two patients declared as a pilot sample in an N=6 clinical trial. As this is an early feasibility study assessing safety, per FDA guidelines subjects enrolled in the study are staggered and in cohort of 2 before the next cohort can be enrolled with FDA approval.
Data exclusions	No data was excluded from this manuscript.
Replication	Data was replicated between two patients, the samples from which were sufficiently powered.
Randomization	No randomization occurred during the recording phase. One subject (Subject 1), per the FDA-guided protocol, was randomized to a SHAM stimulation safety period. For the buffet task (see supplementary information for more details), we presented a standard caloric meal (lunch or breakfast) prior to presenting the LOC buffet. During the LOC buffet, when subject reported they were losing control, the buffet was stopped within 10 minutes per protocol. For ambulatory data, in addition to self-report LOC (magnet swiping, see supplementary info), subjects were asked to swipe magnets during standard meals as well as during period when they were relaxed, not eating. All ambulatory LFP recordings were captured in the same configuration.
Blinding	The purpose of this trial is to assess the safety and feasibility of this potential therapeutic approach. Thus, prior to the stimulation phase subjects 1) underwent acute monopolar testing in clinic to assess for stimulation side effects and therapeutic parameters, and 2) underwent a safety single-blinded ON/OFF period in which subjects were sent home for 1 week stim ON, then 1 week stim OFF, then 4 weeks ON, followed by 1 week OFF before entering the unblinded open label phase. This was crucial to assess for stimulation-induced side effects and was part of the protocol design approved by the FDA (Wu et al, 2020). As this is a first-in-human early feasibility trial, primarily designed to assess safety/feasibility, we did not incorporate a double-blinded protocol which is typically used to assess efficacy.<br><br>Further, for this 2-subject cohort, per the FDA-guided protocol, Subject 1 additionally was randomized to a SHAM period in which the above Safety period was assessed but with stimulation OFF the entire period. During this time everyone involved in the study, except the programming clinician, was blinded. This period occurred right before participants entered the open-label phase of the study.

Reporting for specific materials, systems and methods

We require information from authors about some types of materials, experimental systems and methods used in many studies. Here, indicate whether each material, system or method listed is relevant to your study. If you are not sure if a list item applies to your research, read the appropriate section before selecting a response.

Materials & experimental systems

n/a	Involved in the study	
☒	☐	Antibodies
☒	☐	Eukaryotic cell lines
☒	☐	Palaeontology and archaeology
☒	☐	Animals and other organisms
☐	☒	Human research participants
☐	☒	Clinical data
☒	☐	Dual use research of concern

Methods

n/a	Involved in the study	
☒	☐	ChIP-seq
☒	☐	Flow cytometry
☒	☐	MRI-based neuroimaging

Human research participants

Policy information about studies involving human research participants

Population characteristics	The first participant (Subject 1), a 45 year-old Caucasian woman with a body mass index (BMI) of 46 kg/m2, underwent Roux-en-Y gastric bypass (RYGB) in 2005 and experienced an initial weight loss of 115 lbs. However, the participant gradually

	regained the weight. At the time of enrollment, she was back to her pre-RYGB weight and met DSM-5 criteria for binge eating disorder, reporting at least 5 LOC eating bouts per week. At the time of enrollment, she had co-morbities of Neoplasm, lower back pain, kyphoscoliosis/scoliosis, hypertension, esophageal reflux, dyslipidemia, complicated migraine, and anxiety. The second participant (Subject 2), a 56 year-old Caucasian woman with a BMI of 47 kg/m2, underwent RYGB in 2005 and initially lost 152 lbs. Subject 2 maintained this weight loss for 6 years; however, she regained the weight in 2009 while both caring for an ill family member and recovering from a car accident. At the time of study enrollment, she was within 9% of her pre-RYGB weight and she reported LOC eating at least 4 times a week and met criteria for binge eating disorder as well. At the time of enrollment, she had a migraine co-morbidity. Both subjects reported severe cravings related to emotional and/or stress-related triggers that led to LOC eating. In addition to the RYGB, both subjects tried numerous other weight loss strategies including exercise, dieting, support groups, and medication as was required by our enrollment criteria.
Recruitment	Subjects were recruited through Stanford Bariatric Clinic, Newspaper (print and online), Facebook, or ResearchMatch. Participant bias is present as they are the ones self-reporting LOC severity (e.g. craving, hunger level). Over time, subjects became more aware of what was an LOC event and what was not. Participant bias as it relates to recruitment was present in that only those subjects deeming themselves at a symptom severity level worthy of this type of invasive intervention were responding to the ads for this study.
Ethics oversight	This study was approved by Stanford's Institutional Review Board (IRB-46563), and informed consent was obtained on all subjects. Neither participant was compensated for their participation.

Note that full information on the approval of the study protocol must also be provided in the manuscript.

Clinical data

Policy information about clinical studies

All manuscripts should comply with the ICMJE guidelines for publication of clinical research and a completed CONSORT checklist must be included with all submissions.

Clinical trial registration	Trial Registration # NCT03868670
Study protocol	Implantation pf rDBS was performed for Subject 1 on January 31 2020, and for Subject 2 on July 17 2020. Timeline of procedures for both these subjects can be matched to these dates and expected timeline. The full study protocol can be accessed as part of this manuscript, and can be found here: https://www.ncbi.nlm.nih.gov/pmc/articles/PMC8599841/
Data collection	Data reported was collected on site on the fourth floor of Stanford University's Psychiatry Building at 401 Quarry Road, Palo Alto, CA. Data reported from ambulatory assessments was collected in the given patients naturalistic environment, collected in real-world settings.
Outcomes	Primary outcomes were LOC frequency and severity. These will both be acquired using the Ecological Momentary Assessment Questionnaires. Participants are asked to rate LOC immediately before and after any eating episode. Ratings for pre- meal LOC are made on a 5-point Likert-type scale. A rating of 1 on the scale corresponds to "complete control" and 5 signifies "complete LOC"; post-meal, participants respond to a "yes/no" question as to whether they had experienced LOC while eating. In addition, participants will be signaled at semi-random intervals several times a day to ask retrospectively about any episodes of LOC that may have taken place since the last signal. If the participant did not already fill out ratings about that episode, they are asked to do so at that time. In addition, participants are asked to fill out an end-of-the-day record to ensure that any and all LOC episodes are recorded. Assessment of LOC severity will involve several types of recordings to maximize the ability to capture episodes of LOC. These will include signal, interval contingent, and behavior contingent recordings. Signal contingent recording includes having the participant record their experiences whenever signaled at semi- random times by the researcher. In this study, participants will be signaled 5 semi- random times per day. Each response will require 2-3 minutes to complete. Compliance will be assessed by how often patients respond to signaled recordings (minimum inclusion criteria of 80% compliance). Behavior contingent recordings refer to having participants complete recordings before and after they engage in behaviors rather than in response to a text. Interval contingent recording includes recording at regular intervals determined ahead of time by the researcher. In this study, participants will complete an end of day recording as well. During monthly follow-ups, they will be queried about the details of each eating episode as well. All data will be automatically time- and date-stamped on smartphones and saved in a secure online database, providing the opportunity to delineate temporal relationships with LFP recordings and concordance with other assessments. Additionally, monthly reports of LOC severity using the Grilo Eating Loss of Control Scale (ELOCS) were obtained. The ELOCS is a self-report questionnaire that examines LOC-related behaviors on continuous Likert-type scales and the number of LOC episodes in the past 28 days. Given that LOC is a central diagnostic and clinical feature of multiple eating disorders, this comprehensive, validated, self-report measure of LOC was created to capture the varied experience of LOC among individuals by measuring different aspects of this construct as well as severity on continuous Likert- type scales. This scale does not diagnose the presence of LOC – this is done dichotomously (yes/no) and during a structured clinical interview for an eating disorder assessment, facilitated by the EDE-Q and ELOCS described above.<br><br>Secondary outcomes were weight, BMI, and reported personality measures, obtained monthly. Personality is measured using the Ecological Momentary Assessment Questionnaires. Participants are asked to rate mood, stress, hunger, and LOC immediately before and after any eating episode. Negative mood is assessed using an abbreviated Positive and Negative Affect Scale (PANAS) as LOC eating is typically preceded by negative emotion. This scale comprises the sum of 5 items (afraid, scared, upset, distressed, and jittery), all of which are rated on a 5-point scale. Weight and BMI were obtained monthly, assessed and reported to the clinical team by the subject themselves.
